# Prenatal phthalate exposure and cord blood DNA methylation

**DOI:** 10.1038/s41598-023-33002-8

**Published:** 2023-04-29

**Authors:** Jooah Lee, Jeeyoung Kim, Sabrina Shafi Zinia, Jaehyun Park, Sungho Won, Woo Jin Kim

**Affiliations:** 1 Department of Public Health Sciences, Seoul National University, Seoul, South Korea; 2 Department of Internal Medicine and Environmental Health Center, School of Medicine, Kangwon National University, Chuncheon, 24341 South Korea; 3 Interdisciplinary Program of Bioinformatics, College of Natural Sciences, Seoul National University, Seoul, 08826 South Korea; 4 Institute of Health and Environment, Seoul National University, Seoul, South Korea; 5 RexSoft Corp, Seoul, South Korea

**Keywords:** Computational biology and bioinformatics, Genetics

## Abstract

Exposure to phthalates has been shown to impede the human endocrine system, resulting in deleterious effects on pregnant women and their children. Phthalates modify DNA methylation patterns in infant cord blood. We examined the association between prenatal phthalate exposure and DNA methylation patterns in cord blood in a Korean birth cohort. Phthalate levels were measured in 274 maternal urine samples obtained during late pregnancy and 102 neonatal urine samples obtained at birth, and DNA methylation levels were measured in cord blood samples. For each infant in the cohort, associations between CpG methylation and both maternal and neonate phthalate levels were analyzed using linear mixed models. The results were combined with those from a meta-analysis of the levels of phthalates in maternal and neonatal urine samples, which were also analyzed for MEOHP, MEHHP, MnBP, and DEHP. This meta-analysis revealed significant associations between the methylation levels of CpG sites near the *CHN2* and *CUL3* genes, which were also associated with MEOHP and MnBP in neonatal urine. When the data were stratified by the sex of the infant, MnBP concentration was found to be associated with one CpG site near the *OR2A2* and *MEGF11* genes in female infants. In contrast, the concentrations of the three maternal phthalates showed no significant association with CpG site methylation. Furthermore, the data identified distinct differentially methylated regions in maternal and neonatal urine samples following exposure to phthalates. The CpGs with methylation levels that were positively associated with phthalate levels (particularly MEOHP and MnBP) were found to be enriched genes and related pathways. These results indicate that prenatal phthalate exposure is significantly associated with DNA methylation at multiple CpG sites. These alterations in DNA methylation may serve as biomarkers of maternal exposure to phthalates in infants and are potential candidates for investigating the mechanisms by which phthalates impact maternal and neonatal health.

## Introduction

Phthalates are classified as endocrine-disrupting chemicals (EDCs) and are commonly found in consumer products such as food packaging and toys^1^. Owing to their molecular structure, phthalates undergo hydrolysis into monoesters, which in turn become secondary metabolites^2,3^. Accumulating evidence suggests that constant exposure to phthalates can permanently modify the normal physiology of hormone-mediated processes, such as those involved in metabolism, reproductive function, immune response, energy balance, and neurological function^4^. During the prenatal and early postnatal periods, infants are particularly vulnerable to phthalate exposure because it is during this time that developmental programming defines organogenesis, fetal development, birth weight, early childhood behavior, and tissue differentiation. As a result, early life exposure to phthalates leads to adverse health outcomes, including obesity, allergic diseases, cardiovascular disease, and respiratory issues^5–9^.

DNA methylation is tightly controlled during human development, particularly during the early stages of egg fertilization^10^. Long-term exposure to phthalates during early life is associated with epigenetic changes^10–12^, including DNA methylation and histone modification. These changes affect gene expression and function, increasing susceptibility to several diseases. Growing evidence suggests that alterations in DNA methylation at birth underlie the association between prenatal phthalate exposure and adverse health outcomes. Genes affected by changes in DNA methylation as a result of phthalate exposure were found to be enriched in gene sets or pathways involved in cell cycle regulation, such as mitotic spindle-related genes, E2F-target genes, Myc-target genes, and p53 signaling genes^13–15^, all of which are associated with the effects of phthalates on health, such as reduced cell or organ development. To date, in most of these studies^13–23^, variations in the research design can be categorized into five key principles: first, whether phthalates are classified according to low molecular weights (LMW, e.g., DMP, DEP, DBP, and DiBP) and high molecular weights (HMW, e.g., DEHP, DiNP, and DPHP); second, the type of urinalysis used to measure prenatal exposure in the early to late stages of pregnancy; third, the type of sample (e.g., placenta or cord blood) in which infant DNA methylation was analyzed; fourth, the method used to profile DNA methylation (e.g., in candidate genes or epigenome-wide); and fifth, whether the study population was stratified by the sex of the infant. This last factor is important since the effects of EDCs may be sexually dimorphic or related to the alteration of sex-specific hormone levels (such as thyroid hormones, estrogen, and testosterone)^4,16^. Enrichment of the androgen-responsive gene set could explain why phthalates have anti-androgenic properties, while those of protein secretion and glycolysis-related gene sets may be linked to allergic symptoms, behavioral difficulties, and decreased levels of sex hormones^4^.

Although cohort studies in humans have shown an association between maternal phthalate exposure and DNA methylation changes in cord blood samples^13–20^, the results have been inconsistent, likely because of differences in epigenetic profiles according to race or ethnicity, sex, and age. More research is needed to gain, insight into the mechanisms of the effects of prenatal phthalate exposure is important for developing prevention strategies to mitigate phthalate-associated health outcomes. In the present study, we investigated the differences in cord blood DNA methylation patterns and the association between these differences and prenatal phthalate exposure in a Korean birth cohort. All the analyses on infants were conducted in a sex-specific manner.

## Results

### Subject characteristics

The subject characteristics of the mothers and the newborns for each center are presented in the following order: Ulsan, Ewha, and Dankook. The sample sizes for the maternal group were 159, 87, and 28, and those for the birth group were 74, 14, and 14, respectively. The average BMIs before pregnancy in the maternal group were 21.97 kg/m^2^ (standard error (SE) = 0.25 kg/m^2^), 22.42 kg/m^2^ (SE = 0.34 kg/m^2^), and 21.48 kg/m^2^ (SE = 0.41 kg/m^2^), respectively. In the birth group, the average BMIs were 22.38 kg/m^2^ (SE = 0.36 kg/m^2^), 22.97 kg/m^2^ (SE = 0.99 kg/m^2^), and 21.39 kg/m^2^ (SE = 0.73 kg/m^2^), respectively. The number of current smokers in the maternal group was 3, 1, and 1, respectively, and there were no current smokers among the matched mothers for birth subjects in the birth group. In the birth group, the number of males was 43, 4, and 6, and for females, the numbers were 31, 10, and 8, respectively (Table 1).

**Table 1:** Summary statistics for Ulsan, Ewha, and Dankook centers for maternal and birth group samples. Center-specific sample sizes for each group and their percentages are shown. For binary covariates, the respective count and percentage are shown. For continuous covariates, the average values are shown with standard errors in parentheses.

Group	Maternal group			Birth group		
Center	Ulsan	Ewha^a^	Dankook	Ulsan	Ewha	Dankook
Sample size	159 (58.03%)	87 (31.75%)	28 (10.22%)	74 (72.55%)	14 (13.73%)	14 (13.73%)
Covariates						
Sex of newborn subject						
Male	81 (50.94%)	46 (52.87%)	14 (50.00%)	43 (58.11%)	4 (28.57%)	6 (42.86%)
Female	78 (49.06%)	41 (47.13%)	14 (50.00%)	31 (41.89%)	10 (71.43%)	8 (57.14%)
Pre-pregnancy BMI (kg/m^2^)	21.97 (0.25)	22.42 (0.34)	21.48 (0.41)	22.38 (0.36)	22.97 (0.99)	21.39 (0.73)
Current smokers^b^	3 (1.89%)	1 (1.15%)	1 (3.57%)	0 (0.00%)	0 (0.00%)	0 (0.00%)
Average monthly household income	2.11 (0.08)	2.72 (0.12)	2.25 (0.22)	2.01 (0.11)	2.71 (0.27)	2.00 (0.30)
Estimated leukocyte compositions (%)						
CD8 T cells	0.05 (0.00)	0.04 (0.00)	0.04 (0.01)	0.05 (0.00)	0.03 (0.01)	0.03 (0.00)
CD4 T cells	0.22 (0.01)	0.19 (0.01)	0.19 (0.02)	0.22 (0.01)	0.21 (0.03)	0.22 (0.02)
Natural killer cells	0.03 (0.00)	0.03 (0.00)	0.03 (0.00)	0.04 (0.00)	0.03 (0.00)	0.03 (0.00)
B cells	0.05 (0.00)	0.06 (0.00)	0.05 (0.00)	0.05 (0.00)	0.05 (0.01)	0.06 (0.01)
Monocytes	0.09 (0.00)	0.10 (0.00)	0.10 (0.01)	0.08 (0.00)	0.09 (0.01)	0.11 (0.01)
Granulocytes	0.55 (0.01)	0.58 (0.01)	0.57 (0.02)	0.55 (0.01)	0.59 (0.03)	0.54 (0.04)
Nucleated red blood cells	0.04 (0.00)	0.02 (0.00)	0.03 (0.01)	0.03 (0.00)	0.02 (0.01)	0.02 (0.01)

^a^One maternal sample from the Ewha Center was not assessed for MnBP concentration; thus, the corresponding data were excluded from the analysis. The data shown are relative to 274 subjects, including one subject without MnBP concentration.

^b^Smokers refer to the matched mothers for newborn subjects in the birth group.

### Phthalate exposure

In the maternal group, urine samples from 274 subjects were used to estimate MEOHP, MEHHP, and DEHP levels, and urine samples from 273 subjects were used to estimate MnBP levels. The geometric mean values of the MEOHP, MEHHP, MnBP, and DEHP concentrations were 12.49 µg/L (min. = 0.28; max. = 366.85), 11.59 µg/L (0.24; 271.72), 30.36 µg/L (0.22; 2168.70), and 24.47 µg/L (0.52;638.57), respectively. In the birth group, 102 urine samples were analyzed to estimate the concentrations of the four phthalates. The geometric mean values of the exposure variables were 4.59 µg/L (0.28; 152.91) for MEOHP, 2.79 µg/L (0.22; 114.09) for MEHHP, 20.52 µg/L (0.22; 381.68) for MnBP, and 7.56 µg/L (0.52;267.00) for DEHP.

**Figure 1:**
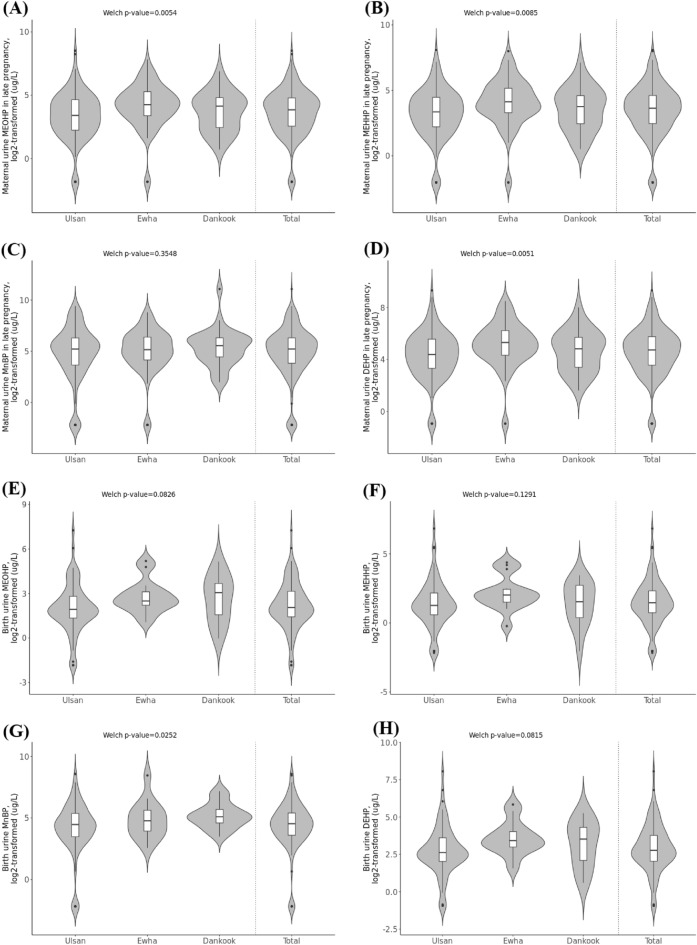
Violin plots of log_2_-transformed (**A**) maternal urine MEOHP concentrations during late pregnancy, (**B**) birth urine MEOHP concentrations, (**C**) maternal urine MEHHP concentrations during late pregnancy, (**D**) birth urine MEHHP concentrations, (**E**) maternal urine MnBP concentrations during late pregnancy, (**F**) birth urine MnBP concentrations, (**G**) maternal urine DEHP concentrations during late pregnancy, (**H**) birth urine DEHP concentrations. Plots for each of the three centers, namely, Ulsan, Ewha, and Dankook, and for the total samples are shown; *p*-values of Welch’s one-way test on the phthalate concentrations for the three centers are provided.

Center-specific phthalate levels were also analyzed. The geometric mean values are described in the following order: Ulsan, Ewha, and Dankook. The mean values of MEOHP concentrations in the maternal group were 10.22 µg/L, 17.82 µg/L, and 12.88 µg/L; in the birth group, they were 4.00 µg/L, 6.92 µg/L, and 6.25 µg/L. The mean values of MEHHP concentrations were 9.50 µg/L, 16.55 µg/L, and 11.87 µg/L in the maternal group samples and 2.58 µg/L, 4.55 µg/L, and 2.63 µg/L in the birth group samples. The mean values of MnBP concentrations were 27.52 µg/L, 32.98 µg/L, and 41.12 µg/L in the maternal group and 17.26 µg/L, 28.92 µg/L, and 36.29 µg/L for the birth group. Finally, the mean values for DEHP concentrations in the maternal group samples were 19.98 µg/L, 35.13 µg/L, and 25.12 µg/L and in the birth group were 6.69 µg/L, 11.93 µg/L, and 9.16 µg/L. To test for differences in exposure levels among the three centers, Welch’s one-way test^24^ was performed on the log_2_-transformed data. For maternal group subjects, the test results for the concentrations of MEOHP, MEHHP, MnBP, and DEHP had *p*-values of 5.4E-03, 8.5E-03, 3.5E-01, and 5.1E-03, respectively, and for birth group subjects, the *p*-values were 8.3E-02, 1.3E-01, 2.5E-02, and 8.2E-02, respectively. Therefore, a meta-analysis was performed to combine the results from the three centers. The center-specific and total violin plots as well as the *p*-values of Welch’s one-way test are shown in Fig. 1.

### Detection of differentially methylated positions (DMPs)

After quality control, 793,925 CpGs were tested for their association with phthalate concentrations. Based on the FDR-adjusted 0.05 significance level using the Benjamini–Hochberg method and absolute value of weighted beta over 0.1, no CpGs were significantly associated with the four phthalate concentrations in the maternal group samples. Under the same threshold, cg12469381 and cg11229715 had a significant association with MEOHP and MnBP concentrations in the birth group samples, respectively. The center-specific and meta-combined regression coefficients, *p*-values, and annotation results are shown in Table 2. EWAS for DEHP exposure in the birth group also revealed one significant CpG, cg16419298, under the FDR-adjusted significance level of 0.05; however, the meta-combined regression coefficient was 0.024, which was not above the 0.1 threshold.

**Table 2:** Results of the epigenome-wide association studies (EWAS). (A) CpG sites associated with birth urine MEOHP and MnBP concentration variables with gene and CpG island annotation. (B) Sex-specific results of CpG sites associated with birth urine MnBP concentration.

CpG ID	Chr	Position	Coefficient (US)	*p*-value (US)	Coefficient (EW)	*p*-value (EW)	Coefficient (DK)	*p*-value (DK)	Coefficient (meta)	*p*-value (meta)	B-H adjusted	Gene annotation	CpG island annotation
(A) Urine MEOHP at birth													
cg12469381	7	29,519,297	− 0.076	1.9E−03	− 0.6	4.4E−06	0.078	5.4E−01	− 0.36	5.0E−08	4.0E−02	CHN2;CHN2	–
Urine MnBP at birth													
cg11229715	2	225,441,832	− 0.009	5.6E−01	− 0.44	3.4E−06	− 0.29	1.2E−05	− 0.31	2.5E−08	2.0E−02	CUL3	–

CpG ID	Chr	Position	logFC	AveExpr	*t*	*p*-value	B-H adjusted *p*-value	Gene annotation	CpG island annotation				
(B) Urine MnBP at birth, females													
cg05353481	7	143,806,743	0.0339	0.8739	7.3136	6.5E−09	5.2E−03	OR2A2	–				
cg22290225	15	66,319,931	0.0141	0.5484	6.7664	3.8E−08	1.5E−02	MEGF11	?				

Chr: chromosome; B-H: Benjamini-Hochberg; –: No CpG island annotation matched.

?: unknown; –: No CpG island annotation matched.

The sex-specific EWAS analysis revealed two significant CpGs associated with MnBP concentration in females in the birth group, cg05353481 and cg22290225, with an FDR-adjusted significance level of 0.05 (Table 2).

### Differentially methylated regions (DMRs) for all samples

Regarding MEOHP, MEHHP, MnBP, and DEHP, 5, 5, 6, and 6 DMRs were identified, respectively, with 3, 5, 4, and 5 unique overlapping genes, respectively, for phthalate concentrations in the maternal group; similarly, for phthalate concentrations in the birth group, 13, 11, 5, and 13 DMRs were identified, respectively, with 11, 10, 2, and 11 unique overlapping genes, respectively. The full list of identified DMRs is shown in Table 3. Sex-specific DMR analysis results are presented in Supplementary Table 1.

**Table 3:** Differentially methylated regions (DMRs) associated with phthalate concentration variables.

Chr	Start	End	N	Estimate	SE	*p*-value	Adjusted *p*-value	Overlapping gene
Maternal urine MEOHP, late pregnancy								
Chr16	5,666,398	5,666,499	2	− 0.1247	0.0272	4.7E-06	9.5E-03	*RP11-420N3.2*
Chr12	94,579,928	94,580,329	2	0.0799	0.0181	1.1E-05	2.2E-02	*PLXNC1*
Chr20	36,259,574	36,259,855	3	0.0922	0.0213	1.5E-05	3.1E-02	
Chr20	4,942,894	4,943,173	2	0.1236	0.0292	2.4E-05	4.9E-02	*SLC23A2*
Chr9	131,682,859	131,683,132	3	− 0.0650	0.0154	2.4E-05	4.9E-02	
Urine MEOHP at birth								
Chr16	67,999,044	67,999,055	2	− 0.2301	0.0479	1.6E-06	5.9E-03	*SLC12A4*
Chr11	65,357,780	65,358,078	2	− 0.2406	0.0503	1.7E-06	6.6E-03	*EHBP1L1*
Chr14	36,946,996	36,947,368	2	0.2105	0.0444	2.2E-06	8.2E-03	*RP11-896J10.3;SFTA3*
Chr17	7,493,572	7,494,095	4	− 0.1844	0.0401	4.3E-06	1.6E-02	*MPDU1*
Chr19	39,806,522	39,807,167	3	− 0.1748	0.0381	4.4E-06	1.7E-02	*CTC-246B18.8*
Chr1	162,530,191	162,530,364	2	− 0.2238	0.0491	5.3E-06	2.0E-02	*TBC1D7*
Chr6	13,295,922	13,295,976	3	− 0.2343	0.0515	5.4E-06	2.0E-02	*RP11-359K18.3*
Chr3	157,807,441	157,807,694	2	0.2384	0.0529	6.7E-06	2.5E-02	
Chr19	44,952,808	44,953,124	2	− 0.2087	0.0464	6.8E-06	2.6E-02	
Chr14	70,883,847	70,884,510	8	− 0.1323	0.0296	8.1E-06	3.1E-02	
Chr10	1,438,165	1,438,548	2	− 0.2264	0.0508	8.2E-06	3.1E-02	*ADARB2*
Chr15	40,630,371	40,630,791	2	− 0.1821	0.0414	1.1E-05	4.2E-02	*C15orf52*
Chr6	31,760,426	31,760,593	2	− 0.2210	0.0504	1.2E-05	4.4E-02	*VARS*
Maternal urine MEHHP, late pregnancy								
Chr3	69,249,276	69,249,718	2	0.0547	0.0120	4.8E-06	9.5E-03	*FRMD4B*
Chr8	6,481,582	6,481,608	2	− 0.1298	0.0289	7.1E-06	1.4E-02	*MCPH1;CTD-2541M15.1*
Chr16	5,666,398	5,666,499	2	− 0.1133	0.0258	1.1E-05	2.2E-02	*RP11-420N3.2*
Chr12	94,579,928	94,580,329	2	0.0751	0.0172	1.2E-05	2.4E-02	*PLXNC1*
Chr9	131,682,737	131,683,044	3	− 0.0630	0.0145	1.5E-05	2.9E-02	
Urine MEHHP at birth								
Chr16	67,999,044	67,999,055	2	− 0.2258	0.0469	1.5E-06	3.5E-03	*SLC12A4*
Chr19	39,806,522	39,807,167	3	− 0.1744	0.0369	2.3E-06	5.5E-03	*CTC-246B18.8*
Chr11	65,357,780	65,358,078	2	− 0.2309	0.0493	2.8E-06	6.5E-03	*EHBP1L1*
Chr13	39,358,777	39,358,795	2	− 0.2349	0.0512	4.4E-06	1.0E-02	*FREM2*
Chr14	36,946,996	36,947,368	2	0.1932	0.0436	9.6E-06	2.2E-02	*RP11-896J10.3;SFTA3*
Chr14	70,883,847	70,884,204	5	− 0.1434	0.0324	9.6E-06	2.2E-02	
Chr19	44,952,808	44,953,124	2	− 0.1982	0.0449	1.0E-05	2.3E-02	
Chr1	200,841,769	200,841,805	2	− 0.2098	0.0478	1.2E-05	2.7E-02	
Chr9	136,200,708	136,201,061	2	− 0.1863	0.0432	1.6E-05	3.8E-02	*SURF6*
Chr7	158,613,567	158,614,019	2	− 0.2341	0.0544	1.7E-05	4.0E-02	*ESYT2*
Chr12	4,918,169	4,919,230	10	0.1982	0.0464	1.9E-05	4.5E-02	*GALNT8;KCNA6*
Maternal urine MnBP, late pregnancy								
Chr9	131,682,737	131,683,132	4	− 0.0546	0.0106	2.4E-07	1.2E-03	
Chr12	133,406,047	133,406,309	2	− 0.0458	0.0096	1.8E-06	9.0E-03	*CHFR*
Chr15	34,394,035	34,394,207	3	− 0.0866	0.0188	4.3E-06	1.6E-02	*EMC7*
Chr16	30,816,719	30,816,924	2	− 0.0765	0.0169	5.9E-06	9.2E-02	
Chr6	170,038,695	170,038,733	2	0.0825	0.0184	7.5E-06	3.7E-02	*WDR27*
Chr10	7,708,869	7,708,934	2	− 0.0822	0.0186	9.5E-06	4.7E-02	*ITIH5*
Urine MnBP at birth								
Chr4	1,363,008	1,363,182	2	− 0.1577	0.0311	3.9E-07	2.1E-03	*UVSSA*
Chr19	39,806,522	39,807,167	3	− 0.1335	0.0284	2.6E-06	1.4E-02	*CTC-246B18.8*
Chr6	32,406,271	32,406,521	2	− 0.1922	0.0412	3.1E-06	1.7E-02	
Chr10	132,887,109	132,887,552	2	− 0.1635	0.0356	4.5E-06	2.4E-02	
Chr15	55,489,541	55,489,544	2	− 0.1905	0.0425	7.3E-06	3.9E-02	
Maternal urine DEHP, late pregnancy								
Chr16	5,666,398	5,666,499	2	− 0.1219	0.0268	5.6E-06	1.1E-02	*RP11-420N3.2*
Chr12	94,579,928	94,580,329	2	0.0782	0.0179	1.2E-05	2.5E-02	*PLXNC1*
Chr8	6,481,582	6,481,608	2	− 0.1316	0.0302	1.3E-05	2.7E-02	*MCPH1; CTD-2541M15.1*
Chr3	69,249,276	69,249,718	2	0.0542	0.0125	1.4E-05	2.7E-02	*FRMD4B*
Chr9	131,682,737	131,683,044	3	− 0.0659	0.0151	1.4E-05	2.7E-02	
Chr20	36,259,574	36,259,855	3	0.0885	0.0210	2.5E-05	5.0E-02	
Urine DEHP at birth								
Chr16	67,999,044	67,999,055	2	− 0.2365	0.0482	9.3E-07	3.0E-03	*SLC12A4*
Chr11	65,357,780	65,358,078	2	− 0.2458	0.0507	1.2E-06	3.9E-03	*EHBP1L1*
Chr19	39,806,522	39,807,167	3	− 0.1808	0.0381	2.1E-06	6.9E-03	*CTC-246B18.8*
Chr14	36,946,996	36,947,368	2	0.2112	0.0447	2.3E-06	7.3E-03	*RP11-896J10.3; SFTA3*
Chr17	7,493,572	7,494,095	4	− 0.1824	0.0405	6.5E-06	2.1E-02	*MPDU1*
Chr14	70,883,847	70,884,510	8	− 0.1339	0.0297	6.6E-06	2.1E-02	
Chr19	44,952,808	44,953,124	2	− 0.2084	0.0464	7.1E-06	2.3E-02	
Chr6	13,295,922	13,295,976	3	− 0.2323	0.0519	7.5E-06	2.4E-02	*TBC1D7*
Chr6	31,760,426	31,760,593	2	− 0.2249	0.0505	8.5E-06	2.7E-02	*VARS*
Chr1	162,530,191	162,530,364	2	− 0.2192	0.0496	9.7E-06	3.1E-02	*RP11-359K18.3*
Chr10	1,438,165	1,438,548	2	− 0.2246	0.0509	1.0E-05	3.3E-02	*ADARB2*
Chr16	1,824,041	1,824,177	2	− 0.2453	0.0565	1.4E-05	4.5E-02	*EME2*
Chr1	200,841,769	200,841,805	2	− 0.2149	0.0496	1.5E-05	4.7E-02	

n: number of CpGs; SE: standard error.

### Gene set analysis (GSA)

GSA was performed for MEOHP and MnBP concentrations in the birth group using their single significant DMPs. Under α = 0.05, for MEOHP and MnBP concentrations, two and 134 GO terms were identified, respectively, and two KEGG pathways were identified for MnBP concentration. The top ten terms are listed in Table 4. Under the FDR-adjusted significance level, no significant terms were identified.

**Table 4:** Results of gene set enrichment analysis using significant CpGs associated with birth urine MEOHP and MnBP concentrations. The top ten gene ontology (GO) and Kyoto Encyclopedia of Genes and Genomes (KEGG) terms under α = 0.05 are shown.

	Ontology	Term	N	DE	P.DE
Urine MEOHP at birth					
GO:0,051,056	BP	regulation of small GTPase-mediated signal transduction	316	1	4.4E-02
GO:0,043,547	BP	positive regulation of GTPase activity	376	1	4.4E-02
Urine MnBP at birth					
GO:0,045,842	BP	positive regulation of mitotic metaphase/anaphase transition	9	1	6.5E-03
GO:1,901,970	BP	positive regulation of mitotic sister chromatid separation	9	1	6.5E-03
GO:1,902,101	BP	positive regulation of metaphase/anaphase transition of cell cycle	10	1	7.4E-03
GO:0,040,016	BP	embryonic cleavage	7	1	7.4E-03
GO:0,001,829	BP	trophectodermal cell differentiation	15	1	8.4E-03
GO:1,905,820	BP	positive regulation of chromosome separation	11	1	8.9E-03
GO:0,071,218	BP	cellular response to misfolded protein	21	1	1.1E-02
GO:0,062,033	BP	positive regulation of mitotic sister chromatid segregation	17	1	1.2E-02
GO:0,051,788	BP	response to misfolded protein	23	1	1.3E-02
GO:0,007,080	BP	mitotic metaphase plate congression	44	1	1.3E-02

	Description	N	DE	P.DE	
path:hsa04340	Hedgehog signaling pathway	56	1	6.6E-02	
path:hsa04120	Ubiquitin-mediated proteolysis	134	1	9.6E-02	

BP: Biological Pathway; N: number of CpGs; DE: differentially methylated CpGs; P.DE: *p*-value.

Previously identified DMRs were also used to perform gene set analyses. No terms were identified under the 0.05 FDR-adjusted significance level. Under α = 0.05, for MEOHP, MEHHP, MnBP, and DEHP concentrations in the maternal group, 22, 29, 5, and 29 GO terms were significant, respectively, and one KEGG pathway was identified for MEOHP concentration. Also under α = 0.05, for MEOHP, MEHHP, MnBP, and DEHP concentration-associated terms in the birth group, 59, 41, 40, and 63 GO terms and 3, 0, 25, and 4 KEGG pathways, respectively, were identified (Supplementary Tables 2–5). The GO and KEGG terms associated with sex-specific DMRs are shown in Supplementary Tables 6–9 and 10–13 for females and males, respectively.

## Discussion

In this study, we found evidence of the relationship between DNA methylation and prenatal phthalate exposure, during late pregnancy. Additionally, two CpG sites in female infants were significantly associated with urine phthalate concentrations at birth. We also identified a significant association between DMRs and maternal and neonatal urinary phthalate concentrations during late pregnancy with each phthalate type (MEHHP, MEOHP, DEHP, and MnBP). We found GO and KEGG pathways under α = 0.05, using the identified DMPs and DMRs associated with multiple genes enriched for pathways related to embryonic development and tumorigenesis, cell cycle progression, and signal transduction. Under the 0.05 FDR-adjusted significance level, no significant terms were identified. Individual *p*-values for each term are not independent, and if adjusted, the *p*-values can become smaller. Results can also be affected by the small sample sizes. Overall, our results suggest a sex-specific association between prenatal exposure to phthalates and DNA methylation at specific CpG sites, indicating that Korean infants may be differentially susceptible to phthalate-induced epigenetic alterations.

In the urine samples obtained after birth, MEOHP concentrations were associated with CpG sites (cg12469381) located near the chimerin 2 (*CHN2*) gene, which encodes GTPase-activating protein, found mainly in the pancreas and brain^25^. Another differentially methylated CpG site was identified at position 225,441,832 on chromosome 2, corresponding to cullin3 (*CUL3*), suggesting that *CUL3* (cg11229715) also has a significant association with cord blood MnBP concentration. To the best of our knowledge, none of the genes associated with pregnancy phthalate concentrations covered in this study have been previously described in the context of epigenetic modifications or functions. However, based on the MRC-IEU EWAS catalog^26^, the CpG site cg12469381 extends beyond birth into late adolescence, impacting immune-neurodevelopmental functions, as shown in two population-based prospective birth cohorts^27^. Moreover, CUL3 is associated with pseudohypoaldosteronism and neurodevelopmental disorders, with or without autism or seizures^28^. Our results indicate that is a phthalate exposure-specific relationship with DNA methylation at specific CpG sites during pregnancy, but further research is needed to determine how prenatal exposure to phthalates impacts neurodevelopment and function.

In this study, the olfactory receptor family 2 subfamily A member 2 (*OR2A2*) gene for CpG sites (cg05353481) was significantly associated with birth urinary MnBP concentration, based on EWAS analysis for females. According to the EWAS catalog^26^, this gene is associated with age, maternal BMI, and nitrogen dioxide exposure^27,29,30^. The GO annotations associated with this gene include G protein-coupled receptor activity and taste receptor activity. Additionally, we found that in female infants another gene, cg22290225, annotated with Multiple EGF Like Domains 11 (*MEGF11*), was significantly associated with urine MnBP concentration. *MEGF11* was manifested in the embryonic retina but was detected in horizontal cells and starburst amacrine cells in the first postnatal week and persisted into adulthood^31^. To date, none of the other gene studies have reported differential DNA methylation associated with urinary MnBP at birth. Sex-specific effects of some phthalates on methylation and expression of genes in cord blood have previously been reported^16,20^. However, the mechanisms involved in these sex-specific differences are still unclear. One plausible explanation is that the sex chromosome may contribute to sex-specific differences in metabolism. Previous studies have shown that the active X chromosome has more methylated CpGs in the body of genes^32,33^. Moreover, exposure to phthalates may negatively affect fetal development parameters, such as reduction in gestational age and preterm birth, and these effects can be sex-specific^4,13,34^. Considering this, it is important to note even small magnitudes of effects in these studies when they are sex-specific; our results provide further evidence for the association between prenatal phthalate exposure and epigenetic changes.

We identified more specific DMRs associated with phthalate metabolites in both maternal and infants, respectively, and found no overlap between them. Among the phthalate overlapping genes for MEOHP, MEHHP, and DEHP, in marternal, *PLXNC1* belong to the family of trans-membrane receptors. It participates in the development of the nervous and immune systems through neuronal polarity, axon guidance, cellular motility, migration, and the immune response^35,36^. *CHFR* was significantly associated with maternal MnBP concentration in late pregnancy. As a tumorigenic factor, this gene promotes cell cycle progression and may induce ubiquitination by promoting polyubiquitination and degradation of *HDAC1*^37,38^. In addition, we examined the associations between DMR and each metabolite at birth. The EH domain-binding protein 1-like 1 (*EHBP1L1*) gene has a significantly hypomethylated DMR associated with MEOHP, MEHHP and DEHP concentrations at birth in infants. This gene is involved in the differentiation process of spermatogenesis and testicular activity^39^. A hypomethylated region of the microcephalin 1 (*MCPH1*) gene is involved in mitotic delay and irregular spindle orientation, resulting in defects in *MCPH1* and an early change in cell division from symmetric to asymmetric, owing to the depletion of the progenitor pool and microencephaly^40,41^. Interestingly, urinary MnBP concentration was positively associated with two DMRs at birth. *UVSSA* encodes protein-coding genes and plays a role in the genome integrity homeostasis network. It is also involved in RNA polymerase II processing and repair factor recruitment. Defects in *UVSSA* affect the TC-NER pathway in RNAPII processing in various types of DNA damage, possibly contributing to the development of a neurodegenerative phenotype common in disorders associated with genome instability^42^. Overall, the DMRs found in our study in genes associated with phthalate exposure either in the urine of mothers during late pregnancy or infants are closely related to neurological functions, indicating the importance of the effects of phthalate exposure on genes associated with embryonic development.

In our study, using gene set enrichment analysis, we showed that urinary MEOHP and MnBP concentrations at birth were associated with GO and KEGG pathways during late pregnancy. Several of these functional categories, such as regulation of signal transduction mediated by small GTPases and positive regulation of GTPase activity, were involved in biological processes associated with urinary MEOHP concentration at birth. In another study, a similar function was demonstrated for different phthalate metabolites, such as butyl benzyl phthalate (BBzP), which is associated with signal transduction mediated by small GTPases only in female infants^20^. In the present study, embryonic cleavage, positive regulation of mitotic metaphase, and mitotic sister chromatid separation were significantly associated with urinary MnBP concentration at birth. A previous study suggested that phthalates may be associated with sperm methylation in or near genes important for early embryogenesis^43^. This would explain a biological pathway linking the observed inverse relationship between the antiandrogenic effects of phthalates and blastocyst quality. Additionally, we observed phthalate-induced methylation in sites associated with the Hedgehog signaling pathway or ubiquitin-mediated proteolysis. This signaling pathway has been demonstrated in vitro and in vivo system^43–46^. One study has reported that the effect of preconception exposure to DEHP on genome-wide DNA methylation and gene expression profiles in mice, was related to the Hedgehog signaling pathway^43^. In addition, other studies have found that in utero exposure to dibutyl phthalate (DBP) led to abnormal proliferation of testicular Sertoli cells in prepubertal mice by modulating the ubiquitination of the key proliferation-related protein IRAK1 via the downregulation of *Peli2*^46^. Several studies have shown that phthalate exposure was associated with numerous enriched inflammatory pathways (e.g., NF-κB, MAPK, TNF-β)^19,47^. In our study, these pathways were not significantly associated with phthalate exposure in late pregnancy or phthalate levels in cord blood. However, methylation is positively associated with the expression of some genes, and it is necessary to investigate whether the methylation process is involved in the regulation of these pathways. Understanding the molecular differences affecting these CpGs sites may lead to a better understanding of possible abnormalities in fetal development caused by prenatal phthalate exposure.

Our study was specifically designed to investigate the relationship between prenatal phthalate exposure and DNA methylation in cord blood. However, this study has some limitations. First, the sample size of cord blood was relatively small, which limits the statistical power of the analysis or the availability of methods. The meta-analysis revealed two CpG sites and two other CpG sites in females that were significantly associated with cord blood concentration. However, we could not find any correlation between maternal phthalate concentrations and CpG sites. A larger sample size would allow for greater statistical power and could help identify relevant DMPs. Further studies with a larger population are needed to confirm the findings of our study. Second, the participants in this study were in the late stages of pregnancy. Phthalates have an extremely short half-life and are rapidly excreted from the body^48^; therefore, a single estimate is not representative of the entire pregnancy. However, most significant DMRs are generally observed in late pregnancy^14,20^. Moreover, we defined the possible biological processes affected by prenatal phthalate exposure based on GO and KEGG pathways, which may be useful for further research. Lastly, a previous study suggested that some probes in the Illumina MethylationEPIC BeadChip can bind with polymorphic sites or hybridize with non-specific sites. While these types of probes can still represent methylation status at the appropriate sites, the results could be inaccurate if the probe itself is methylated^49^. To better explore the epigenome associated with phthalates, our study used the EPIC BeadChip (loci:850,000) instead of the 450 k BeadChips (loci:450,000). Although the EPIC BeadChip approach has become the standard in EWAS studies, the reported percentages of differentially methylated CpGs and enrichment-associated genes may be biased because of the lower coverage of epigenome sequencing. Therefore, these probes need to be interpreted more carefully, and additional reporting or biological validation in different cohorts may be required.

In conclusion, there is a significant association between prenatal phthalate exposure and DNA methylation at multiple CpG sites. Additionally, urinary MnBP levels at birth were significantly associated with CpG sites in female infants. Our study has uncovered several functional mechanisms and associated genes, providing insight into the effects of phthalate exposure on fetal development and neurodevelopmental disorders. Further research is needed to investigate the mechanisms behind the adverse effects of prenatal phthalate exposure on human well-being.

## Methods

### Study population and phthalate measurement

From 2006 to 2010, pregnant women within 20 weeks of pregnancy and over 18 years of age were recruited from the Mother and Child’s Environmental Health (MOCEH) multicenter prospective cohort study. The health centers were located in Ulsan, Ewha, and Dankook. Patients who were planning to move out of the locations within a year or were diagnosed with cognitive impairment or mental disease were excluded from the study. Details on the recruitment process for this cohort can be found in a previous study^50^. All participants provided written informed consent during their first prenatal visits, and during routine face-to-face visits. Self-administered questionnaires were completed to collect sociodemographic information. This study was approved by the Institutional Review Board of Kangwon National University Hospital (2017-11-006). This study was conformed to the tenets of the Declaration of Helsinki.

Urine samples were collected from women during late pregnancy (28–42 weeks of gestation) and from newborns, immediately after birth, and stored at -80 °C for posterior analysis. In terms of phthalate selection, the two metabolites of di(2-ethylhexyl) phthalate (DEHP), mono-(2-ethyl-5-oxohexyl) phthalate (MEOHP) and mono (2-ethyl-5-hydroxyhexyl) phthalate (MEHHP), and one metabolite of di-n-butyl phthalate (DnBP), Mono-n-butyl phthalate (MnBP), were selected. DEHP and DnBP are two common phthalates to which humans are easily exposed through personal care products and plastics^51^.

Phthalate concentrations in the urine samples were measured according to the MOCEH study protocol^50^. Samples were analyzed using high-performance liquid chromatography-tandem mass spectrometry (Agilent Technologies, Inc., California, USA). The limits of detection (LODs) for MEOHP, MEHHP, and MnBP were 0.56 $\upmu$g/L, 0.49 $\upmu$g/L, and 0.44 $\upmu$g/L, respectively. Measurements below the detection limit were recorded as half the LOD. The DEHP concentration was calculated by summing MEOHP and MEHHP concentrations^51^.

### DNA methylation data preprocessing

DNA methylation level was measured from the cord blood samples in MOCEH cohort, following the instructions of the Illumina HumanMethylationEPIC BeadChip kit (Illumina, California) and methods from the Korean Exposome Study^52,53^. The measured data were preprocessed following the recommended quality control (QC) workflow of the ‘ewastools’ package in R^54,55^. Probes with detection *p*-values above the significance threshold of 0.01 were set to missing, and dye-bias was corrected with the RELIC method^56^ using the Theil–Sen estimator. Then, beta values were calculated as the ratio of methylated to total (methylated and unmethylated combined) signal intensities. Leukocyte composition was estimated using the beta values with the Houseman method^57^. Based on the reference panel given by Salas et al., cell deconvolution was achieved using the CpGs optimized with Identifying Optimal Libraries (IDOL) and the “estimateCellCounts2” function with the “CordBlood” option in the “FlowSorted.Blood.EPIC” package in R, which is an expansion of the “minfi” package^58–60^.

### Quality control (QC)

The “ewastools” package was used to perform QC on the subjects and CpGs. For the subject-level QC, following the methods of Park et al^53^., subjects were excluded according to the following criteria: (1) one of the twins; (2) recommended thresholds of the 17 metrics from the Illumina BeadArray Controls Reporter Software Guide^61^; (3) inconsistency between observed sex and sex inferred from normalized X/Y probe intensities; (4) outliers or duplicates for the SNP probe intensities; (5) outliers for the principal components of the autosomal beta values; or (6) outliers for the leukocyte composition. For step (4), a detailed quality check procedure^54^ was performed according to a previously described process. The beta values for SNP probes were trained with a mixture model with three beta distributions for subjects with genotypes AA/Aa/aa and a uniform distribution for outlying subjects. The average log odd of being outliers was calculated for each subject across all SNPs, and if this value was above -4, the corresponding subject was marked as an outlier. Moreover, the posterior probabilities for each genotype were used to calculate the proportions of SNPs for which both had the same genotype for each pair of subjects, excluding those with proportions exceeding 90%. For steps (5) and (6), a multidimensional scaling (MDS) plot was generated, and outliers were manually detected. Likewise, leukocyte compositions were plotted separately for each cell type, and manually-detected outliers were filtered out. The number of subjects excluded by QC procedures is shown in Supplementary Table 1 of a previous study^55^. For CpG-level QC, probes with the following criteria were excluded: (1) sites with > 3% missing data or SNPs; (2) probes from the X or Y chromosome; or (3) previously revealed cross-reactive probes^62^. Finally, 364 subjects and 793,925 sites were included in the analysis.

### Covariate imputation

After excluding one randomly selected twin, 383 subjects were used for the imputation of missing values of the following seven covariates using the “missForest” package in R^55,63^: infant sex, maternal pre-pregnancy body mass index (BMI), smoking history, depending on whether the participants had smoked at least 400 cigarettes, and early and late pregnancy cotinine and creatinine concentrations in maternal blood. For the dataset used for the analysis, please refer to Supplementary Table 2 of a previous published study^55^. Using imputed values for smoking history and late pregnancy cotinine concentrations, participants were classified as “current smokers” if their late pregnancy cotinine concentrations (measured or imputed) were not less than 200 µg/g or if they answered “yes” to a questionnaire on whether they continued to smoke. For the actual statistical analysis, subjects with missing sex information were excluded because (1) the QC steps require sex to be reported and (2) it would be more robust to use only observed values if stratification by sex was performed.

### Epigenome-wide association study (EWAS)

An EWAS was performed on MEOHP, MEHHP, MnBP, and DEHP concentrations in the maternal and birth groups. EWAS was initially performed separately for the three centers, and the results were later combined with the meta-analysis in order to minimize the heterogeneity among centers. For each center, linear regression was performed on each of the CpG sites using the “limma” package in R to test the association between the beta value of methylation level and log2-transformed phthalate concentration in urine^55,64^. The covariates included in the model were the study enrollment year, infant sex, maternal pre-pregnancy BMI, current smoking status, average monthly household income, and estimated leukocyte composition. Batch information was included as a random effect to adjust for batch effects. The *p*-values from each center were combined using Liptak’s method^65^, and regression coefficients were combined using weights inversely proportional to the standard errors.

Also, using the sex of birth subjects, sex-stratified EWAS was performed for samples in the birth group. Due to the limited sample size, samples from the three centers were combined and analyzed together (mega-analysis). The sex-specific sample sizes for each center are listed in Table 1.

### Differentially methylated region (DMR) analysis

The DMRs associated with phthalate concentration in urine were separately identified from the three centers and combined with meta-analysis using the “dmrff” package in R and Rex (version 3.6.0)^66,67^. The methylation levels of each CpG site were first transformed using inverse normal transformation before the analysis to obtain results robust to outliers and the normal distribution assumption. Regions with a maximum distance of 500 bp between consecutive features and at least two significant probes at a significance level of 0.05 were identified. The identified DMRs were evaluated using the 0.05 Bonferroni adjusted significance level. ENSEMBL_MART_ENSEMBL BioMart database and the hsapiens_gene_ensembl database in the Ensembl genome browser (version: GRCh37) were used for annotation. Mega-analysis for sex-specific DMR identification in the birth group was performed using the “dmrff” R package. Annotation was performed using the same procedure as described above.

### Gene set analysis (GSA)

Gene ontology (GO) terms and Kyoto Encyclopedia of Genes and Genomes (KEGG) pathways were identified using the significant CpGs and DMRs. Terms with at least five CpG sites were used to create a gene set, and each gene set was tested using the 0.05 FDR-adjusted significance level. GSA was performed using the “missMethyl” package in R^55,66^. The sex-specific gene set analysis in the birth group was performed using the same procedure.

## Supplementary Information

Supplementary Information.

## Data Availability

Data from this study are available from the corresponding author upon reasonable request.
