# Supplementary material for: Prenatal phthalate exposure and cord blood DNA methylation
Source: Sci Rep. 2023 Apr 29;13:7046. doi: 10.1038/s41598-023-33002-8 (PMC10148847; doi:10.1038/s41598-023-33002-8)
Supplement: Supplementary file 1 — Supplementary Information. [file 41598_2023_33002_MOESM1_ESM.docx]

**Supplementary Table 1.** Sex-specific DMRs associated with MEOHP, MEHHP, MnBP, and DEHP concentration in maternal urine samples during late pregnancy and birth urine samples (A: female, B: male).

(A)

| **Maternal urine MEOHP, late pregnancy** | | | | | | | | |
| --- | --- | --- | --- | --- | --- | --- | --- | --- |
| **chr** | **start** | **end** | **n** | **estimate** | **SE** | ***p*-value** | **adjusted *p*-value** | **Overlapping gene** |
| chr6 | 31803209 | 31805329 | 13 | -0.1560 | 0.0289 | 6.82E-08 | 0.0004 | *C6orf48; SNORD52* |
| chr14 | 77228193 | 77228497 | 2 | -0.1609 | 0.0341 | 2.40E-06 | 0.0156 |  |
| chr13 | 44453183 | 44453208 | 2 | -0.1685 | 0.0363 | 3.34E-06 | 0.0218 | *CCDC122* |
| chr16 | 84004435 | 84005252 | 5 | 0.1596 | 0.0344 | 3.52E-06 | 0.0229 | *NECAB2* |
| chr11 | 27372753 | 27373021 | 2 | 0.1702 | 0.0371 | 4.46E-06 | 0.0290 | *CCDC34* |
| chr1 | 54665230 | 54665764 | 8 | -0.1414 | 0.0311 | 5.56E-06 | 0.0362 | *RP11-446E24.4; CYB5RL; MRPL37* |
| chr14 | 23540729 | 23540794 | 3 | -0.1347 | 0.0296 | 5.56E-06 | 0.0362 | *ACIN1* |
| chr2 | 20851152 | 20851304 | 2 | -0.1783 | 0.0398 | 7.61E-06 | 0.0495 |  |
| **Birth urine MEOHP** | | | | | | | | |
| **chr** | **start** | **end** | **n** | **estimate** | **SE** | ***p*-value** | **adjusted *p*-value** | **Overlapping gene** |
| chr20 | 57192078 | 57192288 | 2 | -0.3403 | 0.0735 | 3.60E-06 | 0.0039 | *APCDD1L-AS1* |
| chr6 | 170500610 | 170500638 | 2 | -0.3569 | 0.0793 | 6.73E-06 | 0.0074 |  |
| chr6 | 74009455 | 74009865 | 2 | -0.3039 | 0.0684 | 8.75E-06 | 0.0096 | *KHDC1; RP11-398K22.12* |
| chr11 | 73965049 | 73965265 | 2 | -0.3016 | 0.0680 | 9.11E-06 | 0.0100 | *PPME1; P4HA3* |
| chr6 | 168378810 | 168379113 | 4 | -0.2499 | 0.0573 | 1.28E-05 | 0.0140 | *RP3-470B24.5* |
| chr8 | 143389587 | 143389706 | 2 | -0.3097 | 0.0726 | 1.97E-05 | 0.0216 | *TSNARE1* |
| chr4 | 182186286 | 182186820 | 4 | -0.3822 | 0.0899 | 2.12E-05 | 0.0233 |  |
| chr7 | 51384676 | 51384931 | 2 | -0.3180 | 0.0750 | 2.25E-05 | 0.0247 |  |
| chr1 | 917571 | 917661 | 3 | 0.3675 | 0.0869 | 2.33E-05 | 0.0255 |  |
| chr20 | 44541745 | 44541804 | 2 | -0.3139 | 0.0743 | 2.41E-05 | 0.0264 |  |
| chr3 | 50232343 | 50232459 | 2 | 0.3532 | 0.0838 | 2.48E-05 | 0.0272 | *GNAT1* |
| chr4 | 182230157 | 182230345 | 2 | 0.3745 | 0.0897 | 2.95E-05 | 0.0323 |  |
| chr7 | 158266097 | 158266198 | 3 | -0.3802 | 0.0913 | 3.10E-05 | 0.0340 | *PTPRN2* |
| chr3 | 9343708 | 9344105 | 3 | -0.2711 | 0.0652 | 3.23E-05 | 0.0353 | *SRGAP3; RP11-380O24.1* |
| chr6 | 31829205 | 31829292 | 2 | -0.2957 | 0.0718 | 3.85E-05 | 0.0422 | *NEU1* |
| chr15 | 26328049 | 26328092 | 2 | -0.3781 | 0.0924 | 4.28E-05 | 0.0470 |  |
| chr6 | 158093525 | 158093883 | 2 | -0.2585 | 0.0633 | 4.46E-05 | 0.0488 | *ZDHHC14* |
| **Maternal urine MEHHP, late pregnancy** | | | | | | | | |
| **chr** | **start** | **end** | **n** | **estimate** | **SE** | ***p*-value** | **adjusted *p*-value** | **Overlapping gene** |
| chr6 | 31803182 | 31805329 | 16 | -0.1239 | 0.0245 | 4.17E-07 | 0.0033 | *C6orf48; SNORD52* |
| chr14 | 77228193 | 77228497 | 2 | -0.1635 | 0.0324 | 4.39E-07 | 0.0035 |  |
| chr11 | 27372753 | 27373021 | 2 | 0.1660 | 0.0353 | 2.60E-06 | 0.0207 | *CCDC34* |
| **Birth urine MEHHP** | | | | | | | | |
| **chr** | **start** | **end** | **n** | **estimate** | **SE** | ***p*-value** | **adjusted *p*-value** | **Overlapping gene** |
| chr7 | 158266097 | 158266198 | 3 | -0.4756 | 0.0928 | 2.99E-07 | 0.0003 | *PTPRN2* |
| chr10 | 29228804 | 29228916 | 3 | -0.4104 | 0.0928 | 9.87E-06 | 0.0091 |  |
| chr6 | 170500610 | 170500638 | 2 | -0.3675 | 0.0860 | 1.93E-05 | 0.0177 |  |
| chr1 | 917655 | 917661 | 2 | 0.4337 | 0.1020 | 2.13E-05 | 0.0196 |  |
| chr6 | 158093525 | 158093883 | 2 | -0.2839 | 0.0674 | 2.55E-05 | 0.0234 | *ZDHHC14* |
| chr16 | 14029456 | 14029472 | 2 | -0.3250 | 0.0789 | 3.81E-05 | 0.0349 | *ERCC4; CTD-2135D7.2* |
| chr12 | 109613665 | 109614094 | 2 | -0.3074 | 0.0748 | 3.95E-05 | 0.0363 | *ACACB* |
| chr13 | 112729515 | 112729991 | 2 | 0.3820 | 0.0937 | 4.57E-05 | 0.0420 |  |
| chr21 | 46368052 | 46368313 | 4 | -0.2680 | 0.0663 | 5.30E-05 | 0.0486 | *FAM207A* |
| **Maternal urine MnBP, late pregnancy** | | | | | | | | |
| **chr** | **start** | **end** | **n** | **estimate** | **SE** | ***p*-value** | **adjusted *p*-value** | **Overlapping gene** |
| chr17 | 79614917 | 79615824 | 6 | -0.1144 | 0.0207 | 3.07E-08 | 0.0005 | *NPLOC4; TSPAN10* |
| chr10 | 102415086 | 102415320 | 4 | -0.1223 | 0.0236 | 2.19E-07 | 0.0033 |  |
| chr21 | 38580129 | 38580484 | 2 | -0.0984 | 0.0195 | 4.72E-07 | 0.0071 |  |
| chr5 | 88180332 | 88180499 | 2 | -0.1180 | 0.0244 | 1.26E-06 | 0.0189 | *MEF2C; MEF2C-AS1* |
| chr8 | 65499048 | 65499369 | 3 | -0.1251 | 0.0259 | 1.35E-06 | 0.0202 |  |
| chr17 | 2117809 | 2118147 | 2 | -0.0639 | 0.0132 | 1.38E-06 | 0.0207 | *SMG6* |
| chr17 | 58499065 | 58499300 | 2 | -0.0941 | 0.0196 | 1.62E-06 | 0.0243 | *USP32; C17orf64* |
| chr1 | 27683351 | 27684241 | 8 | -0.0797 | 0.0166 | 1.70E-06 | 0.0254 | *MAP3K6* |
| chr4 | 4866047 | 4866454 | 5 | -0.1269 | 0.0265 | 1.70E-06 | 0.0255 |  |
| chr3 | 93692773 | 93692925 | 2 | -0.1267 | 0.0268 | 2.26E-06 | 0.0339 | *PROS1* |
| chr8 | 13424419 | 13424483 | 2 | -0.1217 | 0.0258 | 2.30E-06 | 0.0344 | *RP11-145O15.3; C8orf48* |
| chr11 | 10325255 | 10325440 | 3 | -0.1090 | 0.0231 | 2.44E-06 | 0.0366 |  |
| chr18 | 52626476 | 52627127 | 9 | -0.0729 | 0.0156 | 2.92E-06 | 0.0438 | *CCDC68* |
| chr7 | 27237893 | 27238059 | 4 | -0.1194 | 0.0255 | 2.98E-06 | 0.0446 | *HOXA13* |
| chr20 | 60697463 | 60697728 | 4 | -0.0825 | 0.0177 | 3.10E-06 | 0.0465 | *LSM14B* |
| chr19 | 14260591 | 14260651 | 2 | -0.1319 | 0.0283 | 3.22E-06 | 0.0483 | *CTB-55O6.12* |
| chr17 | 79624018 | 79624126 | 2 | -0.1430 | 0.0307 | 3.27E-06 | 0.0490 | *PDE6G* |
| **Birth urine MnBP** | | | | | | | | |
| **chr** | **start** | **end** | **n** | **estimate** | **SE** | ***p*-value** | **adjusted *p*-value** | **Overlapping gene** |
| chr11 | 129907095 | 129907308 | 2 | -0.2900 | 0.0587 | 7.77E-07 | 0.0012 |  |
| chr8 | 35581557 | 35581595 | 2 | -0.2991 | 0.0633 | 2.29E-06 | 0.0037 | *UNC5D* |
| chr16 | 67997858 | 67998466 | 8 | -0.2422 | 0.0559 | 1.50E-05 | 0.0239 | *SLC12A4* |
| chr2 | 6910631 | 6911113 | 2 | 0.2440 | 0.0566 | 1.61E-05 | 0.0257 |  |
| chr3 | 120314602 | 120314841 | 2 | -0.2416 | 0.0566 | 1.96E-05 | 0.0314 |  |
| chr10 | 130064021 | 130064296 | 3 | -0.2997 | 0.0717 | 2.88E-05 | 0.0460 |  |
| **Maternal urine DEHP, late pregnancy** | | | | | | | | |
| **chr** | **start** | **end** | **n** | **estimate** | **SE** | ***p*-value** | **adjusted *p*-value** | **Overlapping gene** |
| chr6 | 31803209 | 31805329 | 13 | -0.1508 | 0.0286 | 1.38E-07 | 0.0010 | *C6orf48; SNORD52* |
| chr14 | 77228193 | 77228497 | 2 | -0.1650 | 0.0335 | 8.72E-07 | 0.0064 |  |
| chr11 | 27372753 | 27373021 | 2 | 0.1713 | 0.0365 | 2.70E-06 | 0.0198 | *CCDC34* |
| chr13 | 44453183 | 44453208 | 2 | -0.1658 | 0.0358 | 3.60E-06 | 0.0264 | *CCDC122* |
| chr1 | 54665230 | 54665764 | 8 | -0.1405 | 0.0307 | 4.75E-06 | 0.0348 | *RP11-446E24.4; CYB5RL; MRPL37* |
| chr14 | 23540729 | 23540794 | 3 | -0.1334 | 0.0293 | 5.15E-06 | 0.0378 | *ACIN1* |
| chr11 | 75526013 | 75526058 | 2 | -0.1930 | 0.0427 | 6.10E-06 | 0.0447 | *RP11-535A19.2* |
| **Birth urine DEHP** | | | | | | | | |
| **chr** | **start** | **end** | **n** | **estimate** | **SE** | ***p*-value** | **adjusted *p*-value** | **Overlapping gene** |
| chr6 | 170500610 | 170500638 | 2 | -0.3814 | 0.0803 | 2.01E-06 | 0.0021 |  |
| chr7 | 158266097 | 158266198 | 3 | -0.4240 | 0.0910 | 3.16E-06 | 0.0033 | *PTPRN2* |
| chr20 | 57192078 | 57192288 | 2 | -0.3414 | 0.0754 | 5.95E-06 | 0.0061 | *APCDD1L-AS1* |
| chr6 | 158093525 | 158093883 | 2 | -0.2800 | 0.0641 | 1.26E-05 | 0.0130 | *ZDHHC14* |
| chr6 | 74009455 | 74009865 | 2 | -0.3062 | 0.0702 | 1.28E-05 | 0.0132 | *KHDC1; RP11-398K22.12* |
| chr6 | 168378810 | 168379113 | 4 | -0.2549 | 0.0586 | 1.36E-05 | 0.0140 |  |
| chr11 | 73965049 | 73965265 | 2 | -0.2975 | 0.0698 | 2.04E-05 | 0.0210 | *PPME1; P4HA3* |
| chr21 | 46368052 | 46368313 | 4 | -0.2670 | 0.0631 | 2.34E-05 | 0.0241 | *FAM207A* |
| chr10 | 29228804 | 29228916 | 3 | -0.3747 | 0.0899 | 3.07E-05 | 0.0317 |  |
| chr3 | 9343708 | 9344105 | 3 | -0.2761 | 0.0667 | 3.45E-05 | 0.0356 | *SRGAP3; RP11-380O24.1* |
| chr3 | 32927676 | 32927740 | 2 | 0.2649 | 0.0640 | 3.48E-05 | 0.0360 | *TRIM71* |
| chr12 | 109613665 | 109614094 | 2 | -0.2956 | 0.0716 | 3.61E-05 | 0.0373 | *ACACB* |
| chr4 | 182186286 | 182186820 | 4 | -0.3797 | 0.0926 | 4.16E-05 | 0.0429 |  |
| chr20 | 44541745 | 44541804 | 2 | -0.3131 | 0.0765 | 4.26E-05 | 0.0440 |  |
| chr1 | 917571 | 917661 | 3 | 0.3658 | 0.0894 | 4.30E-05 | 0.0444 |  |

(B)

| **Maternal urine MEOHP, late pregnancy** | | | | | | | | |
| --- | --- | --- | --- | --- | --- | --- | --- | --- |
| **chr** | **start** | **end** | **n** | **estimate** | **SE** | ***p*-value** | **adjusted *p*-value** | **Overlapping gene** |
| chr3 | 182511312 | 182511317 | 2 | 0.1541 | 0.0330 | 2.95E-06 | 0.0038 | *ATP11B* |
| chr18 | 2554720 | 2554914 | 2 | -0.1595 | 0.0352 | 5.74E-06 | 0.0075 | *METTL4* |
| chr11 | 102359753 | 102359781 | 2 | -0.1417 | 0.0314 | 6.55E-06 | 0.0085 | *RP11-315O6.2* |
| chr10 | 106013813 | 106014311 | 7 | 0.0577 | 0.0131 | 1.01E-05 | 0.0131 | *GSTO1* |
| chr19 | 10213271 | 10213619 | 3 | 0.0652 | 0.0151 | 1.64E-05 | 0.0212 | *ANGPTL6* |
| chr8 | 10331676 | 10331891 | 3 | -0.1168 | 0.0272 | 1.71E-05 | 0.0222 |  |
| chr1 | 235106679 | 235107109 | 2 | 0.0523 | 0.0122 | 1.81E-05 | 0.0235 |  |
| chr12 | 53730380 | 53730421 | 3 | 0.1007 | 0.0241 | 2.99E-05 | 0.0388 | *SP7* |
| chr4 | 74847761 | 74848392 | 7 | 0.0840 | 0.0202 | 3.25E-05 | 0.0422 | *PF4* |
| chr2 | 179916147 | 179916184 | 2 | 0.1166 | 0.0283 | 3.67E-05 | 0.0477 |  |
| **Birth urine MEOHP** | | | | | | | | |
| **chr** | **start** | **end** | **n** | **estimate** | **SE** | ***p*-value** | **adjusted *p*-value** | **Overlapping gene** |
| chr17 | 80928410 | 80929145 | 5 | -0.2058 | 0.0382 | 7.13E-08 | 0.0001 | *B3GNTL1* |
| chr19 | 18700553 | 18700933 | 2 | -0.3142 | 0.0600 | 1.64E-07 | 0.0003 | *CRLF1; C19orf60* |
| chr21 | 42219202 | 42219211 | 2 | 0.2761 | 0.0539 | 3.01E-07 | 0.0005 |  |
| chr3 | 194868750 | 194868790 | 2 | -0.3141 | 0.0671 | 2.87E-06 | 0.0044 | *XXYLT1; XXYLT1-AS2* |
| chr22 | 47439366 | 47439477 | 2 | -0.2253 | 0.0509 | 9.46E-06 | 0.0146 | *TBC1D22A* |
| chr8 | 143867129 | 143867383 | 2 | -0.1961 | 0.0454 | 1.59E-05 | 0.0244 | *LY6D; RP11-706C16.8* |
| chr4 | 736765 | 737005 | 2 | -0.2459 | 0.0571 | 1.67E-05 | 0.0257 | *PCGF3* |
| chr7 | 157956042 | 157956050 | 2 | 0.2187 | 0.0517 | 2.33E-05 | 0.0358 | *PTPRN2* |
| **Maternal urine MEHHP, late pregnancy** | | | | | | | | |
| **chr** | **start** | **end** | **n** | **estimate** | **SE** | ***p*-value** | **adjusted *p*-value** | **Overlapping gene** |
| chr5 | 67730497 | 67730595 | 2 | 0.0470 | 0.0085 | 2.62E-08 | 0.0000 |  |
| chr3 | 182511312 | 182511317 | 2 | 0.1513 | 0.0313 | 1.33E-06 | 0.0018 | *ATP11B* |
| chr1 | 217672412 | 217672626 | 2 | -0.1110 | 0.0254 | 1.25E-05 | 0.0168 | *GPATCH2* |
| chr11 | 102359753 | 102359781 | 2 | -0.1289 | 0.0300 | 1.77E-05 | 0.0239 | *RP11-315O6.2* |
| chr7 | 3991031 | 3991307 | 2 | -0.1263 | 0.0294 | 1.78E-05 | 0.0239 | *SDK1* |
| chr1 | 235106679 | 235107109 | 2 | 0.0488 | 0.0116 | 2.56E-05 | 0.0344 |  |
| chr12 | 94676369 | 94676708 | 5 | 0.1016 | 0.0245 | 3.34E-05 | 0.0449 | *PLXNC1; RP11-1105G2.3* |
| **Birth urine MEHHP** | | | | | | | | |
| **chr** | **start** | **end** | **n** | **estimate** | **SE** | ***p*-value** | **adjusted *p*-value** | **Overlapping gene** |
| chr19 | 18700553 | 18700933 | 2 | -0.2877 | 0.0567 | 3.92E-07 | 0.0009 | *CRLF1; C19orf60* |
| chr3 | 194868750 | 194868790 | 2 | -0.3113 | 0.0616 | 4.39E-07 | 0.0010 | *XXYLT1; XXYLT1-AS2* |
| chr17 | 80928410 | 80929145 | 5 | -0.1821 | 0.0361 | 4.48E-07 | 0.0010 | *B3GNTL1* |
| chr6 | 31461728 | 31461850 | 2 | -0.2232 | 0.0471 | 2.14E-06 | 0.0050 |  |
| chr5 | 177870276 | 177870407 | 4 | -0.2090 | 0.0445 | 2.60E-06 | 0.0061 | *COL23A1* |
| chr10 | 50285414 | 50285427 | 2 | -0.2321 | 0.0499 | 3.34E-06 | 0.0078 | *VSTM4* |
| chr22 | 47439366 | 47439477 | 2 | -0.2154 | 0.0474 | 5.42E-06 | 0.0127 | *TBC1D22A* |
| chr20 | 36148604 | 36149188 | 27 | -0.1836 | 0.0406 | 5.97E-06 | 0.0140 | *BLCAP* |
| chr7 | 195656 | 195679 | 2 | -0.2341 | 0.0529 | 9.66E-06 | 0.0226 | *FAM20C* |
| chr8 | 41522721 | 41522809 | 2 | -0.2061 | 0.0473 | 1.34E-05 | 0.0313 | *ANK1; RP11-930P14.1* |
| chr10 | 135071851 | 135072039 | 2 | -0.1377 | 0.0322 | 1.88E-05 | 0.0439 |  |
| chr21 | 42219202 | 42219211 | 2 | 0.2243 | 0.0526 | 1.99E-05 | 0.0465 |  |
| chr16 | 3493641 | 3494094 | 7 | -0.2056 | 0.0482 | 2.01E-05 | 0.0471 | *NAA60* |
| **Maternal urine MnBP, late pregnancy** | | | | | | | | |
| **chr** | **start** | **end** | **n** | **estimate** | **SE** | ***p*-value** | **adjusted *p*-value** | **Overlapping gene** |
| chr11 | 77158423 | 77158483 | 2 | 0.0720 | 0.0163 | 1.02E-05 | 0.0095 | *PAK1* |
| chr12 | 131919527 | 131919579 | 2 | -0.1249 | 0.0285 | 1.19E-05 | 0.0112 |  |
| chr16 | 2985833 | 2986201 | 2 | 0.0800 | 0.0184 | 1.43E-05 | 0.0134 | *FLYWCH1* |
| chr3 | 193272778 | 193272998 | 4 | -0.1141 | 0.0266 | 1.82E-05 | 0.0171 | *ATP13A4; ATP13A4-AS1* |
| chr2 | 236812921 | 236812946 | 2 | 0.1188 | 0.0280 | 2.25E-05 | 0.0211 | *AGAP1* |
| chr10 | 106013534 | 106014311 | 8 | 0.0405 | 0.0096 | 2.34E-05 | 0.0219 | *GSTO1* |
| chr12 | 16761299 | 16761419 | 3 | 0.1051 | 0.0251 | 2.84E-05 | 0.0266 | *MGST1; LMO3* |
| chr14 | 37064634 | 37064660 | 2 | 0.1109 | 0.0266 | 3.16E-05 | 0.0296 |  |
| **Birth urine MnBP** | | | | | | | | |
| **chr** | **start** | **end** | **n** | **estimate** | **SE** | ***p*-value** | **adjusted *p*-value** | **Overlapping gene** |
| chr13 | 44833178 | 44833984 | 4 | 0.1543 | 0.0287 | 7.73E-08 | 0.0000 |  |
| chr5 | 1107098 | 1107148 | 2 | 0.2451 | 0.0541 | 5.78E-06 | 0.0058 | *SLC12A7* |
| chr10 | 132887109 | 132887552 | 2 | -0.1924 | 0.0431 | 7.87E-06 | 0.0079 |  |
| chr13 | 95655083 | 95655172 | 2 | 0.1906 | 0.0437 | 1.31E-05 | 0.0131 |  |
| chr15 | 41100308 | 41100798 | 2 | -0.1763 | 0.0415 | 2.16E-05 | 0.0217 | *ZFYVE19* |
| chr19 | 45849751 | 45850053 | 3 | -0.2077 | 0.0496 | 2.81E-05 | 0.0282 | *KLC3* |
| chr21 | 31766922 | 31767469 | 3 | -0.1608 | 0.0391 | 3.97E-05 | 0.0398 |  |
| **Maternal urine DEHP, late pregnancy** | | | | | | | | |
| **chr** | **start** | **end** | **n** | **estimate** | **SE** | ***p*-value** | **adjusted *p*-value** | **Overlapping gene** |
| chr5 | 67730497 | 67730595 | 2 | 0.0516 | 0.0088 | 4.45E-09 | 0.0000 |  |
| chr3 | 182511312 | 182511317 | 2 | 0.1576 | 0.0326 | 1.34E-06 | 0.0018 | *ATP11B* |
| chr11 | 102359753 | 102359781 | 2 | -0.1410 | 0.0311 | 5.95E-06 | 0.0079 | *RP11-315O6.2* |
| chr1 | 235106679 | 235107109 | 2 | 0.0525 | 0.0121 | 1.38E-05 | 0.0183 |  |
| chr18 | 2554720 | 2554914 | 2 | -0.1489 | 0.0351 | 2.17E-05 | 0.0288 | *METTL4* |
| chr1 | 217672412 | 217672626 | 2 | -0.1120 | 0.0265 | 2.47E-05 | 0.0328 | *GPATCH2* |
| chr12 | 53730380 | 53730421 | 3 | 0.0997 | 0.0239 | 3.06E-05 | 0.0406 | *SP7* |
| chr8 | 10331676 | 10331891 | 3 | -0.1121 | 0.0270 | 3.27E-05 | 0.0434 |  |
| chr12 | 26204751 | 26205058 | 2 | -0.1218 | 0.0295 | 3.67E-05 | 0.0488 | *RASSF8* |
| **Birth urine DEHP** | | | | | | | | |
| **chr** | **start** | **end** | **n** | **estimate** | **SE** | ***p*-value** | **adjusted *p*-value** | **Overlapping gene** |
| chr17 | 80928410 | 80929145 | 5 | -0.2015 | 0.0382 | 1.29E-07 | 0.0002 | *B3GNTL1* |
| chr19 | 18700553 | 18700933 | 2 | -0.3134 | 0.0598 | 1.57E-07 | 0.0003 | *CRLF1; C19orf60* |
| chr21 | 42219202 | 42219211 | 2 | 0.2657 | 0.0543 | 1.00E-06 | 0.0018 |  |
| chr3 | 194868750 | 194868790 | 2 | -0.3211 | 0.0663 | 1.26E-06 | 0.0023 | *XXYLT1; XXYLT1-AS2* |
| chr6 | 31461728 | 31461850 | 2 | -0.2332 | 0.0503 | 3.52E-06 | 0.0064 |  |
| chr22 | 47439366 | 47439477 | 2 | -0.2260 | 0.0506 | 7.87E-06 | 0.0142 | *TBC1D22A* |
| chr5 | 177870276 | 177870407 | 4 | -0.2096 | 0.0478 | 1.17E-05 | 0.0212 | *COL23A1* |
| chr4 | 736765 | 737005 | 2 | -0.2465 | 0.0569 | 1.46E-05 | 0.0265 | *PCGF3* |
| chr10 | 50285414 | 50285427 | 2 | -0.2298 | 0.0539 | 2.04E-05 | 0.0369 | *VSTM4* |
| chr8 | 143867129 | 143867383 | 2 | -0.1919 | 0.0454 | 2.38E-05 | 0.0430 | *LY6D; RP11-706C16.8* |

**Supplementary Table 2**. Gene set enrichment analysis result using significant DMRs associated with MEOHP concentration in maternal urine samples during late pregnancy (A: GO terms) and birth urine samples (B: GO terms, C: KEGG pathways).

(A)

|  | **ONTOLOGY** | **TERM** | **N** | **DE** | **P.DE** |
| --- | --- | --- | --- | --- | --- |
| GO:0051182 | BP | coenzyme transport | 8 | 1 | 0.0011 |
| GO:0015851 | BP | nucleobase transport | 8 | 1 | 0.0013 |
| GO:0019852 | BP | L-ascorbic acid metabolic process | 10 | 1 | 0.0020 |
| GO:0035461 | BP | vitamin transmembrane transport | 16 | 1 | 0.0032 |
| GO:0070633 | BP | transepithelial transport | 17 | 1 | 0.0034 |
| GO:1902287 | BP | semaphorin-plexin signaling pathway involved in axon guidance | 10 | 1 | 0.0043 |
| GO:1902285 | BP | semaphorin-plexin signaling pathway involved in neuron projection guidance | 11 | 1 | 0.0048 |
| GO:0051180 | BP | vitamin transport | 39 | 1 | 0.0080 |
| GO:0051181 | BP | cofactor transport | 46 | 1 | 0.0081 |
| GO:0071526 | BP | semaphorin-plexin signaling pathway | 37 | 1 | 0.0115 |
| GO:0006767 | BP | water-soluble vitamin metabolic process | 84 | 1 | 0.0158 |
| GO:0015749 | BP | monosaccharide transmembrane transport | 112 | 1 | 0.0214 |
| GO:0034219 | BP | carbohydrate transmembrane transport | 114 | 1 | 0.0216 |
| GO:0050772 | BP | positive regulation of axonogenesis | 79 | 1 | 0.0233 |
| GO:0006766 | BP | vitamin metabolic process | 130 | 1 | 0.0246 |
| GO:1903825 | BP | organic acid transmembrane transport | 126 | 1 | 0.0263 |
| GO:1905039 | BP | carboxylic acid transmembrane transport | 126 | 1 | 0.0263 |
| GO:0008643 | BP | carbohydrate transport | 145 | 1 | 0.0266 |
| GO:0008360 | BP | regulation of cell shape | 144 | 1 | 0.0363 |
| GO:0010770 | BP | positive regulation of cell morphogenesis involved in differentiation | 142 | 1 | 0.0401 |
| GO:0006814 | BP | sodium ion transport | 208 | 1 | 0.0431 |
| GO:0050770 | BP | regulation of axonogenesis | 172 | 1 | 0.0496 |

(B)

|  | **ONTOLOGY** | **TERM** | **N** | **DE** | **P.DE** |
| --- | --- | --- | --- | --- | --- |
| GO:0043415 | BP | positive regulation of skeletal muscle tissue regeneration | 5 | 1 | 0.0034 |
| GO:0043416 | BP | regulation of skeletal muscle tissue regeneration | 8 | 1 | 0.0047 |
| GO:0014719 | BP | skeletal muscle satellite cell activation | 7 | 1 | 0.0050 |
| GO:0048312 | BP | intracellular distribution of mitochondria | 8 | 1 | 0.0055 |
| GO:2000288 | BP | positive regulation of myoblast proliferation | 9 | 1 | 0.0065 |
| GO:0006382 | BP | adenosine to inosine editing | 9 | 1 | 0.0067 |
| GO:0006048 | BP | UDP-N-acetylglucosamine biosynthetic process | 10 | 1 | 0.0072 |
| GO:0046349 | BP | amino sugar biosynthetic process | 12 | 1 | 0.0085 |
| GO:0099151 | BP | regulation of postsynaptic density assembly | 11 | 1 | 0.0086 |
| GO:0106074 | BP | aminoacyl-tRNA metabolism involved in translational fidelity | 13 | 1 | 0.0090 |
| GO:0099150 | BP | regulation of postsynaptic specialization assembly | 12 | 1 | 0.0092 |
| GO:0150052 | BP | regulation of postsynapse assembly | 12 | 1 | 0.0092 |
| GO:0048311 | BP | mitochondrion distribution | 15 | 1 | 0.0102 |
| GO:0055064 | BP | chloride ion homeostasis | 14 | 1 | 0.0104 |
| GO:0006047 | BP | UDP-N-acetylglucosamine metabolic process | 15 | 1 | 0.0106 |
| GO:1904889 | BP | regulation of excitatory synapse assembly | 14 | 1 | 0.0107 |
| GO:1905874 | BP | regulation of postsynaptic density organization | 15 | 1 | 0.0113 |
| GO:2000291 | BP | regulation of myoblast proliferation | 23 | 1 | 0.0116 |
| GO:0016553 | BP | base conversion or substitution editing | 21 | 1 | 0.0122 |
| GO:0006450 | BP | regulation of translational fidelity | 19 | 1 | 0.0128 |
| GO:0097107 | BP | postsynaptic density assembly | 17 | 1 | 0.0138 |
| GO:0051450 | BP | myoblast proliferation | 27 | 1 | 0.0142 |
| GO:0009226 | BP | nucleotide-sugar biosynthetic process | 22 | 1 | 0.0159 |
| GO:0098698 | BP | postsynaptic specialization assembly | 20 | 1 | 0.0161 |
| GO:0055083 | BP | monovalent inorganic anion homeostasis | 26 | 1 | 0.0180 |
| GO:0006884 | BP | cell volume homeostasis | 27 | 1 | 0.0197 |
| GO:1904861 | BP | excitatory synapse assembly | 25 | 1 | 0.0197 |
| GO:0055075 | BP | potassium ion homeostasis | 29 | 1 | 0.0211 |
| GO:0099068 | BP | postsynapse assembly | 27 | 1 | 0.0216 |
| GO:0097106 | BP | postsynaptic density organization | 29 | 1 | 0.0230 |
| GO:0099084 | BP | postsynaptic specialization organization | 31 | 1 | 0.0245 |
| GO:0006040 | BP | amino sugar metabolic process | 36 | 1 | 0.0255 |
| GO:0009225 | BP | nucleotide-sugar metabolic process | 37 | 1 | 0.0259 |
| GO:0043403 | BP | skeletal muscle tissue regeneration | 40 | 1 | 0.0271 |
| GO:0070316 | BP | regulation of G0 to G1 transition | 39 | 1 | 0.0273 |
| GO:1903671 | BP | negative regulation of sprouting angiogenesis | 66 | 1 | 0.0275 |
| GO:0051646 | BP | mitochondrion localization | 42 | 1 | 0.0285 |
| GO:0045023 | BP | G0 to G1 transition | 41 | 1 | 0.0285 |
| GO:0045197 | BP | establishment or maintenance of epithelial cell apical/basal polarity | 39 | 1 | 0.0287 |
| GO:1990573 | BP | potassium ion import across plasma membrane | 43 | 1 | 0.0299 |
| GO:0035088 | BP | establishment or maintenance of apical/basal cell polarity | 42 | 1 | 0.0306 |
| GO:0061245 | BP | establishment or maintenance of bipolar cell polarity | 42 | 1 | 0.0306 |
| GO:0006418 | BP | tRNA aminoacylation for protein translation | 44 | 1 | 0.0308 |
| GO:0043039 | BP | tRNA aminoacylation | 47 | 1 | 0.0326 |
| GO:0010107 | BP | potassium ion import | 47 | 1 | 0.0331 |
| GO:0043038 | BP | amino acid activation | 48 | 1 | 0.0333 |
| GO:0001937 | BP | negative regulation of endothelial cell proliferation | 69 | 1 | 0.0338 |
| GO:0045843 | BP | negative regulation of striated muscle tissue development | 57 | 1 | 0.0355 |
| GO:0048635 | BP | negative regulation of muscle organ development | 58 | 1 | 0.0362 |
| GO:0090303 | BP | positive regulation of wound healing | 60 | 1 | 0.0372 |
| GO:1901862 | BP | negative regulation of muscle tissue development | 60 | 1 | 0.0375 |
| GO:0097120 | BP | receptor localization to synapse | 51 | 1 | 0.0376 |
| GO:0055081 | BP | anion homeostasis | 56 | 1 | 0.0377 |
| GO:0043113 | BP | receptor clustering | 53 | 1 | 0.0391 |
| GO:1903036 | BP | positive regulation of response to wounding | 71 | 1 | 0.0438 |
| GO:0010596 | BP | negative regulation of endothelial cell migration | 87 | 1 | 0.0439 |
| GO:0070373 | BP | negative regulation of ERK1 and ERK2 cascade | 70 | 1 | 0.0464 |
| GO:0042246 | BP | tissue regeneration | 77 | 1 | 0.0492 |
| GO:0042060 | BP | wound healing | 542 | 2 | 0.0497 |

(C)

|  | **Description** | **N** | **DE** | **P.DE** |
| --- | --- | --- | --- | --- |
| path:hsa01250 | Biosynthesis of nucleotide sugars | 37 | 1 | 0.0257 |
| path:hsa00970 | Aminoacyl-tRNA biosynthesis | 43 | 1 | 0.0299 |
| path:hsa00520 | Amino sugar and nucleotide sugar metabolism | 48 | 1 | 0.0331 |

**Supplementary Table 3**. Result of GO term identification using DMRs associated with MEHHP concentration in (A) maternal urine during late pregnancy and (B) birth urine.

(A)

|  | **ONTOLOGY** | **TERM** | **N** | **DE** | **P.DE** |
| --- | --- | --- | --- | --- | --- |
| GO:0030010 | BP | establishment of cell polarity | 131 | 2 | 0.0010 |
| GO:0060623 | BP | regulation of chromosome condensation | 5 | 1 | 0.0015 |
| GO:0007163 | BP | establishment or maintenance of cell polarity | 204 | 2 | 0.0022 |
| GO:0071850 | BP | mitotic cell cycle arrest | 17 | 1 | 0.0045 |
| GO:1902287 | BP | semaphorin-plexin signaling pathway involved in axon guidance | 10 | 1 | 0.0067 |
| GO:1902285 | BP | semaphorin-plexin signaling pathway involved in neuron projection guidance | 11 | 1 | 0.0075 |
| GO:0000132 | BP | establishment of mitotic spindle orientation | 29 | 1 | 0.0076 |
| GO:0097150 | BP | neuronal stem cell population maintenance | 23 | 1 | 0.0085 |
| GO:0040001 | BP | establishment of mitotic spindle localization | 33 | 1 | 0.0087 |
| GO:0030261 | BP | chromosome condensation | 46 | 1 | 0.0103 |
| GO:0071539 | BP | protein localization to centrosome | 31 | 1 | 0.0104 |
| GO:0051294 | BP | establishment of spindle orientation | 35 | 1 | 0.0106 |
| GO:0090162 | BP | establishment of epithelial cell polarity | 27 | 1 | 0.0108 |
| GO:1905508 | BP | protein localization to microtubule organizing center | 32 | 1 | 0.0109 |
| GO:0046605 | BP | regulation of centrosome cycle | 58 | 1 | 0.0113 |
| GO:0072698 | BP | protein localization to microtubule cytoskeleton | 48 | 1 | 0.0142 |
| GO:0051293 | BP | establishment of spindle localization | 43 | 1 | 0.0146 |
| GO:0044380 | BP | protein localization to cytoskeleton | 53 | 1 | 0.0148 |
| GO:0051653 | BP | spindle localization | 48 | 1 | 0.0152 |
| GO:0071526 | BP | semaphorin-plexin signaling pathway | 37 | 1 | 0.0172 |
| GO:0007098 | BP | centrosome cycle | 121 | 1 | 0.0278 |
| GO:1902850 | BP | microtubule cytoskeleton organization involved in mitosis | 126 | 1 | 0.0308 |
| GO:0031023 | BP | microtubule organizing center organization | 130 | 1 | 0.0313 |
| GO:0050772 | BP | positive regulation of axonogenesis | 79 | 1 | 0.0345 |
| GO:0006323 | BP | DNA packaging | 177 | 1 | 0.0369 |
| GO:0021987 | BP | cerebral cortex development | 111 | 1 | 0.0400 |
| GO:0019827 | BP | stem cell population maintenance | 152 | 1 | 0.0457 |
| GO:0098727 | BP | maintenance of cell number | 154 | 1 | 0.0457 |
| GO:0070507 | BP | regulation of microtubule cytoskeleton organization | 179 | 1 | 0.0487 |

(B)

|  | **ONTOLOGY** | **TERM** | **N** | **DE** | **P.DE** |
| --- | --- | --- | --- | --- | --- |
| GO:0061817 | BP | endoplasmic reticulum-plasma membrane tethering | 6 | 1 | 0.0046 |
| GO:0048312 | BP | intracellular distribution of mitochondria | 8 | 1 | 0.0048 |
| GO:0051643 | BP | endoplasmic reticulum localization | 8 | 1 | 0.0061 |
| GO:0099151 | BP | regulation of postsynaptic density assembly | 11 | 1 | 0.0083 |
| GO:0071804 | BP | cellular potassium ion transport | 209 | 2 | 0.0087 |
| GO:0071805 | BP | potassium ion transmembrane transport | 209 | 2 | 0.0087 |
| GO:0099150 | BP | regulation of postsynaptic specialization assembly | 12 | 1 | 0.0091 |
| GO:0150052 | BP | regulation of postsynapse assembly | 12 | 1 | 0.0091 |
| GO:0048311 | BP | mitochondrion distribution | 15 | 1 | 0.0099 |
| GO:0055064 | BP | chloride ion homeostasis | 14 | 1 | 0.0102 |
| GO:0006813 | BP | potassium ion transport | 232 | 2 | 0.0104 |
| GO:1904889 | BP | regulation of excitatory synapse assembly | 14 | 1 | 0.0105 |
| GO:1905874 | BP | regulation of postsynaptic density organization | 15 | 1 | 0.0113 |
| GO:0097107 | BP | postsynaptic density assembly | 17 | 1 | 0.0136 |
| GO:0098657 | BP | import into cell | 815 | 3 | 0.0157 |
| GO:0098698 | BP | postsynaptic specialization assembly | 20 | 1 | 0.0164 |
| GO:0055083 | BP | monovalent inorganic anion homeostasis | 26 | 1 | 0.0171 |
| GO:0055075 | BP | potassium ion homeostasis | 29 | 1 | 0.0196 |
| GO:0006884 | BP | cell volume homeostasis | 27 | 1 | 0.0199 |
| GO:1904861 | BP | excitatory synapse assembly | 25 | 1 | 0.0202 |
| GO:0099068 | BP | postsynapse assembly | 27 | 1 | 0.0225 |
| GO:0097106 | BP | postsynaptic density organization | 29 | 1 | 0.0236 |
| GO:0099084 | BP | postsynaptic specialization organization | 31 | 1 | 0.0253 |
| GO:0051646 | BP | mitochondrion localization | 42 | 1 | 0.0261 |
| GO:1990573 | BP | potassium ion import across plasma membrane | 43 | 1 | 0.0287 |
| GO:1903671 | BP | negative regulation of sprouting angiogenesis | 66 | 1 | 0.0289 |
| GO:0045197 | BP | establishment or maintenance of epithelial cell apical/basal polarity | 39 | 1 | 0.0294 |
| GO:0035088 | BP | establishment or maintenance of apical/basal cell polarity | 42 | 1 | 0.0313 |
| GO:0061245 | BP | establishment or maintenance of bipolar cell polarity | 42 | 1 | 0.0313 |
| GO:0042274 | BP | ribosomal small subunit biogenesis | 64 | 1 | 0.0322 |
| GO:0010107 | BP | potassium ion import | 47 | 1 | 0.0324 |
| GO:0042273 | BP | ribosomal large subunit biogenesis | 68 | 1 | 0.0326 |
| GO:0055081 | BP | anion homeostasis | 56 | 1 | 0.0335 |
| GO:0001937 | BP | negative regulation of endothelial cell proliferation | 69 | 1 | 0.0338 |
| GO:0043113 | BP | receptor clustering | 53 | 1 | 0.0384 |
| GO:0015672 | BP | monovalent inorganic cation transport | 498 | 2 | 0.0394 |
| GO:0097120 | BP | receptor localization to synapse | 51 | 1 | 0.0402 |
| GO:0006687 | BP | glycosphingolipid metabolic process | 64 | 1 | 0.0406 |
| GO:0070373 | BP | negative regulation of ERK1 and ERK2 cascade | 70 | 1 | 0.0436 |
| GO:0010596 | BP | negative regulation of endothelial cell migration | 87 | 1 | 0.0439 |
| GO:0042733 | BP | embryonic digit morphogenesis | 61 | 1 | 0.0440 |

**Supplementary Table 4**. Gene set enrichment analysis result using significant DMRs associated with MnBP concentration in maternal urine samples during late pregnancy (A: GO terms) and birth urine samples (B: GO terms, C: KEGG pathways).

(A)

|  | **ONTOLOGY** | **TERM** | **N** | **DE** | **P.DE** |
| --- | --- | --- | --- | --- | --- |
| GO:0006891 | BP | intra-Golgi vesicle-mediated transport | 32 | 1 | 0.0074 |
| GO:0030212 | BP | hyaluronan metabolic process | 36 | 1 | 0.0106 |
| GO:1903510 | BP | mucopolysaccharide metabolic process | 108 | 1 | 0.0322 |
| GO:0030203 | BP | glycosaminoglycan metabolic process | 153 | 1 | 0.0474 |
| GO:0006022 | BP | aminoglycan metabolic process | 162 | 1 | 0.0497 |

(B)

|  | **ONTOLOGY** | **TERM** | **N** | **DE** | **P.DE** |
| --- | --- | --- | --- | --- | --- |
| GO:0002501 | BP | peptide antigen assembly with MHC protein complex | 5 | 1 | 0.0012 |
| GO:0002396 | BP | MHC protein complex assembly | 6 | 1 | 0.0015 |
| GO:0099151 | BP | regulation of postsynaptic density assembly | 11 | 1 | 0.0025 |
| GO:0099150 | BP | regulation of postsynaptic specialization assembly | 12 | 1 | 0.0027 |
| GO:0150052 | BP | regulation of postsynapse assembly | 12 | 1 | 0.0027 |
| GO:1905874 | BP | regulation of postsynaptic density organization | 15 | 1 | 0.0030 |
| GO:1904889 | BP | regulation of excitatory synapse assembly | 14 | 1 | 0.0033 |
| GO:0097107 | BP | postsynaptic density assembly | 17 | 1 | 0.0041 |
| GO:0098698 | BP | postsynaptic specialization assembly | 20 | 1 | 0.0047 |
| GO:1904861 | BP | excitatory synapse assembly | 25 | 1 | 0.0057 |
| GO:0000027 | BP | ribosomal large subunit assembly | 28 | 1 | 0.0062 |
| GO:0099068 | BP | postsynapse assembly | 27 | 1 | 0.0062 |
| GO:0097106 | BP | postsynaptic density organization | 29 | 1 | 0.0062 |
| GO:0099084 | BP | postsynaptic specialization organization | 31 | 1 | 0.0066 |
| GO:0042255 | BP | ribosome assembly | 56 | 1 | 0.0123 |
| GO:0070925 | BP | organelle assembly | 806 | 2 | 0.0132 |
| GO:0042273 | BP | ribosomal large subunit biogenesis | 68 | 1 | 0.0150 |
| GO:0022607 | BP | cellular component assembly | 2914 | 3 | 0.0174 |
| GO:0060333 | BP | interferon-gamma-mediated signaling pathway | 87 | 1 | 0.0184 |
| GO:0006283 | BP | transcription-coupled nucleotide-excision repair | 71 | 1 | 0.0186 |
| GO:0034622 | BP | cellular protein-containing complex assembly | 1048 | 2 | 0.0198 |
| GO:0044085 | BP | cellular component biogenesis | 3145 | 3 | 0.0215 |
| GO:0099175 | BP | regulation of postsynapse organization | 92 | 1 | 0.0222 |
| GO:0051963 | BP | regulation of synapse assembly | 97 | 1 | 0.0224 |
| GO:0019886 | BP | antigen processing and presentation of  exogenous peptide antigen via MHC class II | 94 | 1 | 0.0230 |
| GO:0002495 | BP | antigen processing and presentation of  peptide antigen via MHC class II | 97 | 1 | 0.0237 |
| GO:0002504 | BP | antigen processing and presentation of  peptide or polysaccharide antigen via MHC class II | 98 | 1 | 0.0238 |
| GO:0006289 | BP | nucleotide-excision repair | 105 | 1 | 0.0268 |
| GO:0071346 | BP | cellular response to interferon-gamma | 171 | 1 | 0.0315 |
| GO:0009411 | BP | response to UV | 136 | 1 | 0.0332 |
| GO:0034341 | BP | response to interferon-gamma | 189 | 1 | 0.0353 |
| GO:0099173 | BP | postsynapse organization | 149 | 1 | 0.0360 |
| GO:0007416 | BP | synapse assembly | 165 | 1 | 0.0375 |
| GO:0002478 | BP | antigen processing and presentation of exogenous peptide antigen | 167 | 1 | 0.0407 |
| GO:0019884 | BP | antigen processing and presentation of exogenous antigen | 174 | 1 | 0.0414 |
| GO:0050852 | BP | T cell receptor signaling pathway | 186 | 1 | 0.0426 |
| GO:0048002 | BP | antigen processing and presentation of peptide antigen | 180 | 1 | 0.0429 |
| GO:1902115 | BP | regulation of organelle assembly | 186 | 1 | 0.0441 |
| GO:0050807 | BP | regulation of synapse organization | 204 | 1 | 0.0481 |
| GO:0019882 | BP | antigen processing and presentation | 215 | 1 | 0.0486 |

(C)

|  | **Description** | **N** | **DE** | **P.DE** |
| --- | --- | --- | --- | --- |
| path:hsa05310 | Asthma | 27 | 1 | 0.0037 |
| path:hsa05332 | Graft-versus-host disease | 38 | 1 | 0.0063 |
| path:hsa05330 | Allograft rejection | 34 | 1 | 0.0065 |
| path:hsa05320 | Autoimmune thyroid disease | 46 | 1 | 0.0066 |
| path:hsa04940 | Type I diabetes mellitus | 41 | 1 | 0.0083 |
| path:hsa04672 | Intestinal immune network for IgA production | 44 | 1 | 0.0087 |
| path:hsa05150 | Staphylococcus aureus infection | 86 | 1 | 0.0104 |
| path:hsa05321 | Inflammatory bowel disease | 61 | 1 | 0.0120 |
| path:hsa05416 | Viral myocarditis | 55 | 1 | 0.0125 |
| path:hsa04612 | Antigen processing and presentation | 68 | 1 | 0.0130 |
| path:hsa05322 | Systemic lupus erythematosus | 115 | 1 | 0.0135 |
| path:hsa05140 | Leishmaniasis | 70 | 1 | 0.0158 |
| path:hsa05323 | Rheumatoid arthritis | 87 | 1 | 0.0159 |
| path:hsa04640 | Hematopoietic cell lineage | 91 | 1 | 0.0171 |
| path:hsa04658 | Th1 and Th2 cell differentiation | 88 | 1 | 0.0194 |
| path:hsa04659 | Th17 cell differentiation | 103 | 1 | 0.0223 |
| path:hsa05145 | Toxoplasmosis | 106 | 1 | 0.0244 |
| path:hsa03010 | Ribosome | 127 | 1 | 0.0249 |
| path:hsa04145 | Phagosome | 143 | 1 | 0.0299 |
| path:hsa04514 | Cell adhesion molecules | 142 | 1 | 0.0315 |
| path:hsa05164 | Influenza A | 156 | 1 | 0.0333 |
| path:hsa05152 | Tuberculosis | 169 | 1 | 0.0341 |
| path:hsa05171 | Coronavirus disease - COVID-19 | 215 | 1 | 0.0403 |
| path:hsa05169 | Epstein-Barr virus infection | 193 | 1 | 0.0444 |
| path:hsa05166 | Human T-cell leukemia virus 1 infection | 214 | 1 | 0.0495 |

**Supplementary Table 5**. Gene set enrichment analysis result using significant DMRs associated with DEHP concentration in maternal urine samples during late pregnancy (A: GO terms) and birth urine samples (B: GO terms, C: KEGG pathways).

(A)

|  | **ONTOLOGY** | **TERM** | **N** | **DE** | **P.DE** |
| --- | --- | --- | --- | --- | --- |
| GO:0030010 | BP | establishment of cell polarity | 131 | 2 | 0.0010 |
| GO:0060623 | BP | regulation of chromosome condensation | 5 | 1 | 0.0015 |
| GO:0007163 | BP | establishment or maintenance of cell polarity | 204 | 2 | 0.0022 |
| GO:0071850 | BP | mitotic cell cycle arrest | 17 | 1 | 0.0045 |
| GO:1902287 | BP | semaphorin-plexin signaling pathway involved in axon guidance | 10 | 1 | 0.0067 |
| GO:1902285 | BP | semaphorin-plexin signaling pathway involved in neuron projection guidance | 11 | 1 | 0.0075 |
| GO:0000132 | BP | establishment of mitotic spindle orientation | 29 | 1 | 0.0076 |
| GO:0097150 | BP | neuronal stem cell population maintenance | 23 | 1 | 0.0085 |
| GO:0040001 | BP | establishment of mitotic spindle localization | 33 | 1 | 0.0087 |
| GO:0030261 | BP | chromosome condensation | 46 | 1 | 0.0103 |
| GO:0071539 | BP | protein localization to centrosome | 31 | 1 | 0.0104 |
| GO:0051294 | BP | establishment of spindle orientation | 35 | 1 | 0.0106 |
| GO:0090162 | BP | establishment of epithelial cell polarity | 27 | 1 | 0.0108 |
| GO:1905508 | BP | protein localization to microtubule organizing center | 32 | 1 | 0.0109 |
| GO:0046605 | BP | regulation of centrosome cycle | 58 | 1 | 0.0113 |
| GO:0072698 | BP | protein localization to microtubule cytoskeleton | 48 | 1 | 0.0142 |
| GO:0051293 | BP | establishment of spindle localization | 43 | 1 | 0.0146 |
| GO:0044380 | BP | protein localization to cytoskeleton | 53 | 1 | 0.0148 |
| GO:0051653 | BP | spindle localization | 48 | 1 | 0.0152 |
| GO:0071526 | BP | semaphorin-plexin signaling pathway | 37 | 1 | 0.0172 |
| GO:0007098 | BP | centrosome cycle | 121 | 1 | 0.0278 |
| GO:1902850 | BP | microtubule cytoskeleton organization involved in mitosis | 126 | 1 | 0.0308 |
| GO:0031023 | BP | microtubule organizing center organization | 130 | 1 | 0.0313 |
| GO:0050772 | BP | positive regulation of axonogenesis | 79 | 1 | 0.0345 |
| GO:0006323 | BP | DNA packaging | 177 | 1 | 0.0369 |
| GO:0021987 | BP | cerebral cortex development | 111 | 1 | 0.0400 |
| GO:0019827 | BP | stem cell population maintenance | 152 | 1 | 0.0457 |
| GO:0098727 | BP | maintenance of cell number | 154 | 1 | 0.0457 |
| GO:0070507 | BP | regulation of microtubule cytoskeleton organization | 179 | 1 | 0.0487 |

(B)

|  | **ONTOLOGY** | **TERM** | **N** | **DE** | **P.DE** |
| --- | --- | --- | --- | --- | --- |
| GO:0043415 | BP | positive regulation of skeletal muscle tissue regeneration | 5 | 1 | 0.0036 |
| GO:0014719 | BP | skeletal muscle satellite cell activation | 7 | 1 | 0.0054 |
| GO:0043416 | BP | regulation of skeletal muscle tissue regeneration | 8 | 1 | 0.0057 |
| GO:0048312 | BP | intracellular distribution of mitochondria | 8 | 1 | 0.0064 |
| GO:0006382 | BP | adenosine to inosine editing | 9 | 1 | 0.0069 |
| GO:0006048 | BP | UDP-N-acetylglucosamine biosynthetic process | 10 | 1 | 0.0070 |
| GO:2000288 | BP | positive regulation of myoblast proliferation | 9 | 1 | 0.0071 |
| GO:0046349 | BP | amino sugar biosynthetic process | 12 | 1 | 0.0081 |
| GO:0106074 | BP | aminoacyl-tRNA metabolism involved in translational fidelity | 13 | 1 | 0.0089 |
| GO:0099151 | BP | regulation of postsynaptic density assembly | 11 | 1 | 0.0094 |
| GO:0055064 | BP | chloride ion homeostasis | 14 | 1 | 0.0097 |
| GO:0006047 | BP | UDP-N-acetylglucosamine metabolic process | 15 | 1 | 0.0099 |
| GO:0099150 | BP | regulation of postsynaptic specialization assembly | 12 | 1 | 0.0100 |
| GO:0150052 | BP | regulation of postsynapse assembly | 12 | 1 | 0.0100 |
| GO:0031573 | BP | intra-S DNA damage checkpoint | 15 | 1 | 0.0108 |
| GO:0000712 | BP | resolution of meiotic recombination intermediates | 16 | 1 | 0.0109 |
| GO:0048311 | BP | mitochondrion distribution | 15 | 1 | 0.0110 |
| GO:1904889 | BP | regulation of excitatory synapse assembly | 14 | 1 | 0.0114 |
| GO:1905874 | BP | regulation of postsynaptic density organization | 15 | 1 | 0.0129 |
| GO:0006450 | BP | regulation of translational fidelity | 19 | 1 | 0.0135 |
| GO:0097107 | BP | postsynaptic density assembly | 17 | 1 | 0.0144 |
| GO:2000291 | BP | regulation of myoblast proliferation | 23 | 1 | 0.0147 |
| GO:0051307 | BP | meiotic chromosome separation | 23 | 1 | 0.0154 |
| GO:0016553 | BP | base conversion or substitution editing | 21 | 1 | 0.0154 |
| GO:0009226 | BP | nucleotide-sugar biosynthetic process | 22 | 1 | 0.0154 |
| GO:0098698 | BP | postsynaptic specialization assembly | 20 | 1 | 0.0167 |
| GO:0055083 | BP | monovalent inorganic anion homeostasis | 26 | 1 | 0.0173 |
| GO:0051450 | BP | myoblast proliferation | 27 | 1 | 0.0176 |
| GO:0006884 | BP | cell volume homeostasis | 27 | 1 | 0.0195 |
| GO:0031297 | BP | replication fork processing | 30 | 1 | 0.0197 |
| GO:0055075 | BP | potassium ion homeostasis | 29 | 1 | 0.0197 |
| GO:1904861 | BP | excitatory synapse assembly | 25 | 1 | 0.0205 |
| GO:0099068 | BP | postsynapse assembly | 27 | 1 | 0.0224 |
| GO:0006040 | BP | amino sugar metabolic process | 36 | 1 | 0.0249 |
| GO:0097106 | BP | postsynaptic density organization | 29 | 1 | 0.0251 |
| GO:0009225 | BP | nucleotide-sugar metabolic process | 37 | 1 | 0.0259 |
| GO:0045005 | BP | DNA-dependent DNA replication maintenance of fidelity | 39 | 1 | 0.0263 |
| GO:0070316 | BP | regulation of G0 to G1 transition | 39 | 1 | 0.0264 |
| GO:0099084 | BP | postsynaptic specialization organization | 31 | 1 | 0.0265 |
| GO:0045023 | BP | G0 to G1 transition | 41 | 1 | 0.0278 |
| GO:0045197 | BP | establishment or maintenance of epithelial cell apical/basal polarity | 39 | 1 | 0.0278 |
| GO:1990573 | BP | potassium ion import across plasma membrane | 43 | 1 | 0.0283 |
| GO:0006418 | BP | tRNA aminoacylation for protein translation | 44 | 1 | 0.0291 |
| GO:0051646 | BP | mitochondrion localization | 42 | 1 | 0.0292 |
| GO:0035088 | BP | establishment or maintenance of apical/basal cell polarity | 42 | 1 | 0.0296 |
| GO:0061245 | BP | establishment or maintenance of bipolar cell polarity | 42 | 1 | 0.0296 |
| GO:0043403 | BP | skeletal muscle tissue regeneration | 40 | 1 | 0.0296 |
| GO:0010107 | BP | potassium ion import | 47 | 1 | 0.0313 |
| GO:0043039 | BP | tRNA aminoacylation | 47 | 1 | 0.0317 |
| GO:0043038 | BP | amino acid activation | 48 | 1 | 0.0325 |
| GO:0007131 | BP | reciprocal meiotic recombination | 51 | 1 | 0.0339 |
| GO:0035825 | BP | homologous recombination | 52 | 1 | 0.0345 |
| GO:0045843 | BP | negative regulation of striated muscle tissue development | 57 | 1 | 0.0359 |
| GO:0048635 | BP | negative regulation of muscle organ development | 58 | 1 | 0.0366 |
| GO:0097120 | BP | receptor localization to synapse | 51 | 1 | 0.0366 |
| GO:1903671 | BP | negative regulation of sprouting angiogenesis | 66 | 1 | 0.0368 |
| GO:0043113 | BP | receptor clustering | 53 | 1 | 0.0377 |
| GO:0055081 | BP | anion homeostasis | 56 | 1 | 0.0378 |
| GO:1901862 | BP | negative regulation of muscle tissue development | 60 | 1 | 0.0378 |
| GO:0090303 | BP | positive regulation of wound healing | 60 | 1 | 0.0422 |
| GO:0001937 | BP | negative regulation of endothelial cell proliferation | 69 | 1 | 0.0442 |
| GO:0070373 | BP | negative regulation of ERK1 and ERK2 cascade | 70 | 1 | 0.0482 |
| GO:1903036 | BP | positive regulation of response to wounding | 71 | 1 | 0.0490 |

(C)

|  | **Description** | **N** | **DE** | **P.DE** |
| --- | --- | --- | --- | --- |
| path:hsa01250 | Biosynthesis of nucleotide sugars | 37 | 1 | 0.0257 |
| path:hsa00970 | Aminoacyl-tRNA biosynthesis | 43 | 1 | 0.0292 |
| path:hsa03460 | Fanconi anemia pathway | 49 | 1 | 0.0313 |
| path:hsa00520 | Amino sugar and nucleotide sugar metabolism | 48 | 1 | 0.0323 |

**Supplementary Table 6**. Gene set enrichment analysis result using female-specific significant DMRs associated with MEOHP concentration in maternal urine samples during late pregnancy (A: GO terms, B: KEGG pathways) and birth urine samples (C: GO terms, D: KEGG pathways).

(A)

|  | **ONTOLOGY** | **TERM** | **N** | **DE** | **P.DE** |
| --- | --- | --- | --- | --- | --- |
| GO:1901490 | BP | regulation of lymphangiogenesis | 5 | 1 | 0.0031 |
| GO:0030263 | BP | apoptotic chromosome condensation | 5 | 1 | 0.0035 |
| GO:0045657 | BP | positive regulation of monocyte differentiation | 10 | 1 | 0.0064 |
| GO:0001973 | BP | adenosine receptor signaling pathway | 13 | 1 | 0.0076 |
| GO:0002091 | BP | negative regulation of receptor internalization | 13 | 1 | 0.0079 |
| GO:0002031 | BP | G protein-coupled receptor internalization | 13 | 1 | 0.0084 |
| GO:0042983 | BP | amyloid precursor protein biosynthetic process | 14 | 1 | 0.0092 |
| GO:0042984 | BP | regulation of amyloid precursor protein biosynthetic process | 14 | 1 | 0.0092 |
| GO:1900451 | BP | positive regulation of glutamate receptor signaling pathway | 15 | 1 | 0.0094 |
| GO:0001946 | BP | lymphangiogenesis | 16 | 1 | 0.0104 |
| GO:0060716 | BP | labyrinthine layer blood vessel development | 18 | 1 | 0.0108 |
| GO:0002029 | BP | desensitization of G protein-coupled receptor signaling pathway | 17 | 1 | 0.0110 |
| GO:0022401 | BP | negative adaptation of signaling pathway | 17 | 1 | 0.0110 |
| GO:0023058 | BP | adaptation of signaling pathway | 17 | 1 | 0.0110 |
| GO:0035588 | BP | G protein-coupled purinergic receptor signaling pathway | 20 | 1 | 0.0110 |
| GO:0045655 | BP | regulation of monocyte differentiation | 20 | 1 | 0.0119 |
| GO:0036303 | BP | lymph vessel morphogenesis | 19 | 1 | 0.0129 |
| GO:0045745 | BP | positive regulation of G protein-coupled receptor signaling pathway | 31 | 1 | 0.0150 |
| GO:0048261 | BP | negative regulation of receptor-mediated endocytosis | 28 | 1 | 0.0151 |
| GO:0035587 | BP | purinergic receptor signaling pathway | 28 | 1 | 0.0158 |
| GO:0001945 | BP | lymph vessel development | 25 | 1 | 0.0177 |
| GO:0060674 | BP | placenta blood vessel development | 31 | 1 | 0.0177 |
| GO:0030262 | BP | apoptotic nuclear changes | 34 | 1 | 0.0194 |
| GO:0006921 | BP | cellular component disassembly involved in execution phase of apoptosis | 36 | 1 | 0.0207 |
| GO:0030224 | BP | monocyte differentiation | 35 | 1 | 0.0215 |
| GO:1903131 | BP | mononuclear cell differentiation | 35 | 1 | 0.0215 |
| GO:0010559 | BP | regulation of glycoprotein biosynthetic process | 40 | 1 | 0.0229 |
| GO:0015701 | BP | bicarbonate transport | 41 | 1 | 0.0234 |
| GO:0030261 | BP | chromosome condensation | 46 | 1 | 0.0250 |
| GO:1903018 | BP | regulation of glycoprotein metabolic process | 47 | 1 | 0.0260 |
| GO:0060711 | BP | labyrinthine layer development | 43 | 1 | 0.0265 |
| GO:2000772 | BP | regulation of cellular senescence | 50 | 1 | 0.0268 |
| GO:0045744 | BP | negative regulation of G protein-coupled receptor signaling pathway | 48 | 1 | 0.0278 |
| GO:0043537 | BP | negative regulation of blood vessel endothelial cell migration | 69 | 1 | 0.0286 |
| GO:0001937 | BP | negative regulation of endothelial cell proliferation | 69 | 1 | 0.0290 |
| GO:0002763 | BP | positive regulation of myeloid leukocyte differentiation | 53 | 1 | 0.0292 |
| GO:0090342 | BP | regulation of cell aging | 58 | 1 | 0.0311 |
| GO:0002090 | BP | regulation of receptor internalization | 51 | 1 | 0.0317 |
| GO:0022411 | BP | cellular component disassembly | 527 | 2 | 0.0341 |
| GO:0045806 | BP | negative regulation of endocytosis | 67 | 1 | 0.0364 |
| GO:1900449 | BP | regulation of glutamate receptor signaling pathway | 60 | 1 | 0.0379 |
| GO:0042982 | BP | amyloid precursor protein metabolic process | 62 | 1 | 0.0380 |
| GO:0010596 | BP | negative regulation of endothelial cell migration | 87 | 1 | 0.0383 |
| GO:0070126 | BP | mitochondrial translational termination | 89 | 1 | 0.0389 |
| GO:0070125 | BP | mitochondrial translational elongation | 88 | 1 | 0.0391 |
| GO:0090398 | BP | cellular senescence | 74 | 1 | 0.0411 |
| GO:0097194 | BP | execution phase of apoptosis | 80 | 1 | 0.0438 |
| GO:0006415 | BP | translational termination | 102 | 1 | 0.0459 |
| GO:0045639 | BP | positive regulation of myeloid cell differentiation | 86 | 1 | 0.0490 |
| GO:0010633 | BP | negative regulation of epithelial cell migration | 104 | 1 | 0.0491 |

(B)

|  | **Description** | **N** | **DE** | **P.DE** |
| --- | --- | --- | --- | --- |
| path:hsa00520 | Amino sugar and nucleotide sugar metabolism | 48 | 1 | 0.0262 |
| path:hsa00270 | Cysteine and methionine metabolism | 49 | 1 | 0.0265 |
| path:hsa01232 | Nucleotide metabolism | 81 | 1 | 0.0460 |
| path:hsa03015 | mRNA surveillance pathway | 85 | 1 | 0.0469 |

(C)

|  | **ONTOLOGY** | **TERM** | **N** | **DE** | **P.DE** |
| --- | --- | --- | --- | --- | --- |
| GO:0051346 | BP | negative regulation of hydrolase activity | 437 | 3 | 0.0024 |
| GO:0051343 | BP | positive regulation of cyclic-nucleotide phosphodiesterase activity | 5 | 1 | 0.0025 |
| GO:0050917 | BP | sensory perception of umami taste | 6 | 1 | 0.0035 |
| GO:0051342 | BP | regulation of cyclic-nucleotide phosphodiesterase activity | 8 | 1 | 0.0041 |
| GO:0001580 | BP | detection of chemical stimulus involved in sensory perception of bitter taste | 39 | 1 | 0.0042 |
| GO:0006689 | BP | ganglioside catabolic process | 6 | 1 | 0.0046 |
| GO:0033504 | BP | floor plate development | 7 | 1 | 0.0057 |
| GO:0050912 | BP | detection of chemical stimulus involved in sensory perception of taste | 45 | 1 | 0.0066 |
| GO:0009642 | BP | response to light intensity | 10 | 1 | 0.0068 |
| GO:0050913 | BP | sensory perception of bitter taste | 43 | 1 | 0.0072 |
| GO:0035627 | BP | ceramide transport | 13 | 1 | 0.0072 |
| GO:0048669 | BP | collateral sprouting in absence of injury | 5 | 1 | 0.0078 |
| GO:0051639 | BP | actin filament network formation | 7 | 1 | 0.0079 |
| GO:0046479 | BP | glycosphingolipid catabolic process | 13 | 1 | 0.0089 |
| GO:0042362 | BP | fat-soluble vitamin biosynthetic process | 15 | 1 | 0.0092 |
| GO:0009313 | BP | oligosaccharide catabolic process | 13 | 1 | 0.0095 |
| GO:0019377 | BP | glycolipid catabolic process | 15 | 1 | 0.0099 |
| GO:0034375 | BP | high-density lipoprotein particle remodeling | 18 | 1 | 0.0102 |
| GO:0007379 | BP | segment specification | 16 | 1 | 0.0104 |
| GO:0071257 | BP | cellular response to electrical stimulus | 12 | 1 | 0.0105 |
| GO:0046514 | BP | ceramide catabolic process | 19 | 1 | 0.0112 |
| GO:0051336 | BP | regulation of hydrolase activity | 1230 | 4 | 0.0116 |
| GO:0018230 | BP | peptidyl-L-cysteine S-palmitoylation | 19 | 1 | 0.0119 |
| GO:0018231 | BP | peptidyl-S-diacylglycerol-L-cysteine biosynthetic process from peptidyl-cysteine | 19 | 1 | 0.0119 |
| GO:1900029 | BP | positive regulation of ruffle assembly | 15 | 1 | 0.0128 |
| GO:0050908 | BP | detection of light stimulus involved in visual perception | 16 | 1 | 0.0134 |
| GO:0050962 | BP | detection of light stimulus involved in sensory perception | 16 | 1 | 0.0134 |
| GO:0010875 | BP | positive regulation of cholesterol efflux | 16 | 1 | 0.0135 |
| GO:0043086 | BP | negative regulation of catalytic activity | 777 | 3 | 0.0137 |
| GO:0009110 | BP | vitamin biosynthetic process | 23 | 1 | 0.0139 |
| GO:0034368 | BP | protein-lipid complex remodeling | 28 | 1 | 0.0146 |
| GO:0034369 | BP | plasma lipoprotein particle remodeling | 28 | 1 | 0.0146 |
| GO:0034367 | BP | protein-containing complex remodeling | 29 | 1 | 0.0150 |
| GO:0014850 | BP | response to muscle activity | 24 | 1 | 0.0160 |
| GO:0030903 | BP | notochord development | 19 | 1 | 0.0160 |
| GO:0022400 | BP | regulation of rhodopsin mediated signaling pathway | 24 | 1 | 0.0164 |
| GO:0030149 | BP | sphingolipid catabolic process | 29 | 1 | 0.0165 |
| GO:0001573 | BP | ganglioside metabolic process | 19 | 1 | 0.0172 |
| GO:0032373 | BP | positive regulation of sterol transport | 23 | 1 | 0.0177 |
| GO:0032376 | BP | positive regulation of cholesterol transport | 23 | 1 | 0.0177 |
| GO:0016056 | BP | rhodopsin mediated signaling pathway | 26 | 1 | 0.0179 |
| GO:0010874 | BP | regulation of cholesterol efflux | 38 | 1 | 0.0182 |
| GO:0018345 | BP | protein palmitoylation | 31 | 1 | 0.0188 |
| GO:0046466 | BP | membrane lipid catabolic process | 33 | 1 | 0.0190 |
| GO:0050907 | BP | detection of chemical stimulus involved in sensory perception | 394 | 1 | 0.0216 |
| GO:0007603 | BP | phototransduction, visible light | 31 | 1 | 0.0227 |
| GO:0006482 | BP | protein demethylation | 26 | 1 | 0.0228 |
| GO:0008214 | BP | protein dealkylation | 26 | 1 | 0.0228 |
| GO:0050909 | BP | sensory perception of taste | 65 | 1 | 0.0234 |
| GO:0042462 | BP | eye photoreceptor cell development | 35 | 1 | 0.0246 |
| GO:1900027 | BP | regulation of ruffle assembly | 29 | 1 | 0.0255 |
| GO:0071827 | BP | plasma lipoprotein particle organization | 45 | 1 | 0.0267 |
| GO:0033344 | BP | cholesterol efflux | 56 | 1 | 0.0267 |
| GO:0000578 | BP | embryonic axis specification | 33 | 1 | 0.0273 |
| GO:0006470 | BP | protein dephosphorylation | 309 | 2 | 0.0274 |
| GO:0006775 | BP | fat-soluble vitamin metabolic process | 43 | 1 | 0.0277 |
| GO:0018198 | BP | peptidyl-cysteine modification | 43 | 1 | 0.0281 |
| GO:0048668 | BP | collateral sprouting | 25 | 1 | 0.0307 |
| GO:0071825 | BP | protein-lipid complex subunit organization | 49 | 1 | 0.0313 |
| GO:0032371 | BP | regulation of sterol transport | 62 | 1 | 0.0315 |
| GO:0032374 | BP | regulation of cholesterol transport | 62 | 1 | 0.0315 |
| GO:0051602 | BP | response to electrical stimulus | 42 | 1 | 0.0324 |
| GO:0043312 | BP | neutrophil degranulation | 466 | 2 | 0.0330 |
| GO:0002283 | BP | neutrophil activation involved in immune response | 469 | 2 | 0.0332 |
| GO:0097178 | BP | ruffle assembly | 41 | 1 | 0.0333 |
| GO:0009311 | BP | oligosaccharide metabolic process | 47 | 1 | 0.0334 |
| GO:0007602 | BP | phototransduction | 52 | 1 | 0.0336 |
| GO:0042119 | BP | neutrophil activation | 479 | 2 | 0.0345 |
| GO:0044092 | BP | negative regulation of molecular function | 1095 | 3 | 0.0346 |
| GO:0002446 | BP | neutrophil mediated immunity | 480 | 2 | 0.0346 |
| GO:0042461 | BP | photoreceptor cell development | 46 | 1 | 0.0349 |
| GO:0036230 | BP | granulocyte activation | 485 | 2 | 0.0350 |
| GO:0001754 | BP | eye photoreceptor cell differentiation | 46 | 1 | 0.0353 |
| GO:0009584 | BP | detection of visible light | 54 | 1 | 0.0369 |
| GO:0034260 | BP | negative regulation of GTPase activity | 44 | 1 | 0.0384 |
| GO:0042886 | BP | amide transport | 1965 | 4 | 0.0388 |
| GO:0043299 | BP | leukocyte degranulation | 513 | 2 | 0.0396 |
| GO:0002275 | BP | myeloid cell activation involved in immune response | 523 | 2 | 0.0409 |
| GO:0032370 | BP | positive regulation of lipid transport | 61 | 1 | 0.0415 |
| GO:0070988 | BP | demethylation | 64 | 1 | 0.0416 |
| GO:0002444 | BP | myeloid leukocyte mediated immunity | 530 | 2 | 0.0416 |
| GO:0032515 | BP | negative regulation of phosphoprotein phosphatase activity | 58 | 1 | 0.0427 |
| GO:0031529 | BP | ruffle organization | 52 | 1 | 0.0433 |
| GO:0014823 | BP | response to activity | 65 | 1 | 0.0448 |
| GO:0009583 | BP | detection of light stimulus | 66 | 1 | 0.0450 |
| GO:0009593 | BP | detection of chemical stimulus | 429 | 1 | 0.0454 |
| GO:0030317 | BP | flagellated sperm motility | 82 | 1 | 0.0473 |
| GO:0097722 | BP | sperm motility | 82 | 1 | 0.0473 |
| GO:0035308 | BP | negative regulation of protein dephosphorylation | 63 | 1 | 0.0480 |
| GO:0006687 | BP | glycosphingolipid metabolic process | 64 | 1 | 0.0482 |
| GO:0046530 | BP | photoreceptor cell differentiation | 62 | 1 | 0.0489 |
| GO:0006497 | BP | protein lipidation | 83 | 1 | 0.0489 |

(D)

|  | **Description** | **N** | **DE** | **P.DE** |
| --- | --- | --- | --- | --- |
| path:hsa00511 | Other glycan degradation | 18 | 1 | 0.0134 |
| path:hsa04744 | Phototransduction | 27 | 1 | 0.0153 |
| path:hsa04940 | Type I diabetes mellitus | 41 | 1 | 0.0257 |
| path:hsa04979 | Cholesterol metabolism | 51 | 1 | 0.0329 |
| path:hsa00600 | Sphingolipid metabolism | 52 | 1 | 0.0363 |
| path:hsa03320 | PPAR signaling pathway | 72 | 1 | 0.0470 |

**Supplementary Table 7**. Gene set enrichment analysis result using female-specific significant DMRs associated with MEHHP concentration in maternal urine samples during late pregnancy (A: GO terms) and birth urine samples (B: GO terms, C: KEGG pathways).

(A)

|  | **ONTOLOGY** | **TERM** | **N** | **DE** | **P.DE** |
| --- | --- | --- | --- | --- | --- |
| GO:1901490 | BP | regulation of lymphangiogenesis | 5 | 1 | 0.0010 |
| GO:0001946 | BP | lymphangiogenesis | 16 | 1 | 0.0035 |
| GO:0060716 | BP | labyrinthine layer blood vessel development | 18 | 1 | 0.0041 |
| GO:0036303 | BP | lymph vessel morphogenesis | 19 | 1 | 0.0043 |
| GO:0001945 | BP | lymph vessel development | 25 | 1 | 0.0060 |
| GO:0060674 | BP | placenta blood vessel development | 31 | 1 | 0.0068 |
| GO:0060711 | BP | labyrinthine layer development | 43 | 1 | 0.0092 |
| GO:2000772 | BP | regulation of cellular senescence | 50 | 1 | 0.0104 |
| GO:0001937 | BP | negative regulation of endothelial cell proliferation | 69 | 1 | 0.0110 |
| GO:0090342 | BP | regulation of cell aging | 58 | 1 | 0.0121 |
| GO:0043537 | BP | negative regulation of blood vessel endothelial cell migration | 69 | 1 | 0.0122 |
| GO:0090398 | BP | cellular senescence | 74 | 1 | 0.0153 |
| GO:0010596 | BP | negative regulation of endothelial cell migration | 87 | 1 | 0.0153 |
| GO:0001892 | BP | embryonic placenta development | 83 | 1 | 0.0174 |
| GO:0010633 | BP | negative regulation of epithelial cell migration | 104 | 1 | 0.0192 |
| GO:0007569 | BP | cell aging | 112 | 1 | 0.0233 |
| GO:0043535 | BP | regulation of blood vessel endothelial cell migration | 143 | 1 | 0.0268 |
| GO:0050680 | BP | negative regulation of epithelial cell proliferation | 152 | 1 | 0.0275 |
| GO:0016525 | BP | negative regulation of angiogenesis | 158 | 1 | 0.0279 |
| GO:2000181 | BP | negative regulation of blood vessel morphogenesis | 160 | 1 | 0.0283 |
| GO:0001890 | BP | placenta development | 145 | 1 | 0.0299 |
| GO:0001936 | BP | regulation of endothelial cell proliferation | 166 | 1 | 0.0301 |
| GO:1901343 | BP | negative regulation of vasculature development | 172 | 1 | 0.0313 |
| GO:0043534 | BP | blood vessel endothelial cell migration | 166 | 1 | 0.0316 |
| GO:0001935 | BP | endothelial cell proliferation | 180 | 1 | 0.0332 |
| GO:0010594 | BP | regulation of endothelial cell migration | 215 | 1 | 0.0409 |
| GO:0007050 | BP | cell cycle arrest | 232 | 1 | 0.0437 |
| GO:0043542 | BP | endothelial cell migration | 256 | 1 | 0.0485 |

(B)

|  | ONTOLOGY | TERM | N | DE | P.DE |
| --- | --- | --- | --- | --- | --- |
| GO:0031848 | BP | protection from non-homologous end joining at telomere | 10 | 1 | 0.0027 |
| GO:0009650 | BP | UV protection | 13 | 1 | 0.0032 |
| GO:0031999 | BP | negative regulation of fatty acid beta-oxidation | 5 | 1 | 0.0032 |
| GO:0043247 | BP | telomere maintenance in response to DNA damage | 13 | 1 | 0.0037 |
| GO:0000712 | BP | resolution of meiotic recombination intermediates | 16 | 1 | 0.0038 |
| GO:1904354 | BP | negative regulation of telomere capping | 8 | 1 | 0.0039 |
| GO:0046322 | BP | negative regulation of fatty acid oxidation | 10 | 1 | 0.0047 |
| GO:0044092 | BP | negative regulation of molecular function | 1095 | 3 | 0.0048 |
| GO:0000722 | BP | telomere maintenance via recombination | 13 | 1 | 0.0049 |
| GO:0006853 | BP | carnitine shuttle | 10 | 1 | 0.0049 |
| GO:0018230 | BP | peptidyl-L-cysteine S-palmitoylation | 19 | 1 | 0.0057 |
| GO:0018231 | BP | peptidyl-S-diacylglycerol-L-cysteine biosynthetic process  from peptidyl-cysteine | 19 | 1 | 0.0057 |
| GO:0051307 | BP | meiotic chromosome separation | 23 | 1 | 0.0058 |
| GO:0006293 | BP | nucleotide-excision repair, preincision complex stabilization | 19 | 1 | 0.0066 |
| GO:0006295 | BP | nucleotide-excision repair, DNA incision, 3'-to lesion | 19 | 1 | 0.0066 |
| GO:0070911 | BP | global genome nucleotide-excision repair | 23 | 1 | 0.0068 |
| GO:0006312 | BP | mitotic recombination | 22 | 1 | 0.0076 |
| GO:0045922 | BP | negative regulation of fatty acid metabolic process | 31 | 1 | 0.0077 |
| GO:0014850 | BP | response to muscle activity | 24 | 1 | 0.0079 |
| GO:0050995 | BP | negative regulation of lipid catabolic process | 23 | 1 | 0.0083 |
| GO:1902001 | BP | fatty acid transmembrane transport | 15 | 1 | 0.0084 |
| GO:0010884 | BP | positive regulation of lipid storage | 22 | 1 | 0.0084 |
| GO:0000462 | BP | maturation of SSU-rRNA from tricistronic rRNA transcript  (SSU-rRNA, 5.8S rRNA, LSU-rRNA) | 33 | 1 | 0.0088 |
| GO:0018345 | BP | protein palmitoylation | 31 | 1 | 0.0090 |
| GO:1904357 | BP | negative regulation of telomere maintenance via telomere lengthening | 24 | 1 | 0.0094 |
| GO:0031998 | BP | regulation of fatty acid beta-oxidation | 17 | 1 | 0.0099 |
| GO:0006296 | BP | nucleotide-excision repair, DNA incision, 5'-to lesion | 35 | 1 | 0.0101 |
| GO:0030490 | BP | maturation of SSU-rRNA | 44 | 1 | 0.0103 |
| GO:0033683 | BP | nucleotide-excision repair, DNA incision | 37 | 1 | 0.0109 |
| GO:0006084 | BP | acetyl-CoA metabolic process | 36 | 1 | 0.0118 |
| GO:1904353 | BP | regulation of telomere capping | 26 | 1 | 0.0126 |
| GO:0042274 | BP | ribosomal small subunit biogenesis | 64 | 1 | 0.0133 |
| GO:0032205 | BP | negative regulation of telomere maintenance | 33 | 1 | 0.0137 |
| GO:0007131 | BP | reciprocal meiotic recombination | 51 | 1 | 0.0139 |
| GO:0035825 | BP | homologous recombination | 52 | 1 | 0.0142 |
| GO:0018198 | BP | peptidyl-cysteine modification | 43 | 1 | 0.0143 |
| GO:0046320 | BP | regulation of fatty acid oxidation | 29 | 1 | 0.0148 |
| GO:0036297 | BP | interstrand cross-link repair | 49 | 1 | 0.0151 |
| GO:0032365 | BP | intracellular lipid transport | 42 | 1 | 0.0158 |
| GO:0016233 | BP | telomere capping | 53 | 1 | 0.0163 |
| GO:0097009 | BP | energy homeostasis | 34 | 1 | 0.0175 |
| GO:0043170 | BP | macromolecule metabolic process | 9578 | 6 | 0.0176 |
| GO:0043392 | BP | negative regulation of DNA binding | 51 | 1 | 0.0184 |
| GO:0045540 | BP | regulation of cholesterol biosynthetic process | 49 | 1 | 0.0194 |
| GO:0106118 | BP | regulation of sterol biosynthetic process | 49 | 1 | 0.0194 |
| GO:0006283 | BP | transcription-coupled nucleotide-excision repair | 71 | 1 | 0.0199 |
| GO:0010883 | BP | regulation of lipid storage | 46 | 1 | 0.0204 |
| GO:0015909 | BP | long-chain fatty acid transport | 67 | 1 | 0.0204 |
| GO:0050994 | BP | regulation of lipid catabolic process | 53 | 1 | 0.0205 |
| GO:1990542 | BP | mitochondrial transmembrane transport | 95 | 1 | 0.0207 |
| GO:0045132 | BP | meiotic chromosome segregation | 88 | 1 | 0.0212 |
| GO:0034260 | BP | negative regulation of GTPase activity | 44 | 1 | 0.0220 |
| GO:0035384 | BP | thioester biosynthetic process | 50 | 1 | 0.0223 |
| GO:0071616 | BP | acyl-CoA biosynthetic process | 50 | 1 | 0.0223 |
| GO:1904356 | BP | regulation of telomere maintenance via telomere lengthening | 58 | 1 | 0.0226 |
| GO:0014823 | BP | response to activity | 65 | 1 | 0.0231 |
| GO:0060421 | BP | positive regulation of heart growth | 47 | 1 | 0.0235 |
| GO:0006497 | BP | protein lipidation | 83 | 1 | 0.0238 |
| GO:0006695 | BP | cholesterol biosynthetic process | 71 | 1 | 0.0240 |
| GO:1902653 | BP | secondary alcohol biosynthetic process | 72 | 1 | 0.0240 |
| GO:0090181 | BP | regulation of cholesterol metabolic process | 62 | 1 | 0.0241 |
| GO:0042158 | BP | lipoprotein biosynthetic process | 88 | 1 | 0.0243 |
| GO:0042592 | BP | homeostatic process | 1836 | 3 | 0.0245 |
| GO:0034644 | BP | cellular response to UV | 79 | 1 | 0.0248 |
| GO:0006303 | BP | double-strand break repair via nonhomologous end joining | 80 | 1 | 0.0250 |
| GO:0006635 | BP | fatty acid beta-oxidation | 71 | 1 | 0.0253 |
| GO:0006807 | BP | nitrogen compound metabolic process | 9928 | 6 | 0.0254 |
| GO:0016126 | BP | sterol biosynthetic process | 77 | 1 | 0.0261 |
| GO:0019915 | BP | lipid storage | 68 | 1 | 0.0270 |
| GO:0046622 | BP | positive regulation of organ growth | 56 | 1 | 0.0271 |
| GO:0000726 | BP | non-recombinational repair | 86 | 1 | 0.0271 |
| GO:0043086 | BP | negative regulation of catalytic activity | 777 | 2 | 0.0280 |
| GO:2001021 | BP | negative regulation of response to DNA damage stimulus | 74 | 1 | 0.0280 |
| GO:0045833 | BP | negative regulation of lipid metabolic process | 90 | 1 | 0.0280 |
| GO:0033866 | BP | nucleoside bisphosphate biosynthetic process | 64 | 1 | 0.0285 |
| GO:0034030 | BP | ribonucleoside bisphosphate biosynthetic process | 64 | 1 | 0.0285 |
| GO:0034033 | BP | purine nucleoside bisphosphate biosynthetic process | 64 | 1 | 0.0285 |
| GO:0010833 | BP | telomere maintenance via telomere lengthening | 78 | 1 | 0.0287 |
| GO:0007127 | BP | meiosis I | 112 | 1 | 0.0288 |
| GO:0032204 | BP | regulation of telomere maintenance | 75 | 1 | 0.0289 |
| GO:0019217 | BP | regulation of fatty acid metabolic process | 91 | 1 | 0.0289 |
| GO:1905954 | BP | positive regulation of lipid localization | 82 | 1 | 0.0294 |
| GO:0015908 | BP | fatty acid transport | 93 | 1 | 0.0300 |
| GO:0061982 | BP | meiosis I cell cycle process | 117 | 1 | 0.0301 |
| GO:0006289 | BP | nucleotide-excision repair | 105 | 1 | 0.0308 |
| GO:0051304 | BP | chromosome separation | 88 | 1 | 0.0318 |
| GO:0035773 | BP | insulin secretion involved in cellular response to glucose stimulus | 62 | 1 | 0.0319 |
| GO:1902930 | BP | regulation of alcohol biosynthetic process | 80 | 1 | 0.0322 |
| GO:0044238 | BP | primary metabolic process | 10381 | 6 | 0.0330 |
| GO:0006637 | BP | acyl-CoA metabolic process | 99 | 1 | 0.0337 |
| GO:0035383 | BP | thioester metabolic process | 99 | 1 | 0.0337 |
| GO:0050810 | BP | regulation of steroid biosynthetic process | 94 | 1 | 0.0338 |
| GO:0062014 | BP | negative regulation of small molecule metabolic process | 104 | 1 | 0.0343 |
| GO:0019395 | BP | fatty acid oxidation | 98 | 1 | 0.0346 |
| GO:0042157 | BP | lipoprotein metabolic process | 124 | 1 | 0.0348 |
| GO:0034440 | BP | lipid oxidation | 100 | 1 | 0.0349 |
| GO:0060420 | BP | regulation of heart growth | 78 | 1 | 0.0355 |
| GO:0009062 | BP | fatty acid catabolic process | 107 | 1 | 0.0357 |
| GO:0031329 | BP | regulation of cellular catabolic process | 833 | 2 | 0.0359 |
| GO:0051289 | BP | protein homotetramerization | 92 | 1 | 0.0362 |
| GO:0044237 | BP | cellular metabolic process | 10500 | 6 | 0.0366 |
| GO:0071482 | BP | cellular response to light stimulus | 119 | 1 | 0.0378 |
| GO:0006364 | BP | rRNA processing | 206 | 1 | 0.0391 |
| GO:0051649 | BP | establishment of localization in cell | 2128 | 3 | 0.0398 |
| GO:0000724 | BP | double-strand break repair via homologous recombination | 125 | 1 | 0.0399 |
| GO:0000725 | BP | recombinational repair | 126 | 1 | 0.0400 |
| GO:0071704 | BP | organic substance metabolic process | 11081 | 6 | 0.0406 |
| GO:0019218 | BP | regulation of steroid metabolic process | 123 | 1 | 0.0407 |
| GO:0033865 | BP | nucleoside bisphosphate metabolic process | 132 | 1 | 0.0423 |
| GO:0033875 | BP | ribonucleoside bisphosphate metabolic process | 132 | 1 | 0.0423 |
| GO:0034032 | BP | purine nucleoside bisphosphate metabolic process | 132 | 1 | 0.0423 |
| GO:0006612 | BP | protein targeting to membrane | 185 | 1 | 0.0436 |
| GO:0072329 | BP | monocarboxylic acid catabolic process | 131 | 1 | 0.0437 |
| GO:0006633 | BP | fatty acid biosynthetic process | 158 | 1 | 0.0441 |
| GO:0009411 | BP | response to UV | 136 | 1 | 0.0443 |
| GO:0051053 | BP | negative regulation of DNA metabolic process | 143 | 1 | 0.0445 |
| GO:0010906 | BP | regulation of glucose metabolic process | 110 | 1 | 0.0449 |
| GO:0009894 | BP | regulation of catabolic process | 935 | 2 | 0.0468 |
| GO:0051101 | BP | regulation of DNA binding | 120 | 1 | 0.0473 |
| GO:0140013 | BP | meiotic nuclear division | 170 | 1 | 0.0475 |
| GO:0046620 | BP | regulation of organ growth | 104 | 1 | 0.0482 |
| GO:2001251 | BP | negative regulation of chromosome organization | 137 | 1 | 0.0486 |
| GO:0060419 | BP | heart growth | 105 | 1 | 0.0488 |
| GO:0035335 | BP | peptidyl-tyrosine dephosphorylation | 97 | 1 | 0.0496 |
| GO:0000723 | BP | telomere maintenance | 153 | 1 | 0.0498 |

(C)

|  | **Description** | **N** | **DE** | **P.DE** |
| --- | --- | --- | --- | --- |
| path:hsa00061 | Fatty acid biosynthesis | 16 | 1 | 0.0096 |
| path:hsa00640 | Propanoate metabolism | 32 | 1 | 0.0124 |
| path:hsa04940 | Type I diabetes mellitus | 41 | 1 | 0.0127 |
| path:hsa03420 | Nucleotide excision repair | 43 | 1 | 0.0128 |
| path:hsa00620 | Pyruvate metabolism | 46 | 1 | 0.0129 |
| path:hsa03460 | Fanconi anemia pathway | 49 | 1 | 0.0165 |
| path:hsa04920 | Adipocytokine signaling pathway | 66 | 1 | 0.0320 |
| path:hsa04922 | Glucagon signaling pathway | 99 | 1 | 0.0435 |
| path:hsa04936 | Alcoholic liver disease | 136 | 1 | 0.0470 |

**Supplementary Table 8**. Gene set enrichment analysis result using female-specific significant DMRs associated with MnBP concentration in maternal urine samples during late pregnancy (A: GO terms, B: KEGG pathways) and birth urine samples (C: GO terms, D: KEGG pathways).

(A)

|  | **ONTOLOGY** | **TERM** | **N** | **DE** | **P.DE** |
| --- | --- | --- | --- | --- | --- |
| GO:0051145 | BP | smooth muscle cell differentiation | 70 | 2 | 0.0008 |
| GO:0051963 | BP | regulation of synapse assembly | 97 | 2 | 0.0024 |
| GO:0097647 | BP | amylin receptor signaling pathway | 6 | 1 | 0.0033 |
| GO:2001016 | BP | positive regulation of skeletal muscle cell differentiation | 8 | 1 | 0.0039 |
| GO:0045906 | BP | negative regulation of vasoconstriction | 6 | 1 | 0.0039 |
| GO:0010694 | BP | positive regulation of alkaline phosphatase activity | 6 | 1 | 0.0040 |
| GO:0006701 | BP | progesterone biosynthetic process | 7 | 1 | 0.0041 |
| GO:0033034 | BP | positive regulation of myeloid cell apoptotic process | 8 | 1 | 0.0043 |
| GO:0007189 | BP | adenylate cyclase-activating G protein-coupled receptor signaling pathway | 135 | 2 | 0.0043 |
| GO:0097646 | BP | calcitonin family receptor signaling pathway | 7 | 1 | 0.0043 |
| GO:0071864 | BP | positive regulation of cell proliferation in bone marrow | 8 | 1 | 0.0048 |
| GO:0071838 | BP | cell proliferation in bone marrow | 9 | 1 | 0.0049 |
| GO:0071863 | BP | regulation of cell proliferation in bone marrow | 9 | 1 | 0.0051 |
| GO:0008277 | BP | regulation of G protein-coupled receptor signaling pathway | 141 | 2 | 0.0051 |
| GO:0072102 | BP | glomerulus morphogenesis | 8 | 1 | 0.0051 |
| GO:0060297 | BP | regulation of sarcomere organization | 9 | 1 | 0.0051 |
| GO:0000187 | BP | activation of MAPK activity | 145 | 2 | 0.0055 |
| GO:1904753 | BP | negative regulation of vascular associated smooth muscle cell migration | 17 | 1 | 0.0056 |
| GO:0010692 | BP | regulation of alkaline phosphatase activity | 9 | 1 | 0.0058 |
| GO:1904354 | BP | negative regulation of telomere capping | 8 | 1 | 0.0060 |
| GO:0097084 | BP | vascular smooth muscle cell development | 9 | 1 | 0.0061 |
| GO:0034454 | BP | microtubule anchoring at centrosome | 9 | 1 | 0.0064 |
| GO:1905563 | BP | negative regulation of vascular endothelial cell proliferation | 23 | 1 | 0.0064 |
| GO:1903367 | BP | positive regulation of fear response | 7 | 1 | 0.0065 |
| GO:2000987 | BP | positive regulation of behavioral fear response | 7 | 1 | 0.0065 |
| GO:2001214 | BP | positive regulation of vasculogenesis | 10 | 1 | 0.0066 |
| GO:0048703 | BP | embryonic viscerocranium morphogenesis | 10 | 1 | 0.0066 |
| GO:0002634 | BP | regulation of germinal center formation | 9 | 1 | 0.0067 |
| GO:0090129 | BP | positive regulation of synapse maturation | 9 | 1 | 0.0068 |
| GO:0003139 | BP | secondary heart field specification | 9 | 1 | 0.0068 |
| GO:0007416 | BP | synapse assembly | 165 | 2 | 0.0069 |
| GO:0055001 | BP | muscle cell development | 181 | 2 | 0.0070 |
| GO:0090073 | BP | positive regulation of protein homodimerization activity | 9 | 1 | 0.0074 |
| GO:0046886 | BP | positive regulation of hormone biosynthetic process | 11 | 1 | 0.0076 |
| GO:0072393 | BP | microtubule anchoring at microtubule organizing center | 11 | 1 | 0.0076 |
| GO:0043116 | BP | negative regulation of vascular permeability | 13 | 1 | 0.0077 |
| GO:2000109 | BP | regulation of macrophage apoptotic process | 10 | 1 | 0.0078 |
| GO:0002031 | BP | G protein-coupled receptor internalization | 13 | 1 | 0.0081 |
| GO:0019933 | BP | cAMP-mediated signaling | 183 | 2 | 0.0081 |
| GO:1903365 | BP | regulation of fear response | 9 | 1 | 0.0083 |
| GO:2000822 | BP | regulation of behavioral fear response | 9 | 1 | 0.0083 |
| GO:0071374 | BP | cellular response to parathyroid hormone stimulus | 8 | 1 | 0.0084 |
| GO:0003211 | BP | cardiac ventricle formation | 10 | 1 | 0.0088 |
| GO:0071107 | BP | response to parathyroid hormone | 10 | 1 | 0.0091 |
| GO:0060712 | BP | spongiotrophoblast layer development | 11 | 1 | 0.0093 |
| GO:0072160 | BP | nephron tubule epithelial cell differentiation | 14 | 1 | 0.0094 |
| GO:0006959 | BP | humoral immune response | 231 | 2 | 0.0094 |
| GO:0042448 | BP | progesterone metabolic process | 16 | 1 | 0.0095 |
| GO:0090128 | BP | regulation of synapse maturation | 14 | 1 | 0.0099 |
| GO:0060670 | BP | branching involved in labyrinthine layer morphogenesis | 12 | 1 | 0.0100 |
| GO:0032352 | BP | positive regulation of hormone metabolic process | 15 | 1 | 0.0101 |
| GO:0003207 | BP | cardiac chamber formation | 12 | 1 | 0.0101 |
| GO:0035303 | BP | regulation of dephosphorylation | 201 | 2 | 0.0101 |
| GO:0050807 | BP | regulation of synapse organization | 204 | 2 | 0.0102 |
| GO:0071888 | BP | macrophage apoptotic process | 12 | 1 | 0.0103 |
| GO:0019935 | BP | cyclic-nucleotide-mediated signaling | 209 | 2 | 0.0105 |
| GO:0003128 | BP | heart field specification | 14 | 1 | 0.0106 |
| GO:2001212 | BP | regulation of vasculogenesis | 14 | 1 | 0.0108 |
| GO:0002467 | BP | germinal center formation | 15 | 1 | 0.0108 |
| GO:0050803 | BP | regulation of synapse structure or activity | 213 | 2 | 0.0109 |
| GO:0007188 | BP | adenylate cyclase-modulating G protein-coupled receptor signaling pathway | 215 | 2 | 0.0110 |
| GO:0002029 | BP | desensitization of G protein-coupled receptor signaling pathway | 17 | 1 | 0.0113 |
| GO:0022401 | BP | negative adaptation of signaling pathway | 17 | 1 | 0.0113 |
| GO:0023058 | BP | adaptation of signaling pathway | 17 | 1 | 0.0113 |
| GO:0055012 | BP | ventricular cardiac muscle cell differentiation | 16 | 1 | 0.0120 |
| GO:0010893 | BP | positive regulation of steroid biosynthetic process | 21 | 1 | 0.0128 |
| GO:0035556 | BP | intracellular signal transduction | 2812 | 6 | 0.0133 |
| GO:0007187 | BP | G protein-coupled receptor signaling pathway,  coupled to cyclic nucleotide second messenger | 246 | 2 | 0.0134 |
| GO:2001014 | BP | regulation of skeletal muscle cell differentiation | 21 | 1 | 0.0134 |
| GO:0010566 | BP | regulation of ketone biosynthetic process | 16 | 1 | 0.0134 |
| GO:2000727 | BP | positive regulation of cardiac muscle cell differentiation | 28 | 1 | 0.0144 |
| GO:0030220 | BP | platelet formation | 19 | 1 | 0.0144 |
| GO:0036344 | BP | platelet morphogenesis | 19 | 1 | 0.0144 |
| GO:0043406 | BP | positive regulation of MAP kinase activity | 246 | 2 | 0.0147 |
| GO:0071498 | BP | cellular response to fluid shear stress | 20 | 1 | 0.0149 |
| GO:0042693 | BP | muscle cell fate commitment | 18 | 1 | 0.0149 |
| GO:0035809 | BP | regulation of urine volume | 21 | 1 | 0.0155 |
| GO:0035584 | BP | calcium-mediated signaling using intracellular calcium source | 20 | 1 | 0.0157 |
| GO:1904706 | BP | negative regulation of vascular smooth muscle cell proliferation | 32 | 1 | 0.0158 |
| GO:0003299 | BP | muscle hypertrophy in response to stress | 31 | 1 | 0.0160 |
| GO:0014887 | BP | cardiac muscle adaptation | 31 | 1 | 0.0160 |
| GO:0014898 | BP | cardiac muscle hypertrophy in response to stress | 31 | 1 | 0.0160 |
| GO:0006925 | BP | inflammatory cell apoptotic process | 22 | 1 | 0.0164 |
| GO:1904738 | BP | vascular associated smooth muscle cell migration | 39 | 1 | 0.0165 |
| GO:1904752 | BP | regulation of vascular associated smooth muscle cell migration | 39 | 1 | 0.0165 |
| GO:0034453 | BP | microtubule anchoring | 24 | 1 | 0.0166 |
| GO:0014912 | BP | negative regulation of smooth muscle cell migration | 33 | 1 | 0.0169 |
| GO:0060713 | BP | labyrinthine layer morphogenesis | 21 | 1 | 0.0169 |
| GO:2000311 | BP | regulation of AMPA receptor activity | 23 | 1 | 0.0170 |
| GO:2000108 | BP | positive regulation of leukocyte apoptotic process | 28 | 1 | 0.0178 |
| GO:0048643 | BP | positive regulation of skeletal muscle tissue development | 26 | 1 | 0.0179 |
| GO:0008209 | BP | androgen metabolic process | 28 | 1 | 0.0181 |
| GO:0043496 | BP | regulation of protein homodimerization activity | 20 | 1 | 0.0186 |
| GO:0045663 | BP | positive regulation of myoblast differentiation | 22 | 1 | 0.0186 |
| GO:0060045 | BP | positive regulation of cardiac muscle cell proliferation | 33 | 1 | 0.0187 |
| GO:0002026 | BP | regulation of the force of heart contraction | 26 | 1 | 0.0187 |
| GO:0101023 | BP | vascular endothelial cell proliferation | 43 | 1 | 0.0188 |
| GO:1905562 | BP | regulation of vascular endothelial cell proliferation | 43 | 1 | 0.0188 |
| GO:0060914 | BP | heart formation | 26 | 1 | 0.0190 |
| GO:0060074 | BP | synapse maturation | 26 | 1 | 0.0191 |
| GO:0030318 | BP | melanocyte differentiation | 25 | 1 | 0.0192 |
| GO:0001958 | BP | endochondral ossification | 27 | 1 | 0.0195 |
| GO:0036075 | BP | replacement ossification | 27 | 1 | 0.0195 |
| GO:0010460 | BP | positive regulation of heart rate | 26 | 1 | 0.0196 |
| GO:0006700 | BP | C21-steroid hormone biosynthetic process | 26 | 1 | 0.0197 |
| GO:0045940 | BP | positive regulation of steroid metabolic process | 30 | 1 | 0.0197 |
| GO:0050790 | BP | regulation of catalytic activity | 2220 | 5 | 0.0198 |
| GO:0035886 | BP | vascular smooth muscle cell differentiation | 37 | 1 | 0.0199 |
| GO:0060536 | BP | cartilage morphogenesis | 29 | 1 | 0.0199 |
| GO:0022400 | BP | regulation of rhodopsin mediated signaling pathway | 24 | 1 | 0.0199 |
| GO:0046885 | BP | regulation of hormone biosynthetic process | 27 | 1 | 0.0201 |
| GO:1903523 | BP | negative regulation of blood circulation | 36 | 1 | 0.0201 |
| GO:1904353 | BP | regulation of telomere capping | 26 | 1 | 0.0203 |
| GO:0016056 | BP | rhodopsin mediated signaling pathway | 26 | 1 | 0.0205 |
| GO:0033032 | BP | regulation of myeloid cell apoptotic process | 29 | 1 | 0.0210 |
| GO:0060669 | BP | embryonic placenta morphogenesis | 26 | 1 | 0.0210 |
| GO:1905209 | BP | positive regulation of cardiocyte differentiation | 40 | 1 | 0.0218 |
| GO:0048520 | BP | positive regulation of behavior | 28 | 1 | 0.0222 |
| GO:2000310 | BP | regulation of NMDA receptor activity | 33 | 1 | 0.0232 |
| GO:0042730 | BP | fibrinolysis | 28 | 1 | 0.0232 |
| GO:0060562 | BP | epithelial tube morphogenesis | 313 | 2 | 0.0234 |
| GO:0032496 | BP | response to lipopolysaccharide | 317 | 2 | 0.0234 |
| GO:0001782 | BP | B cell homeostasis | 28 | 1 | 0.0234 |
| GO:0071902 | BP | positive regulation of protein serine/threonine kinase activity | 317 | 2 | 0.0238 |
| GO:0010092 | BP | specification of animal organ identity | 32 | 1 | 0.0241 |
| GO:0030224 | BP | monocyte differentiation | 35 | 1 | 0.0241 |
| GO:1903131 | BP | mononuclear cell differentiation | 35 | 1 | 0.0241 |
| GO:0032147 | BP | activation of protein kinase activity | 321 | 2 | 0.0242 |
| GO:0010922 | BP | positive regulation of phosphatase activity | 32 | 1 | 0.0246 |
| GO:0043114 | BP | regulation of vascular permeability | 37 | 1 | 0.0247 |
| GO:0045742 | BP | positive regulation of epidermal growth factor receptor signaling pathway | 31 | 1 | 0.0248 |
| GO:0007603 | BP | phototransduction, visible light | 31 | 1 | 0.0249 |
| GO:0007257 | BP | activation of JUN kinase activity | 37 | 1 | 0.0250 |
| GO:0002237 | BP | response to molecule of bacterial origin | 329 | 2 | 0.0250 |
| GO:0055023 | BP | positive regulation of cardiac muscle tissue growth | 43 | 1 | 0.0251 |
| GO:0034405 | BP | response to fluid shear stress | 36 | 1 | 0.0252 |
| GO:0043405 | BP | regulation of MAP kinase activity | 323 | 2 | 0.0252 |
| GO:0033028 | BP | myeloid cell apoptotic process | 33 | 1 | 0.0257 |
| GO:0032205 | BP | negative regulation of telomere maintenance | 33 | 1 | 0.0257 |
| GO:0051604 | BP | protein maturation | 318 | 2 | 0.0257 |
| GO:1901186 | BP | positive regulation of ERBB signaling pathway | 33 | 1 | 0.0260 |
| GO:0050931 | BP | pigment cell differentiation | 35 | 1 | 0.0265 |
| GO:0008207 | BP | C21-steroid hormone metabolic process | 39 | 1 | 0.0270 |
| GO:0042692 | BP | muscle cell differentiation | 373 | 2 | 0.0271 |
| GO:0060421 | BP | positive regulation of heart growth | 47 | 1 | 0.0276 |
| GO:0007223 | BP | Wnt signaling pathway, calcium modulating pathway | 38 | 1 | 0.0277 |
| GO:0030501 | BP | positive regulation of bone mineralization | 38 | 1 | 0.0279 |
| GO:0001662 | BP | behavioral fear response | 35 | 1 | 0.0280 |
| GO:2000725 | BP | regulation of cardiac muscle cell differentiation | 47 | 1 | 0.0280 |
| GO:0035850 | BP | epithelial cell differentiation involved in kidney development | 41 | 1 | 0.0289 |
| GO:0032350 | BP | regulation of hormone metabolic process | 38 | 1 | 0.0290 |
| GO:0001974 | BP | blood vessel remodeling | 42 | 1 | 0.0291 |
| GO:0002209 | BP | behavioral defense response | 36 | 1 | 0.0292 |
| GO:0043537 | BP | negative regulation of blood vessel endothelial cell migration | 69 | 1 | 0.0292 |
| GO:0007186 | BP | G protein-coupled receptor signaling pathway | 1233 | 3 | 0.0294 |
| GO:0045823 | BP | positive regulation of heart contraction | 38 | 1 | 0.0296 |
| GO:0045745 | BP | positive regulation of G protein-coupled receptor signaling pathway | 31 | 1 | 0.0296 |
| GO:0001709 | BP | cell fate determination | 40 | 1 | 0.0301 |
| GO:0000165 | BP | MAPK cascade | 914 | 3 | 0.0302 |
| GO:0007157 | BP | heterophilic cell-cell adhesion via plasma membrane cell adhesion molecules | 45 | 1 | 0.0308 |
| GO:0042596 | BP | fear response | 38 | 1 | 0.0310 |
| GO:0023014 | BP | signal transduction by protein phosphorylation | 925 | 3 | 0.0310 |
| GO:0030890 | BP | positive regulation of B cell proliferation | 40 | 1 | 0.0315 |
| GO:0048701 | BP | embryonic cranial skeleton morphogenesis | 44 | 1 | 0.0317 |
| GO:0014888 | BP | striated muscle adaptation | 57 | 1 | 0.0322 |
| GO:0050808 | BP | synapse organization | 379 | 2 | 0.0325 |
| GO:0055025 | BP | positive regulation of cardiac muscle tissue development | 57 | 1 | 0.0325 |
| GO:0060043 | BP | regulation of cardiac muscle cell proliferation | 52 | 1 | 0.0325 |
| GO:0070169 | BP | positive regulation of biomineral tissue development | 45 | 1 | 0.0328 |
| GO:0045744 | BP | negative regulation of G protein-coupled receptor signaling pathway | 48 | 1 | 0.0332 |
| GO:0051338 | BP | regulation of transferase activity | 936 | 3 | 0.0333 |
| GO:0048066 | BP | developmental pigmentation | 44 | 1 | 0.0334 |
| GO:0046622 | BP | positive regulation of organ growth | 56 | 1 | 0.0337 |
| GO:0042181 | BP | ketone biosynthetic process | 42 | 1 | 0.0341 |
| GO:0060711 | BP | labyrinthine layer development | 43 | 1 | 0.0343 |
| GO:0061005 | BP | cell differentiation involved in kidney development | 50 | 1 | 0.0350 |
| GO:0001937 | BP | negative regulation of endothelial cell proliferation | 69 | 1 | 0.0351 |
| GO:0000186 | BP | activation of MAPKK activity | 50 | 1 | 0.0352 |
| GO:0048662 | BP | negative regulation of smooth muscle cell proliferation | 61 | 1 | 0.0353 |
| GO:0048641 | BP | regulation of skeletal muscle tissue development | 50 | 1 | 0.0356 |
| GO:0045214 | BP | sarcomere organization | 53 | 1 | 0.0359 |
| GO:0019220 | BP | regulation of phosphate metabolic process | 1734 | 4 | 0.0366 |
| GO:0051174 | BP | regulation of phosphorus metabolic process | 1736 | 4 | 0.0368 |
| GO:0051972 | BP | regulation of telomerase activity | 47 | 1 | 0.0368 |
| GO:0009409 | BP | response to cold | 46 | 1 | 0.0369 |
| GO:0003179 | BP | heart valve morphogenesis | 51 | 1 | 0.0379 |
| GO:0007602 | BP | phototransduction | 52 | 1 | 0.0380 |
| GO:0030449 | BP | regulation of complement activation | 52 | 1 | 0.0384 |
| GO:0031102 | BP | neuron projection regeneration | 56 | 1 | 0.0385 |
| GO:1904705 | BP | regulation of vascular smooth muscle cell proliferation | 76 | 1 | 0.0389 |
| GO:1990874 | BP | vascular smooth muscle cell proliferation | 76 | 1 | 0.0389 |
| GO:2000257 | BP | regulation of protein activation cascade | 53 | 1 | 0.0389 |
| GO:1905207 | BP | regulation of cardiocyte differentiation | 63 | 1 | 0.0389 |
| GO:0019932 | BP | second-messenger-mediated signaling | 424 | 2 | 0.0392 |
| GO:0060038 | BP | cardiac muscle cell proliferation | 62 | 1 | 0.0392 |
| GO:0030195 | BP | negative regulation of blood coagulation | 53 | 1 | 0.0400 |
| GO:1900047 | BP | negative regulation of hemostasis | 54 | 1 | 0.0405 |
| GO:0016233 | BP | telomere capping | 53 | 1 | 0.0411 |
| GO:0009584 | BP | detection of visible light | 54 | 1 | 0.0413 |
| GO:0019229 | BP | regulation of vasoconstriction | 55 | 1 | 0.0414 |
| GO:0050819 | BP | negative regulation of coagulation | 56 | 1 | 0.0414 |
| GO:0048568 | BP | embryonic organ development | 418 | 2 | 0.0416 |
| GO:0045661 | BP | regulation of myoblast differentiation | 52 | 1 | 0.0418 |
| GO:0032835 | BP | glomerulus development | 60 | 1 | 0.0421 |
| GO:0014910 | BP | regulation of smooth muscle cell migration | 76 | 1 | 0.0422 |
| GO:0010596 | BP | negative regulation of endothelial cell migration | 87 | 1 | 0.0422 |
| GO:0050853 | BP | B cell receptor signaling pathway | 59 | 1 | 0.0424 |
| GO:0035306 | BP | positive regulation of dephosphorylation | 56 | 1 | 0.0424 |
| GO:0048645 | BP | animal organ formation | 60 | 1 | 0.0426 |
| GO:0010562 | BP | positive regulation of phosphorus metabolic process | 1086 | 3 | 0.0428 |
| GO:0045937 | BP | positive regulation of phosphate metabolic process | 1086 | 3 | 0.0428 |
| GO:1900449 | BP | regulation of glutamate receptor signaling pathway | 60 | 1 | 0.0428 |
| GO:0003170 | BP | heart valve development | 60 | 1 | 0.0430 |
| GO:0045669 | BP | positive regulation of osteoblast differentiation | 61 | 1 | 0.0431 |
| GO:0051155 | BP | positive regulation of striated muscle cell differentiation | 70 | 1 | 0.0432 |
| GO:0032210 | BP | regulation of telomere maintenance via telomerase | 52 | 1 | 0.0435 |
| GO:0051965 | BP | positive regulation of synapse assembly | 61 | 1 | 0.0435 |
| GO:0097755 | BP | positive regulation of blood vessel diameter | 57 | 1 | 0.0436 |
| GO:0050878 | BP | regulation of body fluid levels | 478 | 2 | 0.0443 |
| GO:0023061 | BP | signal release | 444 | 2 | 0.0445 |
| GO:0030888 | BP | regulation of B cell proliferation | 59 | 1 | 0.0449 |
| GO:0002260 | BP | lymphocyte homeostasis | 57 | 1 | 0.0453 |
| GO:0055021 | BP | regulation of cardiac muscle tissue growth | 72 | 1 | 0.0463 |
| GO:0014909 | BP | smooth muscle cell migration | 83 | 1 | 0.0472 |
| GO:1904356 | BP | regulation of telomere maintenance via telomere lengthening | 58 | 1 | 0.0477 |
| GO:0060350 | BP | endochondral bone morphogenesis | 72 | 1 | 0.0480 |
| GO:1904888 | BP | cranial skeletal system development | 65 | 1 | 0.0481 |
| GO:0060998 | BP | regulation of dendritic spine development | 68 | 1 | 0.0482 |
| GO:0099601 | BP | regulation of neurotransmitter receptor activity | 69 | 1 | 0.0483 |
| GO:0016311 | BP | dephosphorylation | 460 | 2 | 0.0485 |
| GO:0009583 | BP | detection of light stimulus | 66 | 1 | 0.0487 |
| GO:0035914 | BP | skeletal muscle cell differentiation | 66 | 1 | 0.0493 |
| GO:0014855 | BP | striated muscle cell proliferation | 76 | 1 | 0.0495 |
| GO:0051966 | BP | regulation of synaptic transmission, glutamatergic | 68 | 1 | 0.0495 |
| GO:0030500 | BP | regulation of bone mineralization | 71 | 1 | 0.0497 |
| GO:0030239 | BP | myofibril assembly | 73 | 1 | 0.0497 |

(B)

|  | **Description** | **N** | **DE** | **P.DE** |
| --- | --- | --- | --- | --- |
| path:hsa04010 | MAPK signaling pathway | 283 | 2 | 0.0177 |
| path:hsa04744 | Phototransduction | 27 | 1 | 0.0208 |

(C)

|  | **ONTOLOGY** | **TERM** | **N** | **DE** | **P.DE** |
| --- | --- | --- | --- | --- | --- |
| GO:0021859 | BP | pyramidal neuron differentiation | 6 | 1 | 0.0019 |
| GO:0055064 | BP | chloride ion homeostasis | 14 | 1 | 0.0029 |
| GO:0055083 | BP | monovalent inorganic anion homeostasis | 26 | 1 | 0.0044 |
| GO:0038007 | BP | netrin-activated signaling pathway | 13 | 1 | 0.0046 |
| GO:0055075 | BP | potassium ion homeostasis | 29 | 1 | 0.0052 |
| GO:0006120 | BP | mitochondrial electron transport, NADH to ubiquinone | 46 | 1 | 0.0056 |
| GO:0006884 | BP | cell volume homeostasis | 27 | 1 | 0.0064 |
| GO:0010257 | BP | NADH dehydrogenase complex assembly | 55 | 1 | 0.0067 |
| GO:0032981 | BP | mitochondrial respiratory chain complex I assembly | 55 | 1 | 0.0067 |
| GO:1990573 | BP | potassium ion import across plasma membrane | 43 | 1 | 0.0073 |
| GO:0055081 | BP | anion homeostasis | 56 | 1 | 0.0084 |
| GO:0010107 | BP | potassium ion import | 47 | 1 | 0.0088 |
| GO:0042775 | BP | mitochondrial ATP synthesis coupled electron transport | 79 | 1 | 0.0097 |
| GO:0042773 | BP | ATP synthesis coupled electron transport | 80 | 1 | 0.0097 |
| GO:0033108 | BP | mitochondrial respiratory chain complex assembly | 87 | 1 | 0.0105 |
| GO:2001222 | BP | regulation of neuron migration | 39 | 1 | 0.0110 |
| GO:0022904 | BP | respiratory electron transport chain | 99 | 1 | 0.0130 |
| GO:0006119 | BP | oxidative phosphorylation | 121 | 1 | 0.0138 |
| GO:1902476 | BP | chloride transmembrane transport | 80 | 1 | 0.0141 |
| GO:0098659 | BP | inorganic cation import across plasma membrane | 80 | 1 | 0.0160 |
| GO:0099587 | BP | inorganic ion import across plasma membrane | 80 | 1 | 0.0160 |
| GO:0006821 | BP | chloride transport | 99 | 1 | 0.0169 |
| GO:0098661 | BP | inorganic anion transmembrane transport | 102 | 1 | 0.0181 |
| GO:0098739 | BP | import across plasma membrane | 106 | 1 | 0.0206 |
| GO:0022900 | BP | electron transport chain | 165 | 1 | 0.0220 |
| GO:0045333 | BP | cellular respiration | 172 | 1 | 0.0224 |
| GO:0055067 | BP | monovalent inorganic cation homeostasis | 147 | 1 | 0.0243 |
| GO:0015698 | BP | inorganic anion transport | 158 | 1 | 0.0264 |
| GO:0001764 | BP | neuron migration | 148 | 1 | 0.0367 |
| GO:0015980 | BP | energy derivation by oxidation of organic compounds | 259 | 1 | 0.0369 |
| GO:0046034 | BP | ATP metabolic process | 277 | 1 | 0.0393 |
| GO:0008361 | BP | regulation of cell size | 169 | 1 | 0.0410 |
| GO:0021953 | BP | central nervous system neuron differentiation | 177 | 1 | 0.0414 |
| GO:0071804 | BP | cellular potassium ion transport | 209 | 1 | 0.0420 |
| GO:0071805 | BP | potassium ion transmembrane transport | 209 | 1 | 0.0420 |
| GO:0009205 | BP | purine ribonucleoside triphosphate metabolic process | 306 | 1 | 0.0422 |
| GO:0009144 | BP | purine nucleoside triphosphate metabolic process | 313 | 1 | 0.0429 |
| GO:0009199 | BP | ribonucleoside triphosphate metabolic process | 311 | 1 | 0.0433 |
| GO:0009167 | BP | purine ribonucleoside monophosphate metabolic process | 307 | 1 | 0.0448 |
| GO:0009126 | BP | purine nucleoside monophosphate metabolic process | 308 | 1 | 0.0450 |
| GO:0009141 | BP | nucleoside triphosphate metabolic process | 332 | 1 | 0.0453 |
| GO:0006813 | BP | potassium ion transport | 232 | 1 | 0.0459 |
| GO:0009161 | BP | ribonucleoside monophosphate metabolic process | 318 | 1 | 0.0464 |
| GO:0009123 | BP | nucleoside monophosphate metabolic process | 339 | 1 | 0.0485 |
| GO:0098656 | BP | anion transmembrane transport | 267 | 1 | 0.0488 |
| GO:0016043 | BP | cellular component organization | 6082 | 3 | 0.0493 |

(D)

|  | **Description** | **N** | **DE** | **P.DE** |
| --- | --- | --- | --- | --- |
| path:hsa00190 | Oxidative phosphorylation | 117 | 1 | 0.0143 |
| path:hsa04932 | Non-alcoholic fatty liver disease | 148 | 1 | 0.0243 |
| path:hsa05208 | Chemical carcinogenesis - reactive oxygen species | 202 | 1 | 0.0290 |
| path:hsa04723 | Retrograde endocannabinoid signaling | 134 | 1 | 0.0296 |
| path:hsa05415 | Diabetic cardiomyopathy | 180 | 1 | 0.0298 |
| path:hsa05012 | Parkinson disease | 245 | 1 | 0.0367 |
| path:hsa05020 | Prion disease | 253 | 1 | 0.0377 |
| path:hsa04714 | Thermogenesis | 213 | 1 | 0.0377 |
| path:hsa05016 | Huntington disease | 286 | 1 | 0.0451 |
| path:hsa04360 | Axon guidance | 175 | 1 | 0.0488 |
| path:hsa05014 | Amyotrophic lateral sclerosis | 341 | 1 | 0.0489 |

**Supplementary Table 9**. Gene set enrichment analysis result using female-specific significant DMRs associated with DEHP concentration in maternal urine samples during late pregnancy (A: GO terms, B: KEGG pathways) and birth urine samples (C: GO terms, D: KEGG pathways).

(A)

|  | **ONTOLOGY** | **TERM** | **N** | **DE** | **P.DE** |
| --- | --- | --- | --- | --- | --- |
| GO:0022411 | BP | cellular component disassembly | 527 | 3 | 0.0019 |
| GO:0030263 | BP | apoptotic chromosome condensation | 5 | 1 | 0.0027 |
| GO:1901490 | BP | regulation of lymphangiogenesis | 5 | 1 | 0.0028 |
| GO:1901098 | BP | positive regulation of autophagosome maturation | 7 | 1 | 0.0036 |
| GO:1901096 | BP | regulation of autophagosome maturation | 10 | 1 | 0.0050 |
| GO:0045657 | BP | positive regulation of monocyte differentiation | 10 | 1 | 0.0052 |
| GO:0051657 | BP | maintenance of organelle location | 11 | 1 | 0.0059 |
| GO:0051645 | BP | Golgi localization | 14 | 1 | 0.0079 |
| GO:0001946 | BP | lymphangiogenesis | 16 | 1 | 0.0089 |
| GO:0060716 | BP | labyrinthine layer blood vessel development | 18 | 1 | 0.0090 |
| GO:0035493 | BP | SNARE complex assembly | 17 | 1 | 0.0093 |
| GO:0032984 | BP | protein-containing complex disassembly | 319 | 2 | 0.0101 |
| GO:0045655 | BP | regulation of monocyte differentiation | 20 | 1 | 0.0105 |
| GO:0036303 | BP | lymph vessel morphogenesis | 19 | 1 | 0.0108 |
| GO:0001945 | BP | lymph vessel development | 25 | 1 | 0.0147 |
| GO:0060674 | BP | placenta blood vessel development | 31 | 1 | 0.0148 |
| GO:0071985 | BP | multivesicular body sorting pathway | 32 | 1 | 0.0152 |
| GO:0030262 | BP | apoptotic nuclear changes | 34 | 1 | 0.0165 |
| GO:0097352 | BP | autophagosome maturation | 36 | 1 | 0.0171 |
| GO:0006921 | BP | cellular component disassembly involved in execution phase of apoptosis | 36 | 1 | 0.0175 |
| GO:0043243 | BP | positive regulation of protein complex disassembly | 36 | 1 | 0.0178 |
| GO:0032801 | BP | receptor catabolic process | 34 | 1 | 0.0183 |
| GO:0030224 | BP | monocyte differentiation | 35 | 1 | 0.0187 |
| GO:1903131 | BP | mononuclear cell differentiation | 35 | 1 | 0.0187 |
| GO:0015701 | BP | bicarbonate transport | 41 | 1 | 0.0195 |
| GO:0030261 | BP | chromosome condensation | 46 | 1 | 0.0223 |
| GO:0060711 | BP | labyrinthine layer development | 43 | 1 | 0.0228 |
| GO:2000772 | BP | regulation of cellular senescence | 50 | 1 | 0.0238 |
| GO:0002763 | BP | positive regulation of myeloid leukocyte differentiation | 53 | 1 | 0.0264 |
| GO:0043537 | BP | negative regulation of blood vessel endothelial cell migration | 69 | 1 | 0.0265 |
| GO:0001937 | BP | negative regulation of endothelial cell proliferation | 69 | 1 | 0.0273 |
| GO:0090342 | BP | regulation of cell aging | 58 | 1 | 0.0277 |
| GO:0016239 | BP | positive regulation of macroautophagy | 65 | 1 | 0.0334 |
| GO:0010596 | BP | negative regulation of endothelial cell migration | 87 | 1 | 0.0356 |
| GO:0090398 | BP | cellular senescence | 74 | 1 | 0.0359 |
| GO:0070126 | BP | mitochondrial translational termination | 89 | 1 | 0.0374 |
| GO:0070125 | BP | mitochondrial translational elongation | 88 | 1 | 0.0374 |
| GO:0097194 | BP | execution phase of apoptosis | 80 | 1 | 0.0380 |
| GO:0006303 | BP | double-strand break repair via nonhomologous end joining | 80 | 1 | 0.0386 |
| GO:0032465 | BP | regulation of cytokinesis | 83 | 1 | 0.0399 |
| GO:0006890 | BP | retrograde vesicle-mediated transport, Golgi to ER | 84 | 1 | 0.0402 |
| GO:0000726 | BP | non-recombinational repair | 86 | 1 | 0.0417 |
| GO:0006415 | BP | translational termination | 102 | 1 | 0.0435 |
| GO:0045639 | BP | positive regulation of myeloid cell differentiation | 86 | 1 | 0.0437 |
| GO:0001892 | BP | embryonic placenta development | 83 | 1 | 0.0438 |
| GO:0010633 | BP | negative regulation of epithelial cell migration | 104 | 1 | 0.0451 |
| GO:0051651 | BP | maintenance of location in cell | 88 | 1 | 0.0455 |
| GO:0006906 | BP | vesicle fusion | 91 | 1 | 0.0477 |
| GO:0090174 | BP | organelle membrane fusion | 97 | 1 | 0.0500 |

(B)

|  | **Description** | **N** | **DE** | **P.DE** |
| --- | --- | --- | --- | --- |
| path:hsa00520 | Amino sugar and nucleotide sugar metabolism | 48 | 1 | 0.0230 |
| path:hsa00270 | Cysteine and methionine metabolism | 49 | 1 | 0.0233 |
| path:hsa01232 | Nucleotide metabolism | 81 | 1 | 0.0396 |
| path:hsa03015 | mRNA surveillance pathway | 85 | 1 | 0.0415 |
| path:hsa03013 | Nucleocytoplasmic transport | 101 | 1 | 0.0474 |

(C)

|  | **ONTOLOGY** | **TERM** | **N** | **DE** | **P.DE** |
| --- | --- | --- | --- | --- | --- |
| GO:1905954 | BP | positive regulation of lipid localization | 82 | 2 | 0.0013 |
| GO:1905952 | BP | regulation of lipid localization | 155 | 2 | 0.0040 |
| GO:0031999 | BP | negative regulation of fatty acid beta-oxidation | 5 | 1 | 0.0059 |
| GO:0035627 | BP | ceramide transport | 13 | 1 | 0.0062 |
| GO:0006853 | BP | carnitine shuttle | 10 | 1 | 0.0077 |
| GO:0042362 | BP | fat-soluble vitamin biosynthetic process | 15 | 1 | 0.0079 |
| GO:0034375 | BP | high-density lipoprotein particle remodeling | 18 | 1 | 0.0087 |
| GO:0046322 | BP | negative regulation of fatty acid oxidation | 10 | 1 | 0.0088 |
| GO:0018230 | BP | peptidyl-L-cysteine S-palmitoylation | 19 | 1 | 0.0105 |
| GO:0018231 | BP | peptidyl-S-diacylglycerol-L-cysteine  biosynthetic process from peptidyl-cysteine | 19 | 1 | 0.0105 |
| GO:0043086 | BP | negative regulation of catalytic activity | 777 | 3 | 0.0112 |
| GO:0009110 | BP | vitamin biosynthetic process | 23 | 1 | 0.0117 |
| GO:1901617 | BP | organic hydroxy compound biosynthetic process | 264 | 2 | 0.0119 |
| GO:0034368 | BP | protein-lipid complex remodeling | 28 | 1 | 0.0122 |
| GO:0034369 | BP | plasma lipoprotein particle remodeling | 28 | 1 | 0.0122 |
| GO:0010875 | BP | positive regulation of cholesterol efflux | 16 | 1 | 0.0124 |
| GO:0034367 | BP | protein-containing complex remodeling | 29 | 1 | 0.0124 |
| GO:0061158 | BP | 3'-UTR-mediated mRNA destabilization | 16 | 1 | 0.0124 |
| GO:1902001 | BP | fatty acid transmembrane transport | 15 | 1 | 0.0137 |
| GO:0050995 | BP | negative regulation of lipid catabolic process | 23 | 1 | 0.0147 |
| GO:0014850 | BP | response to muscle activity | 24 | 1 | 0.0149 |
| GO:0045922 | BP | negative regulation of fatty acid metabolic process | 31 | 1 | 0.0151 |
| GO:0010884 | BP | positive regulation of lipid storage | 22 | 1 | 0.0152 |
| GO:0035278 | BP | miRNA mediated inhibition of translation | 86 | 1 | 0.0153 |
| GO:0040033 | BP | negative regulation of translation, ncRNA-mediated | 86 | 1 | 0.0153 |
| GO:0045974 | BP | regulation of translation, ncRNA-mediated | 86 | 1 | 0.0153 |
| GO:0018345 | BP | protein palmitoylation | 31 | 1 | 0.0165 |
| GO:0032373 | BP | positive regulation of sterol transport | 23 | 1 | 0.0168 |
| GO:0032376 | BP | positive regulation of cholesterol transport | 23 | 1 | 0.0168 |
| GO:0010874 | BP | regulation of cholesterol efflux | 38 | 1 | 0.0170 |
| GO:0000462 | BP | maturation of SSU-rRNA from tricistronic rRNA transcript  (SSU-rRNA, 5.8S rRNA, LSU-rRNA) | 33 | 1 | 0.0173 |
| GO:0031998 | BP | regulation of fatty acid beta-oxidation | 17 | 1 | 0.0173 |
| GO:0030490 | BP | maturation of SSU-rRNA | 44 | 1 | 0.0204 |
| GO:2000637 | BP | positive regulation of gene silencing by miRNA | 22 | 1 | 0.0205 |
| GO:0010586 | BP | miRNA metabolic process | 30 | 1 | 0.0205 |
| GO:0006869 | BP | lipid transport | 357 | 2 | 0.0209 |
| GO:0060148 | BP | positive regulation of posttranscriptional gene silencing | 23 | 1 | 0.0210 |
| GO:0006084 | BP | acetyl-CoA metabolic process | 36 | 1 | 0.0217 |
| GO:0006482 | BP | protein demethylation | 26 | 1 | 0.0222 |
| GO:0008214 | BP | protein dealkylation | 26 | 1 | 0.0222 |
| GO:0061157 | BP | mRNA destabilization | 30 | 1 | 0.0225 |
| GO:0071827 | BP | plasma lipoprotein particle organization | 45 | 1 | 0.0240 |
| GO:0033344 | BP | cholesterol efflux | 56 | 1 | 0.0246 |
| GO:0050779 | BP | RNA destabilization | 32 | 1 | 0.0250 |
| GO:0010876 | BP | lipid localization | 392 | 2 | 0.0253 |
| GO:0018198 | BP | peptidyl-cysteine modification | 43 | 1 | 0.0254 |
| GO:0006470 | BP | protein dephosphorylation | 309 | 2 | 0.0254 |
| GO:0006775 | BP | fat-soluble vitamin metabolic process | 43 | 1 | 0.0256 |
| GO:0046320 | BP | regulation of fatty acid oxidation | 29 | 1 | 0.0260 |
| GO:0034660 | BP | ncRNA metabolic process | 535 | 2 | 0.0262 |
| GO:0042274 | BP | ribosomal small subunit biogenesis | 64 | 1 | 0.0266 |
| GO:0051346 | BP | negative regulation of hydrolase activity | 437 | 2 | 0.0270 |
| GO:0032365 | BP | intracellular lipid transport | 42 | 1 | 0.0278 |
| GO:0071825 | BP | protein-lipid complex subunit organization | 49 | 1 | 0.0287 |
| GO:0044092 | BP | negative regulation of molecular function | 1095 | 3 | 0.0288 |
| GO:0032371 | BP | regulation of sterol transport | 62 | 1 | 0.0295 |
| GO:0032374 | BP | regulation of cholesterol transport | 62 | 1 | 0.0295 |
| GO:0097009 | BP | energy homeostasis | 34 | 1 | 0.0305 |
| GO:0045540 | BP | regulation of cholesterol biosynthetic process | 49 | 1 | 0.0333 |
| GO:0106118 | BP | regulation of sterol biosynthetic process | 49 | 1 | 0.0333 |
| GO:0061014 | BP | positive regulation of mRNA catabolic process | 48 | 1 | 0.0360 |
| GO:0050994 | BP | regulation of lipid catabolic process | 53 | 1 | 0.0360 |
| GO:0034260 | BP | negative regulation of GTPase activity | 44 | 1 | 0.0361 |
| GO:0010883 | BP | regulation of lipid storage | 46 | 1 | 0.0363 |
| GO:0035196 | BP | production of miRNAs involved in gene silencing by miRNA | 46 | 1 | 0.0372 |
| GO:0015909 | BP | long-chain fatty acid transport | 67 | 1 | 0.0372 |
| GO:0060421 | BP | positive regulation of heart growth | 47 | 1 | 0.0383 |
| GO:0032370 | BP | positive regulation of lipid transport | 61 | 1 | 0.0387 |
| GO:0031050 | BP | dsRNA processing | 51 | 1 | 0.0394 |
| GO:0070918 | BP | production of small RNA involved in gene silencing by RNA | 51 | 1 | 0.0394 |
| GO:0035384 | BP | thioester biosynthetic process | 50 | 1 | 0.0398 |
| GO:0071616 | BP | acyl-CoA biosynthetic process | 50 | 1 | 0.0398 |
| GO:0070988 | BP | demethylation | 64 | 1 | 0.0398 |
| GO:0032515 | BP | negative regulation of phosphoprotein phosphatase activity | 58 | 1 | 0.0400 |
| GO:0015711 | BP | organic anion transport | 462 | 2 | 0.0401 |
| GO:1990542 | BP | mitochondrial transmembrane transport | 95 | 1 | 0.0411 |
| GO:0090181 | BP | regulation of cholesterol metabolic process | 62 | 1 | 0.0417 |
| GO:1901615 | BP | organic hydroxy compound metabolic process | 514 | 2 | 0.0417 |
| GO:0006695 | BP | cholesterol biosynthetic process | 71 | 1 | 0.0425 |
| GO:1902653 | BP | secondary alcohol biosynthetic process | 72 | 1 | 0.0427 |
| GO:0014823 | BP | response to activity | 65 | 1 | 0.0431 |
| GO:0030317 | BP | flagellated sperm motility | 82 | 1 | 0.0437 |
| GO:0097722 | BP | sperm motility | 82 | 1 | 0.0437 |
| GO:0006497 | BP | protein lipidation | 83 | 1 | 0.0438 |
| GO:0046622 | BP | positive regulation of organ growth | 56 | 1 | 0.0442 |
| GO:0035308 | BP | negative regulation of protein dephosphorylation | 63 | 1 | 0.0451 |
| GO:0042158 | BP | lipoprotein biosynthetic process | 88 | 1 | 0.0454 |
| GO:0016126 | BP | sterol biosynthetic process | 77 | 1 | 0.0460 |
| GO:0006635 | BP | fatty acid beta-oxidation | 71 | 1 | 0.0460 |
| GO:0051865 | BP | protein autoubiquitination | 67 | 1 | 0.0487 |
| GO:0019915 | BP | lipid storage | 68 | 1 | 0.0489 |
| GO:0016311 | BP | dephosphorylation | 460 | 2 | 0.0490 |
| GO:0006919 | BP | activation of cysteine-type endopeptidase activity involved in apoptotic process | 84 | 1 | 0.0495 |

(D)

|  | **Description** | **N** | **DE** | **P.DE** |
| --- | --- | --- | --- | --- |
| path:hsa00061 | Fatty acid biosynthesis | 16 | 1 | 0.0164 |
| path:hsa00640 | Propanoate metabolism | 32 | 1 | 0.0221 |
| path:hsa00620 | Pyruvate metabolism | 46 | 1 | 0.0231 |
| path:hsa04940 | Type I diabetes mellitus | 41 | 1 | 0.0238 |
| path:hsa04979 | Cholesterol metabolism | 51 | 1 | 0.0314 |
| path:hsa03320 | PPAR signaling pathway | 72 | 1 | 0.0429 |

**Supplementary Table 10**. Gene set enrichment analysis result using male-specific significant DMRs associated with MEOHP concentration in maternal urine samples during late pregnancy (A: GO terms, B: KEGG pathways) and birth urine samples (C: GO terms, D: KEGG pathways).

(A)

|  | **ONTOLOGY** | **TERM** | **N** | **DE** | **P.DE** |
| --- | --- | --- | --- | --- | --- |
| GO:0045918 | BP | negative regulation of cytolysis | 5 | 1 | 0.0016 |
| GO:0045347 | BP | negative regulation of MHC class II biosynthetic process | 5 | 1 | 0.0023 |
| GO:0019852 | BP | L-ascorbic acid metabolic process | 10 | 1 | 0.0036 |
| GO:0060316 | BP | positive regulation of ryanodine-sensitive calcium-release channel activity | 8 | 1 | 0.0038 |
| GO:0060315 | BP | negative regulation of ryanodine-sensitive calcium-release channel activity | 10 | 1 | 0.0049 |
| GO:0051852 | BP | disruption by host of symbiont cells | 12 | 1 | 0.0050 |
| GO:0051873 | BP | killing by host of symbiont cells | 12 | 1 | 0.0050 |
| GO:0042178 | BP | xenobiotic catabolic process | 13 | 1 | 0.0050 |
| GO:0045653 | BP | negative regulation of megakaryocyte differentiation | 16 | 1 | 0.0053 |
| GO:0051280 | BP | negative regulation of release of sequestered calcium ion into cytosol | 14 | 1 | 0.0056 |
| GO:0014819 | BP | regulation of skeletal muscle contraction | 12 | 1 | 0.0057 |
| GO:0045989 | BP | positive regulation of striated muscle contraction | 15 | 1 | 0.0057 |
| GO:0051818 | BP | disruption of cells of other organism involved in symbiotic interaction | 15 | 1 | 0.0058 |
| GO:0051883 | BP | killing of cells in other organism involved in symbiotic interaction | 15 | 1 | 0.0058 |
| GO:0045346 | BP | regulation of MHC class II biosynthetic process | 14 | 1 | 0.0058 |
| GO:0042268 | BP | regulation of cytolysis | 17 | 1 | 0.0060 |
| GO:0045342 | BP | MHC class II biosynthetic process | 15 | 1 | 0.0062 |
| GO:0045651 | BP | positive regulation of macrophage differentiation | 14 | 1 | 0.0063 |
| GO:0051284 | BP | positive regulation of sequestering of calcium ion | 16 | 1 | 0.0063 |
| GO:0010523 | BP | negative regulation of calcium ion transport into cytosol | 18 | 1 | 0.0069 |
| GO:0010744 | BP | positive regulation of macrophage derived foam cell differentiation | 17 | 1 | 0.0070 |
| GO:0010881 | BP | regulation of cardiac muscle contraction by regulation of the release of sequestered calcium ion | 22 | 1 | 0.0078 |
| GO:1901685 | BP | glutathione derivative metabolic process | 22 | 1 | 0.0080 |
| GO:1901687 | BP | glutathione derivative biosynthetic process | 22 | 1 | 0.0080 |
| GO:0042832 | BP | defense response to protozoan | 22 | 1 | 0.0087 |
| GO:0045332 | BP | phospholipid translocation | 24 | 1 | 0.0088 |
| GO:2000738 | BP | positive regulation of stem cell differentiation | 21 | 1 | 0.0089 |
| GO:0071243 | BP | cellular response to arsenic-containing substance | 18 | 1 | 0.0090 |
| GO:0001562 | BP | response to protozoan | 23 | 1 | 0.0090 |
| GO:0045649 | BP | regulation of macrophage differentiation | 21 | 1 | 0.0090 |
| GO:0034204 | BP | lipid translocation | 25 | 1 | 0.0093 |
| GO:0032259 | BP | methylation | 347 | 2 | 0.0097 |
| GO:0010882 | BP | regulation of cardiac muscle contraction by calcium ion signaling | 27 | 1 | 0.0098 |
| GO:0060314 | BP | regulation of ryanodine-sensitive calcium-release channel activity | 24 | 1 | 0.0099 |
| GO:0019932 | BP | second-messenger-mediated signaling | 424 | 2 | 0.0113 |
| GO:0010880 | BP | regulation of release of sequestered calcium ion into cytosol by sarcoplasmic reticulum | 30 | 1 | 0.0115 |
| GO:0050849 | BP | negative regulation of calcium-mediated signaling | 30 | 1 | 0.0123 |
| GO:0014808 | BP | release of sequestered calcium ion into cytosol by sarcoplasmic reticulum | 34 | 1 | 0.0128 |
| GO:0010743 | BP | regulation of macrophage derived foam cell differentiation | 30 | 1 | 0.0128 |
| GO:1903514 | BP | release of sequestered calcium ion into cytosol by endoplasmic reticulum | 35 | 1 | 0.0130 |
| GO:0019835 | BP | cytolysis | 40 | 1 | 0.0136 |
| GO:1901020 | BP | negative regulation of calcium ion transmembrane transporter activity | 34 | 1 | 0.0142 |
| GO:0046685 | BP | response to arsenic-containing substance | 31 | 1 | 0.0147 |
| GO:0070296 | BP | sarcoplasmic reticulum calcium ion transport | 40 | 1 | 0.0148 |
| GO:1903170 | BP | negative regulation of calcium ion transmembrane transport | 39 | 1 | 0.0155 |
| GO:1901099 | BP | negative regulation of signal transduction in absence of ligand | 35 | 1 | 0.0157 |
| GO:2001240 | BP | negative regulation of extrinsic apoptotic signaling pathway in absence of ligand | 35 | 1 | 0.0157 |
| GO:0010742 | BP | macrophage derived foam cell differentiation | 36 | 1 | 0.0160 |
| GO:0090077 | BP | foam cell differentiation | 36 | 1 | 0.0160 |
| GO:0001525 | BP | angiogenesis | 563 | 2 | 0.0161 |
| GO:1901021 | BP | positive regulation of calcium ion transmembrane transporter activity | 37 | 1 | 0.0162 |
| GO:0050850 | BP | positive regulation of calcium-mediated signaling | 43 | 1 | 0.0163 |
| GO:0051281 | BP | positive regulation of release of sequestered calcium ion into cytosol | 40 | 1 | 0.0166 |
| GO:0031640 | BP | killing of cells of other organism | 57 | 1 | 0.0172 |
| GO:0044364 | BP | disruption of cells of other organism | 57 | 1 | 0.0172 |
| GO:2001258 | BP | negative regulation of cation channel activity | 47 | 1 | 0.0173 |
| GO:0030225 | BP | macrophage differentiation | 42 | 1 | 0.0173 |
| GO:0003009 | BP | skeletal muscle contraction | 41 | 1 | 0.0179 |
| GO:0061844 | BP | antimicrobial humoral immune response mediated by antimicrobial peptide | 61 | 1 | 0.0180 |
| GO:0035722 | BP | interleukin-12-mediated signaling pathway | 44 | 1 | 0.0195 |
| GO:2001239 | BP | regulation of extrinsic apoptotic signaling pathway in absence of ligand | 45 | 1 | 0.0195 |
| GO:0097035 | BP | regulation of membrane lipid distribution | 49 | 1 | 0.0198 |
| GO:0071349 | BP | cellular response to interleukin-12 | 46 | 1 | 0.0202 |
| GO:0045933 | BP | positive regulation of muscle contraction | 47 | 1 | 0.0203 |
| GO:0070671 | BP | response to interleukin-12 | 47 | 1 | 0.0209 |
| GO:0048514 | BP | blood vessel morphogenesis | 656 | 2 | 0.0224 |
| GO:0010524 | BP | positive regulation of calcium ion transport into cytosol | 54 | 1 | 0.0227 |
| GO:0002763 | BP | positive regulation of myeloid leukocyte differentiation | 53 | 1 | 0.0229 |
| GO:0051926 | BP | negative regulation of calcium ion transport | 63 | 1 | 0.0233 |
| GO:0050879 | BP | multicellular organismal movement | 54 | 1 | 0.0236 |
| GO:0050881 | BP | musculoskeletal movement | 54 | 1 | 0.0236 |
| GO:0070098 | BP | chemokine-mediated signaling pathway | 84 | 1 | 0.0258 |
| GO:0032413 | BP | negative regulation of ion transmembrane transporter activity | 73 | 1 | 0.0268 |
| GO:0045652 | BP | regulation of megakaryocyte differentiation | 73 | 1 | 0.0272 |
| GO:2001259 | BP | positive regulation of cation channel activity | 69 | 1 | 0.0272 |
| GO:0015914 | BP | phospholipid transport | 74 | 1 | 0.0281 |
| GO:0001568 | BP | blood vessel development | 732 | 2 | 0.0282 |
| GO:0006305 | BP | DNA alkylation | 70 | 1 | 0.0283 |
| GO:0006306 | BP | DNA methylation | 70 | 1 | 0.0283 |
| GO:1904427 | BP | positive regulation of calcium ion transmembrane transport | 69 | 1 | 0.0284 |
| GO:1990868 | BP | response to chemokine | 93 | 1 | 0.0288 |
| GO:1990869 | BP | cellular response to chemokine | 93 | 1 | 0.0288 |
| GO:0051851 | BP | modification by host of symbiont morphology or physiology | 71 | 1 | 0.0294 |
| GO:0032410 | BP | negative regulation of transporter activity | 84 | 1 | 0.0297 |
| GO:0055117 | BP | regulation of cardiac muscle contraction | 77 | 1 | 0.0297 |
| GO:0038034 | BP | signal transduction in absence of ligand | 70 | 1 | 0.0299 |
| GO:0097192 | BP | extrinsic apoptotic signaling pathway in absence of ligand | 70 | 1 | 0.0299 |
| GO:0019730 | BP | antimicrobial humoral response | 103 | 1 | 0.0301 |
| GO:0001944 | BP | vasculature development | 761 | 2 | 0.0304 |
| GO:0051279 | BP | regulation of release of sequestered calcium ion into cytosol | 80 | 1 | 0.0309 |
| GO:0072358 | BP | cardiovascular system development | 769 | 2 | 0.0310 |
| GO:0051702 | BP | interaction with symbiont | 76 | 1 | 0.0316 |
| GO:1904063 | BP | negative regulation of cation transmembrane transport | 86 | 1 | 0.0322 |
| GO:0030593 | BP | neutrophil chemotaxis | 98 | 1 | 0.0327 |
| GO:0045638 | BP | negative regulation of myeloid cell differentiation | 88 | 1 | 0.0328 |
| GO:0032760 | BP | positive regulation of tumor necrosis factor production | 84 | 1 | 0.0331 |
| GO:0030219 | BP | megakaryocyte differentiation | 90 | 1 | 0.0335 |
| GO:1903557 | BP | positive regulation of tumor necrosis factor superfamily cytokine production | 86 | 1 | 0.0338 |
| GO:0044728 | BP | DNA methylation or demethylation | 91 | 1 | 0.0342 |
| GO:0019221 | BP | cytokine-mediated signaling pathway | 755 | 2 | 0.0344 |
| GO:1901019 | BP | regulation of calcium ion transmembrane transporter activity | 84 | 1 | 0.0347 |
| GO:0006942 | BP | regulation of striated muscle contraction | 91 | 1 | 0.0356 |
| GO:0034766 | BP | negative regulation of ion transmembrane transport | 97 | 1 | 0.0364 |
| GO:0060218 | BP | hematopoietic stem cell differentiation | 81 | 1 | 0.0365 |
| GO:0050848 | BP | regulation of calcium-mediated signaling | 94 | 1 | 0.0366 |
| GO:0006767 | BP | water-soluble vitamin metabolic process | 84 | 1 | 0.0368 |
| GO:0045055 | BP | regulated exocytosis | 756 | 2 | 0.0371 |
| GO:0045639 | BP | positive regulation of myeloid cell differentiation | 86 | 1 | 0.0377 |
| GO:1990266 | BP | neutrophil migration | 109 | 1 | 0.0379 |
| GO:0010522 | BP | regulation of calcium ion transport into cytosol | 100 | 1 | 0.0391 |
| GO:0071621 | BP | granulocyte chemotaxis | 117 | 1 | 0.0393 |
| GO:0098869 | BP | cellular oxidant detoxification | 97 | 1 | 0.0395 |
| GO:0032414 | BP | positive regulation of ion transmembrane transporter activity | 99 | 1 | 0.0399 |
| GO:0015748 | BP | organophosphate ester transport | 100 | 1 | 0.0405 |
| GO:0006805 | BP | xenobiotic metabolic process | 124 | 1 | 0.0415 |
| GO:0035239 | BP | tube morphogenesis | 887 | 2 | 0.0421 |
| GO:2001237 | BP | negative regulation of extrinsic apoptotic signaling pathway | 100 | 1 | 0.0428 |
| GO:0032411 | BP | positive regulation of transporter activity | 107 | 1 | 0.0432 |
| GO:0051817 | BP | modification of morphology or physiology of other organism involved in symbiotic interaction | 106 | 1 | 0.0432 |
| GO:1990748 | BP | cellular detoxification | 107 | 1 | 0.0434 |
| GO:0016525 | BP | negative regulation of angiogenesis | 158 | 1 | 0.0444 |
| GO:0006304 | BP | DNA modification | 113 | 1 | 0.0444 |
| GO:0051209 | BP | release of sequestered calcium ion into cytosol | 121 | 1 | 0.0452 |
| GO:2000181 | BP | negative regulation of blood vessel morphogenesis | 160 | 1 | 0.0453 |
| GO:0034763 | BP | negative regulation of transmembrane transport | 122 | 1 | 0.0455 |
| GO:0002761 | BP | regulation of myeloid leukocyte differentiation | 114 | 1 | 0.0456 |
| GO:0051283 | BP | negative regulation of sequestering of calcium ion | 123 | 1 | 0.0459 |
| GO:0030097 | BP | hemopoiesis | 854 | 2 | 0.0460 |
| GO:0051928 | BP | positive regulation of calcium ion transport | 118 | 1 | 0.0461 |
| GO:0097530 | BP | granulocyte migration | 132 | 1 | 0.0465 |
| GO:0051282 | BP | regulation of sequestering of calcium ion | 125 | 1 | 0.0468 |
| GO:0006887 | BP | exocytosis | 858 | 2 | 0.0469 |
| GO:0051208 | BP | sequestering of calcium ion | 128 | 1 | 0.0482 |
| GO:0098754 | BP | detoxification | 126 | 1 | 0.0490 |
| GO:1901343 | BP | negative regulation of vasculature development | 172 | 1 | 0.0491 |
| GO:0002576 | BP | platelet degranulation | 117 | 1 | 0.0492 |
| GO:2000736 | BP | regulation of stem cell differentiation | 114 | 1 | 0.0493 |

(B)

|  | **Description** | **N** | **DE** | **P.DE** |
| --- | --- | --- | --- | --- |
| path:hsa00982 | Drug metabolism - cytochrome P450 | 68 | 1 | 0.0147 |
| path:hsa05204 | Chemical carcinogenesis - DNA adducts | 67 | 1 | 0.0157 |
| path:hsa00980 | Metabolism of xenobiotics by cytochrome P450 | 76 | 1 | 0.0193 |
| path:hsa00480 | Glutathione metabolism | 55 | 1 | 0.0219 |
| path:hsa00983 | Drug metabolism - other enzymes | 79 | 1 | 0.0262 |
| path:hsa01524 | Platinum drug resistance | 71 | 1 | 0.0277 |
| path:hsa04061 | Viral protein interaction with cytokine and cytokine receptor | 95 | 1 | 0.0304 |

(C)

|  | **ONTOLOGY** | **TERM** | **N** | **DE** | **P.DE** |
| --- | --- | --- | --- | --- | --- |
| GO:0035634 | BP | response to stilbenoid | 6 | 1 | 0.0009 |
| GO:0036353 | BP | histone H2A-K119 monoubiquitination | 8 | 1 | 0.0045 |
| GO:0048842 | BP | positive regulation of axon extension involved in axon guidance | 7 | 1 | 0.0073 |
| GO:1902669 | BP | positive regulation of axon guidance | 8 | 1 | 0.0074 |
| GO:0009048 | BP | dosage compensation by inactivation of X chromosome | 16 | 1 | 0.0088 |
| GO:0009886 | BP | post-embryonic animal morphogenesis | 11 | 1 | 0.0092 |
| GO:0035518 | BP | histone H2A monoubiquitination | 15 | 1 | 0.0094 |
| GO:0007549 | BP | dosage compensation | 18 | 1 | 0.0104 |
| GO:0060219 | BP | camera-type eye photoreceptor cell differentiation | 19 | 1 | 0.0111 |
| GO:0006349 | BP | regulation of gene expression by genetic imprinting | 17 | 1 | 0.0119 |
| GO:0033522 | BP | histone H2A ubiquitination | 22 | 1 | 0.0130 |
| GO:0070593 | BP | dendrite self-avoidance | 16 | 1 | 0.0142 |
| GO:0010390 | BP | histone monoubiquitination | 27 | 1 | 0.0147 |
| GO:0071514 | BP | genetic imprinting | 26 | 1 | 0.0159 |
| GO:0038007 | BP | netrin-activated signaling pathway | 13 | 1 | 0.0164 |
| GO:0016574 | BP | histone ubiquitination | 41 | 1 | 0.0211 |
| GO:0010842 | BP | retina layer formation | 21 | 1 | 0.0229 |
| GO:0001754 | BP | eye photoreceptor cell differentiation | 46 | 1 | 0.0263 |
| GO:0048841 | BP | regulation of axon extension involved in axon guidance | 31 | 1 | 0.0273 |
| GO:0034260 | BP | negative regulation of GTPase activity | 44 | 1 | 0.0322 |
| GO:1902667 | BP | regulation of axon guidance | 43 | 1 | 0.0345 |
| GO:0045773 | BP | positive regulation of axon extension | 39 | 1 | 0.0346 |
| GO:0016266 | BP | O-glycan processing | 59 | 1 | 0.0348 |
| GO:0006513 | BP | protein monoubiquitination | 63 | 1 | 0.0349 |
| GO:0048846 | BP | axon extension involved in axon guidance | 36 | 1 | 0.0360 |
| GO:1902284 | BP | neuron projection extension involved in neuron projection guidance | 36 | 1 | 0.0360 |
| GO:0060042 | BP | retina morphogenesis in camera-type eye | 48 | 1 | 0.0362 |
| GO:0046530 | BP | photoreceptor cell differentiation | 62 | 1 | 0.0374 |
| GO:0043087 | BP | regulation of GTPase activity | 445 | 2 | 0.0428 |
| GO:0003407 | BP | neural retina development | 61 | 1 | 0.0429 |
| GO:0035773 | BP | insulin secretion involved in cellular response to glucose stimulus | 62 | 1 | 0.0443 |
| GO:0008038 | BP | neuron recognition | 48 | 1 | 0.0464 |

(D)

|  | **Description** | **N** | **DE** | **P.DE** |
| --- | --- | --- | --- | --- |
| path:hsa04940 | Type I diabetes mellitus | 41 | 1 | 0.0161 |
| path:hsa00514 | Other types of O-glycan biosynthesis | 45 | 1 | 0.0325 |

**Supplementary Table 11**. Gene set enrichment analysis result using male-specific significant DMRs associated with MEHHP concentration in maternal urine samples during late pregnancy (A: GO terms) and birth urine samples (B: GO terms, C: KEGG pathways).

(A)

|  | **ONTOLOGY** | **TERM** | **N** | **DE** | **P.DE** |
| --- | --- | --- | --- | --- | --- |
| GO:0048148 | BP | behavioral response to cocaine | 20 | 1 | 0.0048 |
| GO:1902287 | BP | semaphorin-plexin signaling pathway involved in axon guidance | 10 | 1 | 0.0076 |
| GO:1902285 | BP | semaphorin-plexin signaling pathway involved in neuron projection guidance | 11 | 1 | 0.0085 |
| GO:0010842 | BP | retina layer formation | 21 | 1 | 0.0111 |
| GO:0045332 | BP | phospholipid translocation | 24 | 1 | 0.0136 |
| GO:0034204 | BP | lipid translocation | 25 | 1 | 0.0136 |
| GO:0010975 | BP | regulation of neuron projection development | 478 | 2 | 0.0151 |
| GO:0042220 | BP | response to cocaine | 54 | 1 | 0.0160 |
| GO:0060042 | BP | retina morphogenesis in camera-type eye | 48 | 1 | 0.0183 |
| GO:0097035 | BP | regulation of membrane lipid distribution | 49 | 1 | 0.0193 |
| GO:0071526 | BP | semaphorin-plexin signaling pathway | 37 | 1 | 0.0204 |
| GO:0003407 | BP | neural retina development | 61 | 1 | 0.0211 |
| GO:0072347 | BP | response to anesthetic | 77 | 1 | 0.0229 |
| GO:0045664 | BP | regulation of neuron differentiation | 631 | 2 | 0.0230 |
| GO:0120035 | BP | regulation of plasma membrane bounded cell projection organization | 646 | 2 | 0.0237 |
| GO:0031344 | BP | regulation of cell projection organization | 656 | 2 | 0.0239 |
| GO:0015914 | BP | phospholipid transport | 74 | 1 | 0.0259 |
| GO:0015748 | BP | organophosphate ester transport | 100 | 1 | 0.0305 |
| GO:0010923 | BP | negative regulation of phosphatase activity | 100 | 1 | 0.0317 |
| GO:0050767 | BP | regulation of neurogenesis | 794 | 2 | 0.0324 |
| GO:0060998 | BP | regulation of dendritic spine development | 68 | 1 | 0.0330 |
| GO:0043279 | BP | response to alkaloid | 111 | 1 | 0.0343 |
| GO:0035305 | BP | negative regulation of dephosphorylation | 107 | 1 | 0.0350 |
| GO:0051960 | BP | regulation of nervous system development | 894 | 2 | 0.0392 |
| GO:0060284 | BP | regulation of cell development | 921 | 2 | 0.0397 |
| GO:0050772 | BP | positive regulation of axonogenesis | 79 | 1 | 0.0404 |
| GO:0060359 | BP | response to ammonium ion | 132 | 1 | 0.0418 |
| GO:0048593 | BP | camera-type eye morphogenesis | 110 | 1 | 0.0438 |
| GO:0060996 | BP | dendritic spine development | 88 | 1 | 0.0439 |
| GO:0051336 | BP | regulation of hydrolase activity | 1230 | 2 | 0.0443 |
| GO:0007156 | BP | homophilic cell adhesion via plasma membrane adhesion molecules | 162 | 1 | 0.0486 |
| GO:0030534 | BP | adult behavior | 140 | 1 | 0.0490 |

(B)

|  | **ONTOLOGY** | **TERM** | **N** | **DE** | **P.DE** |
| --- | --- | --- | --- | --- | --- |
| GO:0009249 | BP | protein lipoylation | 6 | 1 | 0.0013 |
| GO:0018065 | BP | protein-cofactor linkage | 10 | 1 | 0.0042 |
| GO:0017196 | BP | N-terminal peptidyl-methionine acetylation | 7 | 1 | 0.0046 |
| GO:0071895 | BP | odontoblast differentiation | 6 | 1 | 0.0055 |
| GO:0018206 | BP | peptidyl-methionine modification | 13 | 1 | 0.0068 |
| GO:0035090 | BP | maintenance of apical/basal cell polarity | 9 | 1 | 0.0079 |
| GO:0045199 | BP | maintenance of epithelial cell apical/basal polarity | 9 | 1 | 0.0079 |
| GO:0097187 | BP | dentinogenesis | 7 | 1 | 0.0088 |
| GO:0006474 | BP | N-terminal protein amino acid acetylation | 15 | 1 | 0.0097 |
| GO:0048842 | BP | positive regulation of axon extension involved in axon guidance | 7 | 1 | 0.0113 |
| GO:1902669 | BP | positive regulation of axon guidance | 8 | 1 | 0.0115 |
| GO:0009886 | BP | post-embryonic animal morphogenesis | 11 | 1 | 0.0136 |
| GO:0031365 | BP | N-terminal protein amino acid modification | 27 | 1 | 0.0149 |
| GO:0001886 | BP | endothelial cell morphogenesis | 12 | 1 | 0.0157 |
| GO:0030262 | BP | apoptotic nuclear changes | 34 | 1 | 0.0164 |
| GO:0060219 | BP | camera-type eye photoreceptor cell differentiation | 19 | 1 | 0.0167 |
| GO:0006921 | BP | cellular component disassembly involved in execution phase of apoptosis | 36 | 1 | 0.0176 |
| GO:0030011 | BP | maintenance of cell polarity | 16 | 1 | 0.0179 |
| GO:0051604 | BP | protein maturation | 318 | 2 | 0.0182 |
| GO:0070166 | BP | enamel mineralization | 16 | 1 | 0.0183 |
| GO:0036035 | BP | osteoclast development | 17 | 1 | 0.0208 |
| GO:0070593 | BP | dendrite self-avoidance | 16 | 1 | 0.0218 |
| GO:0038007 | BP | netrin-activated signaling pathway | 13 | 1 | 0.0242 |
| GO:0097186 | BP | amelogenesis | 24 | 1 | 0.0263 |
| GO:0034505 | BP | tooth mineralization | 22 | 1 | 0.0280 |
| GO:0018205 | BP | peptidyl-lysine modification | 370 | 2 | 0.0326 |
| GO:0040036 | BP | regulation of fibroblast growth factor receptor signaling pathway | 32 | 1 | 0.0329 |
| GO:0010842 | BP | retina layer formation | 21 | 1 | 0.0336 |
| GO:0001754 | BP | eye photoreceptor cell differentiation | 46 | 1 | 0.0393 |
| GO:0030501 | BP | positive regulation of bone mineralization | 38 | 1 | 0.0406 |
| GO:0043966 | BP | histone H3 acetylation | 57 | 1 | 0.0410 |
| GO:0098751 | BP | bone cell development | 34 | 1 | 0.0412 |
| GO:0003382 | BP | epithelial cell morphogenesis | 30 | 1 | 0.0420 |
| GO:0070169 | BP | positive regulation of biomineral tissue development | 45 | 1 | 0.0423 |
| GO:0048841 | BP | regulation of axon extension involved in axon guidance | 31 | 1 | 0.0424 |
| GO:0045197 | BP | establishment or maintenance of epithelial cell apical/basal polarity | 39 | 1 | 0.0477 |
| GO:0035088 | BP | establishment or maintenance of apical/basal cell polarity | 42 | 1 | 0.0495 |
| GO:0061245 | BP | establishment or maintenance of bipolar cell polarity | 42 | 1 | 0.0495 |
| GO:0060563 | BP | neuroepithelial cell differentiation | 53 | 1 | 0.0498 |

(C)

|  | **Description** | **N** | **DE** | **P.DE** |
| --- | --- | --- | --- | --- |
| path:hsa00514 | Other types of O-glycan biosynthesis | 45 | 1 | 0.0496 |

**Supplementary Table 12**. Gene set enrichment analysis result using male-specific significant DMRs associated with MnBP concentration in maternal urine samples during late pregnancy (A: GO terms, B: KEGG pathways) and birth urine samples (C: GO terms, D: KEGG pathways).

(A)

|  | **ONTOLOGY** | **TERM** | **N** | **DE** | **P.DE** |
| --- | --- | --- | --- | --- | --- |
| GO:0033145 | BP | positive regulation of intracellular steroid hormone receptor signaling pathway | 15 | 2 | 0.0000 |
| GO:0033143 | BP | regulation of intracellular steroid hormone receptor signaling pathway | 73 | 2 | 0.0005 |
| GO:0030518 | BP | intracellular steroid hormone receptor signaling pathway | 123 | 2 | 0.0013 |
| GO:0043401 | BP | steroid hormone mediated signaling pathway | 175 | 2 | 0.0029 |
| GO:0060315 | BP | negative regulation of ryanodine-sensitive calcium-release channel activity | 10 | 1 | 0.0030 |
| GO:0060316 | BP | positive regulation of ryanodine-sensitive calcium-release channel activity | 8 | 1 | 0.0032 |
| GO:0042178 | BP | xenobiotic catabolic process | 13 | 1 | 0.0035 |
| GO:0033148 | BP | positive regulation of intracellular estrogen receptor signaling pathway | 10 | 1 | 0.0038 |
| GO:0035360 | BP | positive regulation of peroxisome proliferator activated receptor signaling pathway | 9 | 1 | 0.0038 |
| GO:0009755 | BP | hormone-mediated signaling pathway | 224 | 2 | 0.0041 |
| GO:2000322 | BP | regulation of glucocorticoid receptor signaling pathway | 8 | 1 | 0.0042 |
| GO:0014819 | BP | regulation of skeletal muscle contraction | 12 | 1 | 0.0042 |
| GO:0090257 | BP | regulation of muscle system process | 251 | 2 | 0.0044 |
| GO:0061052 | BP | negative regulation of cell growth involved in cardiac muscle cell development | 12 | 1 | 0.0044 |
| GO:0051280 | BP | negative regulation of release of sequestered calcium ion into cytosol | 14 | 1 | 0.0044 |
| GO:0071383 | BP | cellular response to steroid hormone stimulus | 238 | 2 | 0.0047 |
| GO:1901685 | BP | glutathione derivative metabolic process | 22 | 1 | 0.0047 |
| GO:1901687 | BP | glutathione derivative biosynthetic process | 22 | 1 | 0.0047 |
| GO:0019852 | BP | L-ascorbic acid metabolic process | 10 | 1 | 0.0048 |
| GO:0060242 | BP | contact inhibition | 8 | 1 | 0.0051 |
| GO:0051284 | BP | positive regulation of sequestering of calcium ion | 16 | 1 | 0.0054 |
| GO:0030522 | BP | intracellular receptor signaling pathway | 265 | 2 | 0.0057 |
| GO:0035358 | BP | regulation of peroxisome proliferator activated receptor signaling pathway | 14 | 1 | 0.0057 |
| GO:0045989 | BP | positive regulation of striated muscle contraction | 15 | 1 | 0.0062 |
| GO:0071243 | BP | cellular response to arsenic-containing substance | 18 | 1 | 0.0063 |
| GO:0010523 | BP | negative regulation of calcium ion transport into cytosol | 18 | 1 | 0.0070 |
| GO:2000726 | BP | negative regulation of cardiac muscle cell differentiation | 21 | 1 | 0.0073 |
| GO:0042921 | BP | glucocorticoid receptor signaling pathway | 15 | 1 | 0.0074 |
| GO:0048012 | BP | hepatocyte growth factor receptor signaling pathway | 16 | 1 | 0.0079 |
| GO:0031958 | BP | corticosteroid receptor signaling pathway | 16 | 1 | 0.0080 |
| GO:0010763 | BP | positive regulation of fibroblast migration | 12 | 1 | 0.0081 |
| GO:0061050 | BP | regulation of cell growth involved in cardiac muscle cell development | 23 | 1 | 0.0084 |
| GO:0010881 | BP | regulation of cardiac muscle contraction  by regulation of the release of sequestered calcium ion | 22 | 1 | 0.0085 |
| GO:0048545 | BP | response to steroid hormone | 369 | 2 | 0.0092 |
| GO:0035357 | BP | peroxisome proliferator activated receptor signaling pathway | 20 | 1 | 0.0098 |
| GO:1905208 | BP | negative regulation of cardiocyte differentiation | 25 | 1 | 0.0101 |
| GO:0060314 | BP | regulation of ryanodine-sensitive calcium-release channel activity | 24 | 1 | 0.0107 |
| GO:0046628 | BP | positive regulation of insulin receptor signaling pathway | 22 | 1 | 0.0110 |
| GO:1904754 | BP | positive regulation of vascular associated smooth muscle cell migration | 23 | 1 | 0.0111 |
| GO:1901020 | BP | negative regulation of calcium ion transmembrane transporter activity | 34 | 1 | 0.0111 |
| GO:0010882 | BP | regulation of cardiac muscle contraction by calcium ion signaling | 27 | 1 | 0.0113 |
| GO:0050849 | BP | negative regulation of calcium-mediated signaling | 30 | 1 | 0.0119 |
| GO:0031116 | BP | positive regulation of microtubule polymerization | 28 | 1 | 0.0120 |
| GO:0046685 | BP | response to arsenic-containing substance | 31 | 1 | 0.0120 |
| GO:1900078 | BP | positive regulation of cellular response to insulin stimulus | 24 | 1 | 0.0121 |
| GO:0006874 | BP | cellular calcium ion homeostasis | 438 | 2 | 0.0123 |
| GO:0010880 | BP | regulation of release of sequestered calcium ion into cytosol  by sarcoplasmic reticulum | 30 | 1 | 0.0125 |
| GO:0055022 | BP | negative regulation of cardiac muscle tissue growth | 31 | 1 | 0.0126 |
| GO:0061117 | BP | negative regulation of heart growth | 31 | 1 | 0.0126 |
| GO:0055074 | BP | calcium ion homeostasis | 451 | 2 | 0.0131 |
| GO:0072503 | BP | cellular divalent inorganic cation homeostasis | 473 | 2 | 0.0135 |
| GO:0031112 | BP | positive regulation of microtubule polymerization or depolymerization | 32 | 1 | 0.0135 |
| GO:0090314 | BP | positive regulation of protein targeting to membrane | 29 | 1 | 0.0137 |
| GO:1903170 | BP | negative regulation of calcium ion transmembrane transport | 39 | 1 | 0.0138 |
| GO:0014808 | BP | release of sequestered calcium ion into cytosol by sarcoplasmic reticulum | 34 | 1 | 0.0139 |
| GO:1903514 | BP | release of sequestered calcium ion into cytosol by endoplasmic reticulum | 35 | 1 | 0.0140 |
| GO:0003298 | BP | physiological muscle hypertrophy | 35 | 1 | 0.0142 |
| GO:0003301 | BP | physiological cardiac muscle hypertrophy | 35 | 1 | 0.0142 |
| GO:0061049 | BP | cell growth involved in cardiac muscle cell development | 35 | 1 | 0.0142 |
| GO:0051281 | BP | positive regulation of release of sequestered calcium ion into cytosol | 40 | 1 | 0.0143 |
| GO:0003009 | BP | skeletal muscle contraction | 41 | 1 | 0.0144 |
| GO:0003012 | BP | muscle system process | 451 | 2 | 0.0144 |
| GO:0035722 | BP | interleukin-12-mediated signaling pathway | 44 | 1 | 0.0149 |
| GO:0072507 | BP | divalent inorganic cation homeostasis | 493 | 2 | 0.0150 |
| GO:0010614 | BP | negative regulation of cardiac muscle hypertrophy | 36 | 1 | 0.0150 |
| GO:0051154 | BP | negative regulation of striated muscle cell differentiation | 40 | 1 | 0.0151 |
| GO:0071349 | BP | cellular response to interleukin-12 | 46 | 1 | 0.0156 |
| GO:0090313 | BP | regulation of protein targeting to membrane | 33 | 1 | 0.0157 |
| GO:0070671 | BP | response to interleukin-12 | 47 | 1 | 0.0157 |
| GO:0014741 | BP | negative regulation of muscle hypertrophy | 38 | 1 | 0.0158 |
| GO:0055026 | BP | negative regulation of cardiac muscle tissue development | 41 | 1 | 0.0161 |
| GO:0070296 | BP | sarcoplasmic reticulum calcium ion transport | 40 | 1 | 0.0162 |
| GO:1904738 | BP | vascular associated smooth muscle cell migration | 39 | 1 | 0.0165 |
| GO:1904752 | BP | regulation of vascular associated smooth muscle cell migration | 39 | 1 | 0.0165 |
| GO:2001258 | BP | negative regulation of cation channel activity | 47 | 1 | 0.0166 |
| GO:0045933 | BP | positive regulation of muscle contraction | 47 | 1 | 0.0166 |
| GO:0033146 | BP | regulation of intracellular estrogen receptor signaling pathway | 38 | 1 | 0.0166 |
| GO:0010762 | BP | regulation of fibroblast migration | 29 | 1 | 0.0169 |
| GO:0006875 | BP | cellular metal ion homeostasis | 550 | 2 | 0.0173 |
| GO:0046621 | BP | negative regulation of organ growth | 38 | 1 | 0.0176 |
| GO:1902532 | BP | negative regulation of intracellular signal transduction | 538 | 2 | 0.0180 |
| GO:1904707 | BP | positive regulation of vascular smooth muscle cell proliferation | 46 | 1 | 0.0181 |
| GO:0050850 | BP | positive regulation of calcium-mediated signaling | 43 | 1 | 0.0184 |
| GO:2000725 | BP | regulation of cardiac muscle cell differentiation | 47 | 1 | 0.0184 |
| GO:1901021 | BP | positive regulation of calcium ion transmembrane transporter activity | 37 | 1 | 0.0185 |
| GO:0031295 | BP | T cell costimulation | 51 | 1 | 0.0192 |
| GO:0010524 | BP | positive regulation of calcium ion transport into cytosol | 54 | 1 | 0.0194 |
| GO:0031294 | BP | lymphocyte costimulation | 52 | 1 | 0.0195 |
| GO:0071407 | BP | cellular response to organic cyclic compound | 521 | 2 | 0.0199 |
| GO:0050879 | BP | multicellular organismal movement | 54 | 1 | 0.0211 |
| GO:0050881 | BP | musculoskeletal movement | 54 | 1 | 0.0211 |
| GO:0071396 | BP | cellular response to lipid | 588 | 2 | 0.0212 |
| GO:0010761 | BP | fibroblast migration | 37 | 1 | 0.0212 |
| GO:0051926 | BP | negative regulation of calcium ion transport | 63 | 1 | 0.0212 |
| GO:0030003 | BP | cellular cation homeostasis | 619 | 2 | 0.0212 |
| GO:0014911 | BP | positive regulation of smooth muscle cell migration | 43 | 1 | 0.0213 |
| GO:0031113 | BP | regulation of microtubule polymerization | 49 | 1 | 0.0217 |
| GO:0006873 | BP | cellular ion homeostasis | 632 | 2 | 0.0221 |
| GO:0009967 | BP | positive regulation of signal transduction | 1621 | 3 | 0.0222 |
| GO:0045843 | BP | negative regulation of striated muscle tissue development | 57 | 1 | 0.0227 |
| GO:0030520 | BP | intracellular estrogen receptor signaling pathway | 54 | 1 | 0.0228 |
| GO:0055065 | BP | metal ion homeostasis | 622 | 2 | 0.0229 |
| GO:0048635 | BP | negative regulation of muscle organ development | 58 | 1 | 0.0234 |
| GO:0045773 | BP | positive regulation of axon extension | 39 | 1 | 0.0242 |
| GO:0051148 | BP | negative regulation of muscle cell differentiation | 60 | 1 | 0.0244 |
| GO:1901862 | BP | negative regulation of muscle tissue development | 60 | 1 | 0.0246 |
| GO:0045600 | BP | positive regulation of fat cell differentiation | 62 | 1 | 0.0248 |
| GO:0051496 | BP | positive regulation of stress fiber assembly | 51 | 1 | 0.0255 |
| GO:0044057 | BP | regulation of system process | 589 | 2 | 0.0258 |
| GO:1905207 | BP | regulation of cardiocyte differentiation | 63 | 1 | 0.0263 |
| GO:1904705 | BP | regulation of vascular smooth muscle cell proliferation | 76 | 1 | 0.0273 |
| GO:1990874 | BP | vascular smooth muscle cell proliferation | 76 | 1 | 0.0273 |
| GO:0055080 | BP | cation homeostasis | 695 | 2 | 0.0277 |
| GO:1904427 | BP | positive regulation of calcium ion transmembrane transport | 69 | 1 | 0.0284 |
| GO:2001259 | BP | positive regulation of cation channel activity | 69 | 1 | 0.0284 |
| GO:0098771 | BP | inorganic ion homeostasis | 706 | 2 | 0.0284 |
| GO:0044093 | BP | positive regulation of molecular function | 1685 | 3 | 0.0285 |
| GO:0098869 | BP | cellular oxidant detoxification | 97 | 1 | 0.0285 |
| GO:0032233 | BP | positive regulation of actin filament bundle assembly | 60 | 1 | 0.0288 |
| GO:0070373 | BP | negative regulation of ERK1 and ERK2 cascade | 70 | 1 | 0.0288 |
| GO:0010647 | BP | positive regulation of cell communication | 1775 | 3 | 0.0291 |
| GO:0046626 | BP | regulation of insulin receptor signaling pathway | 65 | 1 | 0.0294 |
| GO:0006767 | BP | water-soluble vitamin metabolic process | 84 | 1 | 0.0295 |
| GO:0023056 | BP | positive regulation of signaling | 1782 | 3 | 0.0295 |
| GO:0032413 | BP | negative regulation of ion transmembrane transporter activity | 73 | 1 | 0.0300 |
| GO:1990748 | BP | cellular detoxification | 107 | 1 | 0.0309 |
| GO:0031110 | BP | regulation of microtubule polymerization or depolymerization | 74 | 1 | 0.0310 |
| GO:0010611 | BP | regulation of cardiac muscle hypertrophy | 73 | 1 | 0.0311 |
| GO:0055021 | BP | regulation of cardiac muscle tissue growth | 72 | 1 | 0.0314 |
| GO:1903533 | BP | regulation of protein targeting | 77 | 1 | 0.0316 |
| GO:0032870 | BP | cellular response to hormone stimulus | 673 | 2 | 0.0316 |
| GO:0014743 | BP | regulation of muscle hypertrophy | 76 | 1 | 0.0320 |
| GO:0046785 | BP | microtubule polymerization | 73 | 1 | 0.0322 |
| GO:1902531 | BP | regulation of intracellular signal transduction | 1870 | 3 | 0.0322 |
| GO:1900076 | BP | regulation of cellular response to insulin stimulus | 73 | 1 | 0.0323 |
| GO:0043408 | BP | regulation of MAPK cascade | 735 | 2 | 0.0325 |
| GO:0050801 | BP | ion homeostasis | 772 | 2 | 0.0335 |
| GO:0006805 | BP | xenobiotic metabolic process | 124 | 1 | 0.0335 |
| GO:0032410 | BP | negative regulation of transporter activity | 84 | 1 | 0.0338 |
| GO:0055117 | BP | regulation of cardiac muscle contraction | 77 | 1 | 0.0340 |
| GO:0098754 | BP | detoxification | 126 | 1 | 0.0341 |
| GO:0055082 | BP | cellular chemical homeostasis | 775 | 2 | 0.0341 |
| GO:1904063 | BP | negative regulation of cation transmembrane transport | 86 | 1 | 0.0343 |
| GO:0051279 | BP | regulation of release of sequestered calcium ion into cytosol | 80 | 1 | 0.0347 |
| GO:0043507 | BP | positive regulation of JUN kinase activity | 70 | 1 | 0.0349 |
| GO:0060420 | BP | regulation of heart growth | 78 | 1 | 0.0351 |
| GO:0014910 | BP | regulation of smooth muscle cell migration | 76 | 1 | 0.0353 |
| GO:0002223 | BP | stimulatory C-type lectin receptor signaling pathway | 108 | 1 | 0.0371 |
| GO:0002220 | BP | innate immune response activating cell surface receptor signaling pathway | 111 | 1 | 0.0374 |
| GO:0014909 | BP | smooth muscle cell migration | 83 | 1 | 0.0384 |
| GO:1901019 | BP | regulation of calcium ion transmembrane transporter activity | 84 | 1 | 0.0389 |
| GO:0006942 | BP | regulation of striated muscle contraction | 91 | 1 | 0.0390 |
| GO:0055013 | BP | cardiac muscle cell development | 86 | 1 | 0.0392 |
| GO:0048661 | BP | positive regulation of smooth muscle cell proliferation | 96 | 1 | 0.0396 |
| GO:0043506 | BP | regulation of JUN kinase activity | 84 | 1 | 0.0398 |
| GO:0034766 | BP | negative regulation of ion transmembrane transport | 97 | 1 | 0.0405 |
| GO:0051492 | BP | regulation of stress fiber assembly | 81 | 1 | 0.0406 |
| GO:0050848 | BP | regulation of calcium-mediated signaling | 94 | 1 | 0.0408 |
| GO:0055024 | BP | regulation of cardiac muscle tissue development | 94 | 1 | 0.0412 |
| GO:0002433 | BP | immune response-regulating cell surface receptor signaling pathway  involved in phagocytosis | 77 | 1 | 0.0413 |
| GO:0038096 | BP | Fc-gamma receptor signaling pathway involved in phagocytosis | 77 | 1 | 0.0413 |
| GO:0033138 | BP | positive regulation of peptidyl-serine phosphorylation | 98 | 1 | 0.0415 |
| GO:0038094 | BP | Fc-gamma receptor signaling pathway | 80 | 1 | 0.0417 |
| GO:0032414 | BP | positive regulation of ion transmembrane transporter activity | 99 | 1 | 0.0423 |
| GO:0033993 | BP | response to lipid | 891 | 2 | 0.0424 |
| GO:0055006 | BP | cardiac cell development | 92 | 1 | 0.0427 |
| GO:0031109 | BP | microtubule polymerization or depolymerization | 106 | 1 | 0.0431 |
| GO:0010522 | BP | regulation of calcium ion transport into cytosol | 100 | 1 | 0.0431 |
| GO:0014812 | BP | muscle cell migration | 96 | 1 | 0.0433 |
| GO:0006766 | BP | vitamin metabolic process | 130 | 1 | 0.0434 |
| GO:0043502 | BP | regulation of muscle adaptation | 105 | 1 | 0.0435 |
| GO:0002431 | BP | Fc receptor mediated stimulatory signaling pathway | 83 | 1 | 0.0437 |
| GO:0021549 | BP | cerebellum development | 95 | 1 | 0.0438 |
| GO:0055017 | BP | cardiac muscle tissue growth | 98 | 1 | 0.0444 |
| GO:0110020 | BP | regulation of actomyosin structure organization | 92 | 1 | 0.0447 |
| GO:0014070 | BP | response to organic cyclic compound | 878 | 2 | 0.0452 |
| GO:0038095 | BP | Fc-epsilon receptor signaling pathway | 107 | 1 | 0.0454 |
| GO:0051928 | BP | positive regulation of calcium ion transport | 118 | 1 | 0.0455 |
| GO:0006612 | BP | protein targeting to membrane | 185 | 1 | 0.0457 |
| GO:0003300 | BP | cardiac muscle hypertrophy | 103 | 1 | 0.0457 |
| GO:0032411 | BP | positive regulation of transporter activity | 107 | 1 | 0.0459 |
| GO:0019725 | BP | cellular homeostasis | 928 | 2 | 0.0460 |
| GO:0051153 | BP | regulation of striated muscle cell differentiation | 113 | 1 | 0.0461 |
| GO:0014897 | BP | striated muscle hypertrophy | 106 | 1 | 0.0465 |
| GO:0048013 | BP | ephrin receptor signaling pathway | 84 | 1 | 0.0466 |
| GO:0032231 | BP | regulation of actin filament bundle assembly | 93 | 1 | 0.0468 |
| GO:0014896 | BP | muscle hypertrophy | 108 | 1 | 0.0469 |
| GO:0046620 | BP | regulation of organ growth | 104 | 1 | 0.0474 |
| GO:0043500 | BP | muscle adaptation | 120 | 1 | 0.0476 |
| GO:0022037 | BP | metencephalon development | 104 | 1 | 0.0476 |
| GO:0034763 | BP | negative regulation of transmembrane transport | 122 | 1 | 0.0477 |
| GO:0060419 | BP | heart growth | 105 | 1 | 0.0480 |
| GO:0050772 | BP | positive regulation of axonogenesis | 79 | 1 | 0.0485 |
| GO:0000165 | BP | MAPK cascade | 914 | 2 | 0.0489 |
| GO:0048584 | BP | positive regulation of response to stimulus | 2292 | 3 | 0.0492 |
| GO:0030038 | BP | contractile actin filament bundle assembly | 95 | 1 | 0.0494 |
| GO:0043149 | BP | stress fiber assembly | 95 | 1 | 0.0494 |

(B)

|  | **Description** | **N** | **DE** | **P.DE** |
| --- | --- | --- | --- | --- |
| path:hsa05204 | Chemical carcinogenesis - DNA adducts | 67 | 1 | 0.0122 |
| path:hsa00982 | Drug metabolism - cytochrome P450 | 68 | 1 | 0.0124 |
| path:hsa00480 | Glutathione metabolism | 55 | 1 | 0.0134 |
| path:hsa00980 | Metabolism of xenobiotics by cytochrome P450 | 76 | 1 | 0.0138 |
| path:hsa04392 | Hippo signaling pathway - multiple species | 29 | 1 | 0.0178 |
| path:hsa00983 | Drug metabolism - other enzymes | 79 | 1 | 0.0187 |
| path:hsa01524 | Platinum drug resistance | 71 | 1 | 0.0263 |
| path:hsa05120 | Epithelial cell signaling in Helicobacter pylori infection | 68 | 1 | 0.0277 |
| path:hsa05211 | Renal cell carcinoma | 65 | 1 | 0.0334 |
| path:hsa04650 | Natural killer cell mediated cytotoxicity | 118 | 1 | 0.0438 |
| path:hsa04625 | C-type lectin receptor signaling pathway | 103 | 1 | 0.0440 |
| path:hsa04012 | ErbB signaling pathway | 81 | 1 | 0.0474 |
| path:hsa04660 | T cell receptor signaling pathway | 99 | 1 | 0.0479 |
| path:hsa04666 | Fc gamma R-mediated phagocytosis | 95 | 1 | 0.0482 |

(C)

|  | **ONTOLOGY** | **TERM** | **N** | **DE** | **P.DE** |
| --- | --- | --- | --- | --- | --- |
| GO:0009838 | BP | abscission | 6 | 1 | 0.0014 |
| GO:0032466 | BP | negative regulation of cytokinesis | 7 | 1 | 0.0017 |
| GO:0055064 | BP | chloride ion homeostasis | 14 | 1 | 0.0031 |
| GO:0051782 | BP | negative regulation of cell division | 18 | 1 | 0.0044 |
| GO:0055083 | BP | monovalent inorganic anion homeostasis | 26 | 1 | 0.0059 |
| GO:0006884 | BP | cell volume homeostasis | 27 | 1 | 0.0066 |
| GO:0055075 | BP | potassium ion homeostasis | 29 | 1 | 0.0066 |
| GO:1990573 | BP | potassium ion import across plasma membrane | 43 | 1 | 0.0094 |
| GO:1901998 | BP | toxin transport | 39 | 1 | 0.0096 |
| GO:0010107 | BP | potassium ion import | 47 | 1 | 0.0104 |
| GO:0055081 | BP | anion homeostasis | 56 | 1 | 0.0132 |
| GO:0008088 | BP | axo-dendritic transport | 67 | 1 | 0.0170 |
| GO:1902476 | BP | chloride transmembrane transport | 80 | 1 | 0.0181 |
| GO:0098659 | BP | inorganic cation import across plasma membrane | 80 | 1 | 0.0187 |
| GO:0099587 | BP | inorganic ion import across plasma membrane | 80 | 1 | 0.0187 |
| GO:0032465 | BP | regulation of cytokinesis | 83 | 1 | 0.0196 |
| GO:0010972 | BP | negative regulation of G2/M transition of mitotic cell cycle | 89 | 1 | 0.0216 |
| GO:0006821 | BP | chloride transport | 99 | 1 | 0.0223 |
| GO:0098661 | BP | inorganic anion transmembrane transport | 102 | 1 | 0.0229 |
| GO:1902750 | BP | negative regulation of cell cycle G2/M phase transition | 100 | 1 | 0.0242 |
| GO:0098739 | BP | import across plasma membrane | 106 | 1 | 0.0246 |
| GO:0055067 | BP | monovalent inorganic cation homeostasis | 147 | 1 | 0.0346 |
| GO:0015698 | BP | inorganic anion transport | 158 | 1 | 0.0355 |
| GO:0010970 | BP | transport along microtubule | 155 | 1 | 0.0375 |
| GO:0099111 | BP | microtubule-based transport | 155 | 1 | 0.0375 |
| GO:0051302 | BP | regulation of cell division | 160 | 1 | 0.0377 |
| GO:0000910 | BP | cytokinesis | 163 | 1 | 0.0383 |
| GO:0007093 | BP | mitotic cell cycle checkpoint | 164 | 1 | 0.0389 |
| GO:0008361 | BP | regulation of cell size | 169 | 1 | 0.0401 |
| GO:0030705 | BP | cytoskeleton-dependent intracellular transport | 174 | 1 | 0.0419 |
| GO:0031424 | BP | keratinization | 219 | 1 | 0.0429 |
| GO:0010389 | BP | regulation of G2/M transition of mitotic cell cycle | 187 | 1 | 0.0443 |
| GO:0071804 | BP | cellular potassium ion transport | 209 | 1 | 0.0474 |
| GO:0071805 | BP | potassium ion transmembrane transport | 209 | 1 | 0.0474 |
| GO:1902749 | BP | regulation of cell cycle G2/M phase transition | 203 | 1 | 0.0481 |

(D)

|  | **Description** | **N** | **DE** | **P.DE** |
| --- | --- | --- | --- | --- |
| path:hsa04966 | Collecting duct acid secretion | 27 | 1 | 0.0063 |

**Supplementary Table 13**. Gene set enrichment analysis result using male-specific significant DMRs associated with DEHP concentration in maternal urine samples during late pregnancy (A: GO terms) and birth urine samples (B: GO terms, C: KEGG pathways).

(A)

|  | **ONTOLOGY** | **TERM** | **N** | **DE** | **P.DE** |
| --- | --- | --- | --- | --- | --- |
| GO:0034334 | BP | adherens junction maintenance | 6 | 1 | 0.0026 |
| GO:0034331 | BP | cell junction maintenance | 14 | 1 | 0.0053 |
| GO:2000738 | BP | positive regulation of stem cell differentiation | 21 | 1 | 0.0083 |
| GO:0045332 | BP | phospholipid translocation | 24 | 1 | 0.0107 |
| GO:0034204 | BP | lipid translocation | 25 | 1 | 0.0110 |
| GO:0097035 | BP | regulation of membrane lipid distribution | 49 | 1 | 0.0191 |
| GO:0043954 | BP | cellular component maintenance | 53 | 1 | 0.0204 |
| GO:0006305 | BP | DNA alkylation | 70 | 1 | 0.0241 |
| GO:0006306 | BP | DNA methylation | 70 | 1 | 0.0241 |
| GO:0015914 | BP | phospholipid transport | 74 | 1 | 0.0259 |
| GO:0044728 | BP | DNA methylation or demethylation | 91 | 1 | 0.0288 |
| GO:0060218 | BP | hematopoietic stem cell differentiation | 81 | 1 | 0.0299 |
| GO:0015748 | BP | organophosphate ester transport | 100 | 1 | 0.0350 |
| GO:0006304 | BP | DNA modification | 113 | 1 | 0.0357 |
| GO:0010923 | BP | negative regulation of phosphatase activity | 100 | 1 | 0.0365 |
| GO:0035305 | BP | negative regulation of dephosphorylation | 107 | 1 | 0.0393 |
| GO:2000736 | BP | regulation of stem cell differentiation | 114 | 1 | 0.0428 |

(B)

|  | **ONTOLOGY** | **TERM** | **N** | **DE** | **P.DE** |
| --- | --- | --- | --- | --- | --- |
| GO:0035634 | BP | response to stilbenoid | 6 | 1 | 0.0015 |
| GO:0036353 | BP | histone H2A-K119 monoubiquitination | 8 | 1 | 0.0052 |
| GO:0048842 | BP | positive regulation of axon extension involved in axon guidance | 7 | 1 | 0.0079 |
| GO:1902669 | BP | positive regulation of axon guidance | 8 | 1 | 0.0080 |
| GO:0009886 | BP | post-embryonic animal morphogenesis | 11 | 1 | 0.0098 |
| GO:0009048 | BP | dosage compensation by inactivation of X chromosome | 16 | 1 | 0.0101 |
| GO:0035518 | BP | histone H2A monoubiquitination | 15 | 1 | 0.0105 |
| GO:0001886 | BP | endothelial cell morphogenesis | 12 | 1 | 0.0114 |
| GO:0007549 | BP | dosage compensation | 18 | 1 | 0.0120 |
| GO:0060219 | BP | camera-type eye photoreceptor cell differentiation | 19 | 1 | 0.0120 |
| GO:0006349 | BP | regulation of gene expression by genetic imprinting | 17 | 1 | 0.0132 |
| GO:0033522 | BP | histone H2A ubiquitination | 22 | 1 | 0.0147 |
| GO:0070593 | BP | dendrite self-avoidance | 16 | 1 | 0.0154 |
| GO:0010390 | BP | histone monoubiquitination | 27 | 1 | 0.0164 |
| GO:0038007 | BP | netrin-activated signaling pathway | 13 | 1 | 0.0171 |
| GO:0071514 | BP | genetic imprinting | 26 | 1 | 0.0174 |
| GO:0010842 | BP | retina layer formation | 21 | 1 | 0.0235 |
| GO:0016574 | BP | histone ubiquitination | 41 | 1 | 0.0238 |
| GO:0001754 | BP | eye photoreceptor cell differentiation | 46 | 1 | 0.0291 |
| GO:0048841 | BP | regulation of axon extension involved in axon guidance | 31 | 1 | 0.0300 |
| GO:0003382 | BP | epithelial cell morphogenesis | 30 | 1 | 0.0301 |
| GO:0016266 | BP | O-glycan processing | 59 | 1 | 0.0374 |
| GO:0045773 | BP | positive regulation of axon extension | 39 | 1 | 0.0377 |
| GO:1902667 | BP | regulation of axon guidance | 43 | 1 | 0.0385 |
| GO:0048846 | BP | axon extension involved in axon guidance | 36 | 1 | 0.0387 |
| GO:1902284 | BP | neuron projection extension involved in neuron projection guidance | 36 | 1 | 0.0387 |
| GO:0060042 | BP | retina morphogenesis in camera-type eye | 48 | 1 | 0.0389 |
| GO:0006513 | BP | protein monoubiquitination | 63 | 1 | 0.0392 |
| GO:0046530 | BP | photoreceptor cell differentiation | 62 | 1 | 0.0408 |
| GO:0003407 | BP | neural retina development | 61 | 1 | 0.0462 |
| GO:0001885 | BP | endothelial cell development | 56 | 1 | 0.0463 |
| GO:0008038 | BP | neuron recognition | 48 | 1 | 0.0492 |

(C)

|  | **Description** | **N** | **DE** | **P.DE** |
| --- | --- | --- | --- | --- |
| path:hsa00514 | Other types of O-glycan biosynthesis | 45 | 1 | 0.0355 |
